# Pharmacogenetic meta-analysis of baseline risk factors, pharmacodynamic, efficacy and tolerability endpoints from two large global cardiovascular outcomes trials for darapladib

**DOI:** 10.1371/journal.pone.0182115

**Published:** 2017-07-28

**Authors:** Astrid Yeo, Li Li, Liling Warren, Jennifer Aponte, Dana Fraser, Karen King, Kelley Johansson, Allison Barnes, Colin MacPhee, Richard Davies, Stephanie Chissoe, Elizabeth Tarka, Michelle L. O’Donoghue, Harvey D. White, Lars Wallentin, Dawn Waterworth

**Affiliations:** 1 Department of Genetics, GlaxoSmithKline Medicines Research Centre, Stevenage, Hertfordshire, United Kingdom; 2 Department of Genetics, GlaxoSmithKline Medicines Research Centre, Research Triangle Park, North Carolina, United States of America; 3 Clinical Statistics, GlaxoSmithKline Medicines Research Centre, Research Triangle Park, North Carolina, United States of America; 4 Department of Vascular Biology & Thrombosis, GlaxoSmithKline Medicines Research Centre, King of Prussia, Pennsylvania, United States of America; 5 Metabolic Pathways and Cardiovascular Therapeutic Area, GlaxoSmithKline Medicines Research Centre, King of Prussia, Pennsylvania, United States of America; 6 TIMI Study Group, Cardiovascular Division, Brigham and Women’s Hospital, Boston, Massachusetts, United States of America; 7 Green Lane Cardiovascular Service, Auckland City Hospital and University of Auckland, Auckland, New Zealand; 8 Department of Medical Sciences, Cardiology & Uppsala Clinical Research Center, Uppsala University, Uppsala, Sweden; 9 Department of Genetics, GlaxoSmithKline Medicines Research Centre, Upper Merion, Philadelphia, Pennsylvania, United States of America; Universita degli Studi di Milano, ITALY

## Abstract

Darapladib, a lipoprotein-associated phospholipase A2 (Lp-PLA_2_) inhibitor, failed to demonstrate efficacy for the primary endpoints in two large phase III cardiovascular outcomes trials, one in stable coronary heart disease patients (STABILITY) and one in acute coronary syndrome (SOLID-TIMI 52). No major safety signals were observed but tolerability issues of diarrhea and odor were common (up to 13%). We hypothesized that genetic variants associated with Lp-PLA_2_ activity may influence efficacy and tolerability and therefore performed a comprehensive pharmacogenetic analysis of both trials. We genotyped patients within the STABILITY and SOLID-TIMI 52 trials who provided a DNA sample and consent (n = 13,577 and 10,404 respectively, representing 86% and 82% of the trial participants) using genome-wide arrays with exome content and performed imputation using a 1000 Genomes reference panel. We investigated baseline and change from baseline in Lp-PLA_2_ activity, two efficacy endpoints (major coronary events and myocardial infarction) as well as tolerability parameters at genome-wide and candidate gene level using a meta-analytic approach. We replicated associations of published loci on baseline Lp-PLA_2_ activity (*APOE*, *CELSR2*, *LPA*, *PLA2G7*, *LDLR* and *SCARB1*) and identified three novel loci (*TOMM5*, *FRMD5* and *LPL*) using the GWAS-significance threshold P≤5E-08. Review of the *PLA2G7* gene (encoding Lp-PLA_2_) within these datasets identified V279F null allele carriers as well as three other rare exonic null alleles within various ethnic groups, however none of these variants nor any other loci associated with Lp-PLA_2_ activity at baseline were associated with any of the drug response endpoints. The analysis of darapladib efficacy endpoints, despite low power, identified six low frequency loci with main genotype effect (though with borderline imputation scores) and one common locus (minor allele frequency 0.24) with genotype by treatment interaction effect passing the GWAS-significance threshold. This locus conferred risk in placebo subjects, hazard ratio (HR) 1.22 with 95% confidence interval (CI) 1.11–1.33, but was protective in darapladib subjects, HR 0.79 (95% CI 0.71–0.88). No major loci for tolerability were found. Thus, genetic analysis confirmed and extended the influence of lipoprotein loci on Lp-PLA_2_ levels, identified some novel null alleles in the *PLA2G7* gene, and only identified one potentially efficacious subgroup within these two large clinical trials.

## Introduction

Lipoprotein-associated phospholipase A2 (Lp-PLA_2_) is the product of activated inflammatory cells with circulating Lp-PLA_2_ predominantly bound to low-density lipoprotein (LDL) cholesterol [1]. Elevated plasma levels of Lp-PLA_2_ are associated with an increased risk of cardiovascular (CV) events, such as coronary heart disease (CHD) death, myocardial infarction (MI) and stroke [2,3]. High expression of Lp-PLA_2_ has been noted in human atherosclerotic lesions, particularly in thin cap fibroatheroma or ruptured plaques [4,5]. In these high risk lesions that often lead to sudden death or MI, the enzyme is highly localized to activated macrophages undergoing apoptosis in the lipid necrotic core and fibrous cap suggesting a potential role in promoting plaque instability [5]. Therefore, it had been proposed that direct inhibition of the pro-inflammatory Lp-PLA_2_ enzyme may reduce vascular inflammation and promote stability of vulnerable plaques. The IBIS-2 study demonstrated (using intravascular ultrasound virtual histology) that darapladib could halt the growth of the plaque necrotic core volume (a secondary endpoint) over 12 months in CHD patients, further supporting this hypothesis, though there were no significant differences in the primary endpoints of plaque deformability or plasma high-sensitivity C-reactive protein [6]. A reduction of plaque necrotic core area and reduction in medial destruction had also previously been observed in hypercholesteremic swine treated with darapladib [7]. Few human genetic data was available to support target validation of Lp-PLA_2_ prior to the Phase III trials commencing.

Darapladib (SB-480848) is a novel, selective, reversible, orally active inhibitor of Lp-PLA_2_ activity that was in development by GlaxoSmithKline (GSK) for CV risk reduction. GSK conducted two phase III outcome studies investigating the clinical efficacy of long-term treatment with darapladib enteric coated (EC) tablets, 160 mg (oral once daily dose) as compared to placebo when added to standard of care. It was hypothesized that direct inhibition of Lp-PLA_2_ activity with darapladib may reduce residual cardiovascular events in high risk individuals when given as an adjunct to standard of care, including lipid-lowering and anti-platelet therapies [1;3;8–10]. Each trial was randomized, double-blind, placebo-controlled, multicenter, and event-driven with approximately 1,500 major adverse cardiovascular events (MACE: first occurrence of non-fatal stroke, non-fatal MI or CV death). The first study was STABILITY (STabilisation of Atherosclerotic plaque By Initiation of darapLadIb TherapY; NCT00799903), which focused on darapladib’s effect on the incidence of MACE in 15,828 subjects with chronic coronary heart disease (CHD) [11]. The SOLID-TIMI 52 (The Stabilization Of pLaques usIng Darapladib-Thrombolysis In Myocardial Infarction 52) trial, evaluated the effects of darapladib on the incidence of major coronary events (MCE: CHD death, non-fatal MI or urgent revascularization for myocardial ischemia) in 13,026 subjects within 30 days of hospitalization with an acute coronary syndrome (ACS) (NCT01000727)[12]. The primary endpoint of the SOLID-TIMI 52 trial was changed from MACE to MCE prior to study unblinding, following the availability of the results of the STABILITY trial. Neither of the trials showed statistically significant results for the primary efficacy endpoints [13;14]. However, there was a potential signal of efficacy in the STABILITY trial where nominally significant reductions were observed for the MCE HR 0.90; 95% CI, 0.82 to 1.00; p = 0.045) and total coronary events endpoints (CHD death, non-fatal MI, hospitalization for unstable angina, or any coronary revascularization procedure; HR 0.91; 95% CI, 0.84 to 0.98; p = 0.02) [14], but not in the SOLID-TIMI 52 trial [13]. Both trials observed a higher percentage of patients experiencing diarrhea (darapladib treated vs placebo: 12% vs 6% in STABILITY and 11% vs 6% in SOLID-TIMI 52), odor (darapladib treated vs placebo: 13% vs 2% in STABILITY and 12% vs 2% in SOLID-TIMI 52) but no consistent, serious adverse events were observed.

A natural deficiency in Lp-PLA_2_ is present in East Asian populations due to a genetic null variant (V279F; rs76863441)[15] and as a result subjects who carry two copies of this genetic null variant have absent or very low levels of enzyme activity in plasma. Therefore subjects with both parents of Japanese, Chinese or Korean ancestry were screened and those with Lp-PLA_2_ activity levels ≤20 nmol/min/mL (indicative of 279FF subjects) were excluded from enrollment into the STABILITY and SOLID-TIMI 52 studies as there would be no clear rationale for exposing such subjects to darapladib. Attempts to use this variant to predict CHD risk have provided mixed results, with some meta-analyses suggesting no relationship [16,17] and a study in Koreans suggesting risk reduction, but only in men with MI or angiographically defined disease [18]. A more recent study in China provided robust evidence of a lack of association with stroke (concordant with results from both darapladib trials) though it was under-powered for coronary heart disease [19]. Null alleles are rare in other ethnic groups as evidenced by a very recent meta-analysis in a multi-ethnic study, designed to test the association of *PLA2G7* variants and CHD [20]. In over 180,000 subjects of European or South Asian descent, less than 40 carriers of null alleles were found. No significant associations with CHD were observed with either these null variants or the V279F in an East Asian sample (~10K cases and ~15K controls), with point estimates broadly concordant with the clinical trial results.

Several genome-wide association studies (GWAS) of Lp-PLA_2_ activity levels and mass were performed (Framingham Heart Study [FHS][21]; Cohorts for Heart and Aging Research in Genomic Epidemiology [CHARGE][22]; Justification for the Use of statins in Primary prevention: an Intervention Trial Evaluating Rosuvastatin [JUPITER][23]). The majority of the loci that showed genome-wide association contained genetic variants that are known to be involved in lipid metabolism and have been observed to strongly influence circulating levels of LDL-C and/or HDL-C [22].

We hypothesized that genetic variants may also influence response to darapladib. As the STABILITY and SOLID-TIMI 52 trials were so large (>24,000 patients combined), we conducted genotyping ahead of time so that the data would be available contemporaneously with the clinical data upon trial completion. In order to maximize the power we performed meta-analyses of the STABILITY and SOLID-TIMI 52 trials for efficacy, pharmacodynamic and tolerability endpoints. The efficacy analysis was focused on MCE and MI endpoints, as those phenotypes had greater efficacy within STABILITY as compared to others. We also investigated baseline and change from baseline Lp-PLA_2_ activity levels. Baseline Lp-PLA_2_ activity levels were associated with primary and secondary endpoints in STABILITY [24], but not in SOLID-TIMI 52. We used both a genome-wide and candidate gene approach, with the candidate genes including those known to influence Lp-PLA_2_ activity levels and darapladib absorption, distribution, metabolism and excretion (ADME). The results reported here represent the one of largest pharmacogenetic (PGx) studies of cardiovascular treatment endpoints reported to date and also contain exome chip variants which have not been included in many PGx evaluations to date. We found genetic variation influencing baseline Lp-PLA_2_ activity levels, but those variants did not influence outcomes. Despite the large size of the clinical trials and availability of a good pharmacodynamic marker, we found just one common locus that appeared to influence cardiovascular outcomes.

## Results

Sample size, demographics, and laboratory characteristics of each endpoint by study are presented in Table 1. Although most characteristics were similar for the STABILITY and SOLID-TIMI 52 trials, there were considerable differences in the use of P2Y12 inhibitors (34% vs 89% respectively) and the prevalence of chronic kidney disease (30% vs 17% respectively), which may be explained, at least in part, by the different study design with regard to disease characteristics (chronic vs acute).

**Table 1:** Characteristics of PGx study subjects (n = 23,981).

	STABILITY		SOLID-TIMI 52	
	Placebo	Darapladib 160mg daily	Placebo	Darapladib 160mg daily
N*	6863	6714	5201	5203
Age (years)	64.3 (9.3)	64.5 (9.2)	64.3 (9.4)	64.0 (9.4)
Male, %	81.2	82.0	74.0	74.8
Current smoking, %	19.0	17.5	18.9	18.9
BMI (kg/m2)	29.0 (4.9)	29.1 (5.09)	28.5 (5.10)	28.6 (5.11)
Waist/hip ratio	0.97 (0.08)	0.97 (0.08)	0.97 (0.09)	0.98 (0.09)
Systolic blood pressure (mmHg)	132.0 (16.4)	132.0 (16.4)	126.0 (16.3)	126.0 (16.2)
Diastolic blood pressure (mmHg)	78.8 (10.2)	78.7 (10.4)	74.6 (10.1)	74.9 (9.9)
Total cholesterol (mg/dL)	4.2 (1.0)	4.2 (1.1)	4.0 (1.1)	4.0 (1.1)
HDL-C (mmol/L)	1.2 (0.3)	1.2 (0.3)	1.1 (0.3)	1.1 (0.3)
LDL-C (mmol/L)	2.2 (0.8)	2.2 (0.9)	2.1 (0.8)	2.1 (0.9)
Triglycerides (mg/dL)	1.8 (1.3)	1.8 (1.4)	1.8 (1.1)	1.8 (1.1)
hsCRP (mg/L)	3.0 (6.6)	2.9 (5.8)	12.4(21.9)	12.6(24.4)
Lp-PLA_2_ activity levels (nmol/min/ml)	176 (47.2)	175 (47.6)	178 (48.2)	178 (49.1)
Statin, %	97.2	97.5	95.2	94.6
Aspirin, %	93.4	92.1	96.4	96.4
Beta blockers, %	79.8	79.2	88.0	87.9
P2Y12 inhibitors, %	33.8	33.5	88.5	88.7
ACE inhibitor or ARB, %	77.7	77.6	83.1	83.2
Chronic kidney disease, %	29.7	30.2	16.7	16.0
Diabetes, %	38.0	38.9	33.5	33.9

Mean (SD) for continuous, percent for categorical variables

*number of subjects that passed genotyping QC

Our PGx analysis included various clinical endpoints and analysis populations, as outlined in Table 2. Further detail on the rationale and definition of the endpoints and analysis populations can be found in the table footnotes and under the Methods section.

**Table 2:** Overview of pharmacogenetic analysis populations by endpoint.

Outcome	Endpoint Variable	Analysis Population	MAF* cut-off	STABILITY (n.event/n.total)	SOLID-TIMI 52 (n.event/n.total)	Meta-Analysis^#^ (n.event/n.total)
Lp-PLA_2_ enzyme activity	Baseline	PGx subjects in placebo arm and darapladib treated arm	> 0%	12640	9516	22156
	Change from baseline	PGx subjects in darapladib treated arm	> 0.5%	6170	4704	10874
Efficacy	MCE	PGx subjects in placebo arm and darapladib treated arm	> 0.5%	Placebo:688/6863; Darapladib:585/6714	Placebo:752/5201; Darapladib:733/5203	Placebo:1440/12064; Darapladib:1318/11917
	MI (fatal + non-fatal)	PGx subjects in placebo arm and darapladib treated arm	> 0.5%	Placebo:358/6863; Darapladib:292/6714	Placebo:475/5201; Darapladib:448/5203	Placebo:833/12064; Darapladib:740/11917
Tolerability	Diarrhea^§^	White PGx subjects with diarrhea event within 90 days of receiving darapladib, excluding subjects on Metformin	> 0.5%	Darapladib:235/3405	Darapladib:246/3155	Darapladib:481/6560
	MS diarrhea^§^ - Moderate and severe	White PGx subjects with diarrhea event within 90 days of receiving darapladib, excluding subjects on Metformin	> 0.5%	Darapladib:97/3267	Darapladib:115/3024	Darapladib:212/6291
	Odor—Bathroom event	PGx subjects in darapladib treated arm	> 0.5%	Darapladib:735/6568	Darapladib:548/5071	Darapladib:1283/11639
	Odor—Non-bathroom event	PGx subjects in darapladib treated arm	> 0.5%	Darapladib:340/6173	Darapladib:244/4769	Darapladib:584/10942

* MAF = Minor Allele Frequency; Analyses included imputed variants (imputation *r*^2^ ≥ 0.5) in both STABILITY and SOLID-TIMI 52 studies. Analysis of Lp-PLA_2_ enzyme activity was performed using 13,572,687 variants, whilst 9,334,585 variants were used for the efficacy and tolerability analyses

^#^ Meta-analyses were done within placebo or darapladib treated arm separately.

^§^ In order to reduce a signal to noise ratio, the diarrhea endpoint was defined as a subset that was most likely associated to treatment with darapladib. For more details, see Material & Methods (Phenotype definition and measurements).

MI = Myocardial infarction; MCE = Major Coronary Event

When we assessed whether the PGx population was representative of the overall clinical study in STABILITY study, a bias was identified in the PGx population resulting in slightly lower hazard ratios in the efficacy endpoint (MCE: PGx subjects HR 0.87 [CI 95% 0.78, 0.97] vs all subjects HR 0.90 [CI 95% 0.82, 1.00]) and the tolerability endpoints (diarrhea: PGx subjects HR 1.9 [CI 95% 1.7, 2.1] vs all subjects HR 2.0 [95% CI 1.8, 2.3]; any odor: PGx subjects HR 5.9 [CI 95% 4.9, 6.9] vs all subjects HR 6.19 [95% CI 5.3, 7.3]). This difference in STABILITY may be explained by the collection of PGx samples at the month 3 study visits, rather than at randomization, thereby missing collection of samples from subjects with early MCE events and those who withdrew from the study due to tolerability issues. PGx sample collection was also delayed in some countries due to late approval of the PGx component to the study. The SOLID-TIMI 52 study, where PGx samples were collected at baseline, did not show this bias.

### Genome-wide association of lipoprotein-associated phospholipase A2 activity at baseline and change from baseline

We first performed a single variant association analysis for Lp-PLA_2_ activity at baseline for imputed variants with imputation *r*^2^ ≥ 0.5 using a linear regression model that included the top 10 principle component (PC) scores as covariates in STABILITY (n = 12,640) and SOLID-TIMI 52 (n = 9,516) separately. Meta-analysis of the ~13.6 million common, low frequency and rare genome-wide variants was carried out to combine the association results from both studies. We detected 578 variants with consistent direction of effect in both STABILITY and SOLID-TIMI 52 studies that surpassed the genome-wide significant P-value threshold of 5E-08 in regions of chromosomes 1, 6 (2 clusters), 9 (2 clusters), 12, 15 and 19. Results for the most significant variant from each chromosomal cluster are summarized in Table 3; with data for other variants in S1 Table. All genomic control (λ_gc_) estimates were close to 1, suggesting no inflation due to uncontrolled population structure (see histograms, QQ and Manhattan plots in S2 Fig).

**Table 3:** Effect estimates, population-specific frequency of top-associated common, low frequency and rare variants contributing to baseline and change from baseline Lp-PLA_2_ enzyme activity.

					Baseline Lp-PLA_2_ activity^§^			Baseline Lp-PLA_2_ activity^§^ adjusting for LDL			Change from baseline Lp-PLA_2_^§^^◆^		Baseline LDL		Baseline HDL		Baseline TRIG		Baseline CRP	
Variant	Gene	Allele	Freq	Imp	Beta	P	Perc*	Beta	P	Perc*	Beta	P	Beta	P	Beta	P	Beta	P	Beta	P
	(Annotation)		(%)#	Rsq#	(SE)			(SE)			(SE)		(SE)		(SE)		(SE)		(SE)	
GWAS																				
rs12740374	CELSR2	T	17.7/	1/	-7.44	3.9E-39	0.77	-5.77	2.8E-28	0.22	-0.46	0.38	-0.16	6.1E-40	0.08	1.5E-10	-0.02	0.13	-0.02	0.441
	(3’UTR)		19.8	1	(0.57)			(0.52)			(0.52)		(0.01)		(0.01)		(0.01)		(0.02)	
rs76863441	PLA2G7	A	1.0/	0.99/	-86.71	1.6E-223	4.08	-85.64	8.2E-301	5.57	-0.24	0.92	0.02	0.81	-0.06	0.45	0.01	0.89	0.08	0.58
	(V279F)		0.5	0.99	(2.72)			(2.31)			(2.33)		(0.07)		(0.07)		(0.07)		(0.14)	
rs10455872	LPA	G	5.9/	0.69/	7.22	4.3E-13	0.24	5.72	7.7E-10	0.09	2.16	0.02	0.12	3.6E-09	0.04	0.09	-0.06	6.8E-03	0.02	0.629
	(intron)		6.9	1	(1)			(0.93)			(0.91)		(0.02)		(0.02)		(0.02)		(0.03)	
rs57578064	TOMM5	A	0.1/	0.94/	32.49	1.4E-08	0.16	29.91	3.2E-08	0.11	-1.42	0.77	0.27	0.03	-0.06	0.63	0.04	0.77	0.25	0.279
	(intron)		0.2	0.93	(5.72)			(5.41)			(4.85)		(0.12)		(0.12)		(0.12)		(0.23)	
rs189889864	intergenic	A	0.02/	0.72/	91.81	8.8E-09	0.16	52.6	2.4E-04	0.07	-2.21	0.89	1.28	1.6E-04	-0.42	0.22	0.37	0.27	-0.3	0.493
			0.02	0.65	(15.96)			(14.33)			(16.21)		(0.34)		(0.34)		(0.34)		(0.43)	
rs11057830	SCARB1	A	15.0/	1/	3.67	7.4E-09	0.15	3.52	1.4E-09	0.11	0.15	0.79	0.03	0.048	-0.01	0.32	0.01	0.55	0.01	0.629
	(intron)		15.2	0.94	(0.63)			(0.58)			(0.57)		(0.01)		(0.01)		(0.01)		(0.02)	
rs2733201	FRMD5	T	7.3/	0.68/	-7.3	5.9E-13	0.23	-6.72	5.2E-13	0.15	0.49	0.60	-0.06	0.004	-0.04	0.08	-0.01	0.62	0	0.987
	(intron)		8.2	0.65	(1.01)			(0.93)			(0.93)		(0.02)		(0.02)		(0.02)		(0.04)	
rs7412	APOE	T	6.1/	1/	-17.11	8.3E-78	1.55	-12.18	1.8E-47	0.36	-0.04	0.96	-0.41	1.3E-96	0.08	1.4E-04	0.23	1.5E-30	0.1	2.4E-03
	(ε2)(R176C)		6.8	0.94	(0.92)			(0.84)			(0.82)		(0.02)		(0.02)		(0.02)		(0.03)	
Candidate genes																				
rs328	LPL	G	9.2/	1/	-3.7	1.5E-06	0.1	-3.3	2.9E-06	0.1	-1.04	0.14	0.02	0.33	0.2	7.6E-32	-0.21	2.3E-35	-0.04	0.154
	(S474X)		9.4	1	(0.77)			(0.7)			(0.71)		(0.02)		(0.02)		(0.02)		(0.03)	
rs6511720	LDLR	T	8.3/	1/	-3.75	1.9E-06	0.11	-2.09	0.004	0.004	0.62	0.39	-0.13	7.4E-15	0.03	0.07	0	0.95	0.03	0.288
	(intron)		9.6	1	(0.79)			(0.72)			(0.71)		(0.02)		(0.02)		(0.02)		(0.03)	

^#^ STABILITY/SOLID-TIMI 52

* % variance explained was reported as the average of % variance explained in STABILITY and SOLID-TIMI 52.

^§^ Lp-PLA_2_ activity (nmol/min/ml)

^◆^ Adjusted for baseline Lp-PLA_2_ activity; see S1 Table for unadjusted P-values.

LDL = Low-density lipoprotein (mmol/L); HDL = High-density lipoprotein(mmol/L); TRIG = Triglycerides(mg/dL); CRP = C-reactive protein(mg/L)

Of the 578 genome-wide significant variants, 25 were contained within the exome component of the genotyping array, while 171 were considered rare variants (minor allele frequency [MAF] <0.5%). The most striking association detected is due to the well-known *PLA2G7* V279F null variant (Table 3) [15;18;25–36]. As expected this variant was mostly observed in Asian countries (Table 4). We also found genome-wide associations (P≤5.0E-08) for three additional rare *PLA2G7* null variants (S2 Table), which tended to be specific to certain ethnic groups (Table 4). Two common *PLA2G7* coding variants also showed genome-wide associations statistically independent of the association between *PLA2G7* V279F variant and baseline Lp-PLA_2_ activity. The V379A common variant was associated with a slightly higher Lp-PLA_2_ activity and the R92H common variant was associated with a slightly lower Lp-PLA_2_ activity (S11 Fig).

**Table 4:** Summary of *PLA2G7* variants observed (allele count and effect size) in STABILITY and SOLID-TIMI 52 studies showing some ethnic group specificity (in bold).

Race (N)	rs76863441 V279F	rs201842579 I317N*	rs1805018 I198T	rs1051931 V379A	rs140020965 Q287X	rs1805017 R92H	rs147252565 T278M*	rs34159425 L389S
All (22147)	339	10	2621	8483	12	12297	3	14
Effect size (All) (Beta, SE)								
Asian—East/Japanese/ South East Asian Heritage (1703)	**298**	**9**	467	423		713		
White -White/Caucasian/European Heritage (18823)	37	1	1796	**7453**	**12**	**10318**	2	1
African American/African Heritage (490)	2		159	221		300		**12**
American Indian or Alaskan Native (244)			23	77		185	1	
Asian—Central/South Asian Heritage (517)	1		112	153		557		
White—Arabic/North African Heritage (195)			30	78		121		1
Mixed race (143)	1		30	67		92		
Native Hawaiian or Other Pacific Islander (14)			1	8		6		

*Variant carrier observed in STABILITY only

Table 3 outlines the most significant variants for each locus associated with baseline and/or change from baseline in Lp-PLA_2_ activity and how this correlates with various baseline lipid and C-reactive-protein levels. The strongest association at the 1p13 locus was for rs12740374, located in the 3’UTR of the *CELSR2* gene. This variant was also significantly associated with baseline LDL and HDL levels. Four significant variants were located in the *LPA* gene. Variant rs10455872 was the most significant and was also associated with baseline LDL levels. Low frequency variant rs57578064 is the most significant in the locus that includes the *TOMM5* and *FBXO10* genes and has not been previously described (S3 Fig). Another novel locus on chromosome 9 contained the intergenic rare variant rs189889864 with a very large effect. A cluster of significant variants were located within the *SCARB1* gene, with the most significant effect observed for rs11057830. Another large cluster of significant variants was observed on chromosome 15, of which intronic variant rs2733201 located in the *FRMD5* gene was the most significant (S3 Fig). The variants in this large cluster are in high LD with each other and fall within several large rearrangements/copy number variations in the region. For the *APOE/APOC1* locus variant rs7412 (APOE ε2 allele) in the *APOE* gene was the most significant variant, which was also associated with baseline LDL and triglyceride levels.

Besides the genome-wide analysis, review of the results for the pre-specified candidate gene variants for Lp-PLA_2_ activity revealed two additional loci that surpassed the candidate gene significance threshold of 5E-04, but were not genome-wide significant (Table 3): *LPL* coding variant rs328, which changes the amino acid at position 474 from a serine to a stop codon, was associated with baseline Lp-PLA_2_ activity, HDL and triglyceride levels (S3 Fig); intronic variant rs6511720 in the *LDLR* gene with baseline Lp-PLA_2_ activity and LDL levels.

### Meta-analysis of change from baseline lipoprotein-associated phospholipase A2 activity

Analysis of change from baseline Lp-PLA_2_ activity after one month of treatment revealed three significant loci around the *CELSR2*, *APOE* and *PLA2G7* genes, none of which were associated with change from baseline Lp-PLA_2_ activity after adjusting for baseline Lp-PLA_2_ activity (Table 3; unadjusted P-values in S1 Table), indicating that the baseline Lp-PLA_2_ genetic association was confounding the change from baseline analysis.

### Meta-analysis of efficacy endpoints

Genome-wide analysis of efficacy focused on the MCE and MI endpoints, looking at potential effects in the darapladib and placebo treated subjects separately. A total of 13,577 STABILITY and 10,195 SOLID-TIMI 52 subjects were analyzed (Table 2) for a total of ~9.3 million common and low frequency variants (MAF >0.5%). Due to the low treatment effect in both the STABILITY and SOLID-TIMI 52 clinical trials there was limited power to identify genetic variants that may impact efficacy (See S1 Fig for power curves). One common locus and six low frequency loci met the genome-wide significant association threshold of 5E-08 with consistent direction of effect in both STABILITY and SOLID-TIMI 52 studies, five of these were associated with MCE and two with MI (Table 5; S5 and S6 Figs). The associated common locus (MAF 24%), rs181937009 which was reasonably well imputed (*r*^2^ STABILITY 0.84 and SOLID 0.75), had a significant allele by treatment interaction effect (P = 6.03E-9) but marginal main genetic effect in individual treatment arms. Carriers of the minor allele in the placebo groups had an elevated HR 1.22 (95% CI 1.11–1.33), meta-P = 4.30E-5 and in the darapladib groups a reduced HR 0.79 (95% CI 0.71–0.88), meta-P = 2.07E-5. All six associated low frequency variants had MAF<2.5% and many had imputation *r*^2^ values between 0.5 and 0.8, which reduces the level of confidence in these results. All genomic control (λ_gc_) estimates were close to 1, suggesting no inflation due to uncontrolled population structure (see QQ and Manhattan plots in S4 Fig).

**Table 5:** List of significant results for efficacy endpoints (MCE and MI).

									Placebo		Darapladib		Combined	
Variant	Gene	Location	Major Allele	Minor Allele	Study	N	MAF	*r*^2^	HR^#^	P	HR	P	P	P*
									(95% CI)		(95% CI)		Interaction	(2 df test)
**Major Coronary Event**														
rs138741635	FHIT	intron	T	A	STABILITY	13577	0.019	0.7	1.89 (1.33, 2.7)	3.36E-04	0.79 (0.46, 1.35)	3.85E-01	6.69E-03	2.41E-03
					SOLID-TIMI 52	10404	0.02	0.66	2.16 (1.52, 3.13)	1.98E-05	0.73 (0.44, 1.2)	2.14E-01	3.89E-04	1.69E-04
					META	23981			2.02 (1.59, 2.56)	**2.87E-08**	0.75 (0.52, 1.09)	1.33E-01	9.01E-06	1.90E-06
rs192427471		intergenic	C	T	STABILITY	13577	0.024	0.67	1.19 (0.79, 1.79)	4.10E-01	2.46 (1.79, 3.45)	4.38E-08	4.03E-03	3.33E-06
					SOLID-TIMI 52	10404	0.021	0.68	0.97 (0.63, 1.49)	8.81E-01	1.61 (1.12, 2.33)	9.57E-03	7.85E-02	5.35E-02
					META	23981			1.08 (0.8, 1.45)	6.23E-01	2.04 (1.61, 2.56)	**6.31E-09**	9.49E-04	1.80E-06
rs181937009		intergenic	A	G	STABILITY	13577	0.244	0.84	1.27 (1.11, 1.45)	5.66E-04	0.78 (0.67, 0.91)	2.14E-03	1.46E-05	6.43E-05
					SOLID-TIMI 52	10404	0.236	0.75	1.18 (1.03, 1.33)	1.95E-02	0.8 (0.69, 0.93)	2.78E-03	9.74E-05	4.42E-04
					META	23981			1.22 (1.11, 1.33)	4.28E-05	0.79 (0.71, 0.88)	2.07E-05	**6.03E-09**	1.00E-07
rs12290663		intergenic	G	A	STABILITY	13577	0.013	0.9	1.03 (0.64, 1.67)	9.18E-01	2.06 (1.37, 3.13)	5.17E-04	1.16E-02	3.08E-03
					SOLID-TIMI 52	10404	0.018	0.65	1.07 (0.68, 1.67)	7.72E-01	2.16 (1.52, 3.03)	1.62E-05	1.30E-02	3.32E-04
					META	23981			1.05 (0.76, 1.45)	7.78E-01	2.11 (1.61, 2.78)	**3.15E-08**	4.07E-04	4.40E-06
rs147204125	ACOT6	upstream	A	G	STABILITY	13577	0.008	0.57	2.31 (1.32, 4)	3.52E-03	1.79 (0.88, 3.57)	1.09E-01	5.49E-01	7.83E-03
					SOLID-TIMI 52	10404	0.011	0.54	3.34 (2.04, 5.56)	1.24E-06	1.71 (1, 2.94)	5.11E-02	9.19E-02	2.40E-05
					META	23981			2.86 (1.96, 4.17)	**2.45E-08**	1.74 (1.14, 2.7)	1.16E-02	9.14E-02	1.70E-06
**Myocardial infarction**														
rs192476688, rs201052613	RP1-15D23.2	intron	AT	A	STABILITY	13577	0.012	0.76	1.23 (0.63, 2.44)	5.62E-01	1.74 (0.88, 3.45)	0.114879	5.54E-01	3.20E-01
					SOLID-TIMI 52	10404	0.011	0.66	0.83 (0.38, 1.82)	6.48E-01	4.29 (2.7, 6.67)	1.58E-09	6.95E-04	3.90E-06
					META	23981			1.04 (0.63, 1.75)	8.94E-01	3.21 (2.17, 4.76)	**4.47E-09**	4.06E-03	1.50E-04
rs117714106		intergenic	C	T	STABILITY	13577	0.007	0.59	0.36 (0.08, 1.69)	1.96E-01	3.75 (1.89, 7.69)	0.000162	5.79E-03	1.88E-03
					SOLID-TIMI 52	10404	0.007	0.61	0.72 (0.23, 2.27)	5.80E-01	3.82 (2.22, 6.67)	8.00E-07	1.02E-02	1.75E-04
					META	23981			0.57 (0.23, 1.43)	2.26E-01	3.79 (2.5, 5.88)	**5.23E-10**	2.09E-04	1.50E-06

# Hazard ratio for minor allele

* A 2-degree-freedom test for both genotype main and genotype by treatment interaction effect

MAF = Minor allele frequency

None of the pre-specified candidate gene variants for efficacy, including the *PLA2G7* V279F null variant, were associated under the candidate gene significance threshold (P = 5E-04) with MCE or MI in either the darapladib or placebo treatment arm. Also none of the variants that were associated with Lp-PLA_2_ activity were associated with efficacy endpoints (MCE or MI events)(S8 Fig).

### Meta-analysis of tolerability endpoints

To investigate genetic variants that could impact darapladib tolerability, we performed genome-wide analysis of ~9.3 million common variants for the diarrhea and odor-related endpoints, evaluating darapladib treated subjects only (Table 2). In order to reduce a signal to noise ratio, the diarrhea endpoint was defined as a subset that was most likely associated to treatment with darapladib (limited to subjects with diarrhea experienced in the first 90 days of darapladib treatment, excluding subjects who were also treated with metformin [diarrhea is a well-known side-effect of metformin treatment] and subjects of White race only. Three loci on chromosome 6, 16 and X (males only) respectively were significantly associated with the diarrhea endpoint at the genome-wide level (S5 Table). A further eight loci met the genome-wide significant association threshold for the more stringent moderate/severe (MS) diarrhea endpoint (S5 Table). Most of the significant variants at all 12 associated loci had low allele frequencies, ranging from 0.6 to 1.5%, with few alleles observed in the population analyzed. Only the loci on chromosomes 5, 9 and X (females) included significant variants with frequencies around 3.5–4.5%. There was no correlation between the diarrhea endpoints and Lp-PLA_2_ activity levels in the darapladib treatment arm at these loci (S8 Fig).

Analysis of the bathroom and non-bathroom related odor endpoints did not identify any significant genome-wide associations, although we did observe some regional differences in reporting of the odor events (see S1 Text for details).

## Discussion

We have performed a comprehensive PGx analysis of pharmacodynamic, efficacy and tolerability phenotypes in two large darapladib cardiovascular outcomes trials which failed to meet their primary endpoints. Although the trials did not show efficacy of darapladib, understanding whether genetics could delineate responsive subgroups was considered a key question. Additionally, it was important to understand the tolerability phenotypes as an alternative indication for darapladib was under consideration.

In addition to the V279F null allele we identified three other rare null *PLA2G7* alleles within various ethnic groups, but did not observe any association with clinical endpoints. We replicated the associations of six published loci on baseline Lp-PLA_2_ activity levels and identified three novel loci. The analysis of darapladib tolerability endpoints did not reveal any robust, high confidence genetic associations, since most of the significant variants at the associated loci had low allele frequencies, with few alleles observed and analyzed. Efficacy analyses revealed only one common variant which had a differential effect by treatment arm, conferring risk in the placebo arm but reducing risk in the darapladib arm. Furthermore, none of the loci associated with Lp-PLA_2_ activity levels at baseline were associated with any of the drug response endpoints.

### Lipoprotein-associated phospholipase A2 activity

In the present meta-analysis, we identified eight loci associated with baseline Lp-PLA_2_ activity. Of these eight loci, five had been previously described in other GWAS studies of Lp-PLA_2_ activity [21–23]. *PLA2G7* null variant rs76863441 (V279F) was the most significant variant associated with baseline and change from baseline Lp-PLA_2_ activity. Although this variant is almost exclusively observed in East-Asian populations, other common *PLA2G7* variants were also significant in this study and mirror observations from the Framingham Heart, CHARGE and JUPITER studies [21–23]. Other genome-wide significant loci that have previously been reported include *CELSR2*, *SCARB1*, *LPA* and *APOE* [21–23]. Whilst four genes are markedly linked to LDL-cholesterol (*CELSR2*, *LPA*, *APOE* and *LDLR*) and likely represent the known positive correlation between Lp-PLA_2_ and LDL-cholesterol, two HDL-cholesterol associated genes (*LPL* and *SCARB1*) were also associated with Lp-PLA2. This is not surprising as HDL particles are known to carry a minority of Lp-PLA_2_.

Additionally, three novel loci were found, one of which is represented by an intronic *FRMD5* gene variant on chromosome 15 in a region of large copy number variation, some of which encompass the entire gene. Genome-wide significant associations have been reported between *FRMD5* variants and triglyceride levels [37], but not with Lp-PLA_2_ activity. The other two loci were observed on chromosome 9, both rare variants with large effects. Carriage of the A allele of a *TOMM5* intronic variant was correlated with a large increase in Lp-PLA_2_ activity and observed at very low frequency (0.15%). Database review to assess possible impact on gene regulation did not reveal any convincing evidence. It is also located upstream of the *FBXO10* gene, for which sub-genomewide associations have been reported with lipid traits and triglyceride levels (dbGAP data entries pha003081 & pha003082 from the NHLBI Family Heart Study study) and lymphocyte counts [38]. For the second locus, only seven A alleles were observed for the top-associated variant rs189889864 in the 22,150 subjects analyzed. This locus falls in a region with many olfactory receptor family 13 genes and multiple sub-genomewide associations have been reported between the *OR13C5* and *OR13C9* genes and lipid traits, triglyceride levels, stroke and coronary artery calcification [39–42] (dbGAP data entry pha002887 for STAMPEED Cardiovascular Health Study). On the basis of these results it appears that only the *PLA2G7* gene itself is completely independent of lipids. None of these eight loci were associated with change from baseline Lp-PLA_2_ activity after adjusting for baseline Lp-PLA_2_ activity, nor were they linked to efficacy in terms of the MCE and MI endpoints.

A composite of the genetic variants associated with baseline Lp-PLA_2_ activity may provide a useful tool in future studies where the impact of genetics on Lp-PLA_2_ activity is assessed. The variance explained in Lp-PLA_2_ activity by all significant associated variants reported in Table 3 and additional *PLA2G7* null variants was 8.34% in all subjects, 38.63% in East Asians and 4.89% in Whites, versus 4.08% in all, 35.45% in East Asians and 0.53% in Whites by the well known *PLA2G7* V279F null variant itself. These data suggest that the multi-variant predictor can be used as a Mendelian randomization “instrument” in human disease genetics analyses, particularly in European populations where common variants have very small effects alone. However, the correlation with lipids would need to be accounted for appropriately in any evaluation of the score with a disease endpoint.

We also identified additional significant rare null alleles within the *PLA2G7* gene which were population specific; I317N in East Asians, Q287X in whites and L389S in subjects of African origin. Based on their effect on Lp-PLA_2_ activity, T278M and R82H (S2 Table) are also likely to be null alleles, but were too infrequent to generate significant results. Carriers of null alleles did not demonstrate any difference in either MCE or MI as compared to non-carriers.

### Darapladib efficacy & tolerability

The genome-wide meta-analysis did not reveal any high confidence effects of common genetic variants on darapladib tolerability, even though the study was relatively well powered to detect these effects. Our ability to identify efficacy variants though was dramatically reduced by the low treatment effects observed in STABILITY and no treatment effect observed in the SOLID-TIMI 52 clinical trials, leading to low power for the PGx analysis. Nevertheless, six low frequency (MAF<2.5%) loci associated with the MCE or MI endpoints suggested a marginally improved outcome with the common allele following treatment with darapladib when compared to placebo (see S1 Text for more details). However, many of these imputed variants had *r*^2^ values between 0.5 and 0.8, leading to some uncertainty as to their validity. One common variant located 3.2kb 5' of RP3-332B22.1 had a significant interaction P-value, but was only marginally significant in the placebo and darapladib arms. Nevertheless, approximately 24% of subjects carried the minor allele of rs181937009 which was associated nominally with 22% elevated risk in the placebo patients but a 21% reduction in risk in the darapladib arms. The results were consistent in the two trials and were reasonably well imputed, suggesting this region may warrant further investigation. Despite the significant association in the placebo arm, this is not a known cardiovascular risk locus and did not demonstrate a nominally significant effect in the CARDIoGRAMplusC4D meta-analysis of coronary artery disease with ~185,000 cases (P = 0.063)[43]. Assessment of the genomic control parameter by MAF or imputation *r*^2^ bins did not identify any significant deviation from 1 for lower frequent (MAF<1%) or less well imputed variants (*r*^2^<0.8). Ideally, these variants should be directly genotyped to confirm, but that is not feasible at the present time.

Analysis of darapladib tolerability endpoints was challenging due to uncertainty around the validity of the more heterogeneous and subjective nature of the diarrhea and odor endpoints, as well as the high prevalence of diarrhea in the placebo arm for both trials. In order to reduce the non-specificity of the diarrhea phenotype and to focus on diarrhea related to darapladib drug treatment, we limited the time window and ethnicity (reduce heterogeneity) as well as excluded metformin users. Initial meta-analysis was performed using a >0.1% minor allele frequency threshold. However, as this generated a lot of noise in the results and complicated the interpretation of the data the minor allele frequency threshold was raised to 0.5% to alleviate these problems. Most of the loci associated with diarrhea were represented by variants with frequencies ranging from 0.6 to 1.5% and no association was seen with common variants (MAF>5%) in any of the tolerability meta-analyses (see S1 Text for more details). Although these observations with low frequency variants are interesting, the impact of these on any clinical utility is limited.

The lack of association signals for the odor-related endpoints is likely due to the heterogeneity and subjectivity of this endpoint, highlighted by the regional and possibly cultural differences in reporting (S6 Table). S10 Fig, a principle components world map for STABILITY subjects, highlights the great diversity in country origin which is inherent to large clinical trials.

### Strengths and limitations

A key strength of this study was the genotyping conducted while both trials were ongoing, allowing opportunity to timely identify genes contributing to drug response at the time of trial completion had they existed. Additionally, by combining both trials a total number of ~24,000 subjects available for the analysis of various endpoints. However, the lack of treatment effect in the STABILITY and SOLID-TIMI 52 clinical trials severely limited the power to find any efficacy genetic effects. This mirrors a recent review of efficacy PGx studies [44] which concluded that drugs that have failed in their primary efficacy objectives have a minimal chance of being rescued by a pharmacogenetics approach. Even though there was no efficacy observed in these large Phase III clinical studies, few large PGx study based on cardiovascular outcomes had been performed previously and therefore exploring a dataset like this could still reveal novel findings. DNA collection at month 3 for STABILITY introduced a bias which influenced the genetics analysis and illustrates why it is important to collect samples at baseline.

Additionally, the different disease background of the STABILITY (chronic heart disease) and SOLID-TIMI 52 (acute coronary syndrome) clinical trials may have had an impact on our ability to see a consistent genetic effect on response to darapladib treatment. Cardiovascular disease trials are extremely heterogeneous with broad endpoints (MACE), which are challenging for genetic studies. It is worth noting that genetic studies for CHD used to predict drug effects tend to have much more discrete set of phenotypes (such as MI or angiographically defined disease) as compared to the phenotypes in the STABILITY and SOLID-TIMI 52 trials. For the darapladib trials combining stroke and CHD meant a limited genetic overlap in these broad endpoints. Besides the analysis of baseline Lp-PLA_2_ activity, none of the efficacy or tolerability endpoint analyses included rare variants (<0.5% allele frequency) and therefore any genetic signals from rare variants will not have been detected in this study.

For GWAS, the classical GWAS-significance threshold (P≤5E-08) was applied to declare significant results. This threshold was established at a time when most GWAS involved approximately 1 million independent tests, mostly for common variants with MAF>5%. Given the larger number of both common and low frequency variants (MAF<5%) to be analyzed here, this implies a lack of stringent multiple testing correction such that any associations reaching classical genome wide significance would be in need of further follow-up for validation or replication.

## Conclusions

Meta-analysis of two large darapladib Phase III trials identified only one region with common variants that significantly influenced differential response to darapladib, though the main effects were not statistically significant. No major loci for tolerability were found. Ten variants associated with baseline Lp-PLA_2_ activity were observed, which, further demonstrated the close relationship between Lp-PLA_2_ activity and lipoproteins and extends the number of variants that can be included in a gene score for Lp-PLA_2_, for investigation of other diseases or conditions. These genetic variants associated with baseline Lp-PLA_2_ activity levels did not however contribute to the interpretation of darapladib treatment response, which seems to correspond with the lack of effect of Lp-PLA2 inhibition on cardiovascular events in the trials. Thus, genetic analysis confirmed and extended the influence of lipoprotein loci on Lp-PLA2 levels, identified some novel null alleles in the PLA2G7 gene, but did not identify any major efficacious subgroups within these two large clinical trials.

Significant exome variants, included as additional content on the genotyping array, were useful for the baseline and change from baseline Lp-PLA_2_ activity endpoints, as this content also allowed the identification of additional *PLA2G7* null allele carriers. Finally, this darapladib meta-analysis represents one of the largest PGx studies ever conducted, providing a large pool of data with comprehensive data mining opportunities, to further the understanding of the mechanism of action of Lp-PLA_2_ inhibitors and CHD disease genetics.

## Materials and methods

### Study population

Subjects included in our study were recruited as part of two darapladib Phase III trials: STABILITY (STabilization of Atherosclerotic plaque By Initiation of darapLadIb TherapY) and SOLID-TIMI 52 (The Stabilization Of pLaques usIng Darapladib-Thrombolysis In Myocardial Infarction 52). Clinical study approval was provided by the relevant Ethics Committee of each participating country: Argentina, Australia, Belgium, Brazil, Bulgaria, Canada, Chile, Colombia, Czech Republic, Denmark, Estonia, France, Germany, Greece, Hong Kong, Hungary, India, Israel, Italy, Japan, Korea, Mexico, Netherlands, New Zealand, Norway, Pakistan, Peru, Philippines, Poland, Romania, Russia, Slovakia, South Africa, Spain, Sweden, Taiwan, Thailand, Turkey, Ukraine, United Kingdom, United States. A full list of Ethics Committees and Independent Review Boards used in the STABILITY and SOLID-TIMI 52 trials can be found in S7 and S8 Tables respectively. Clinical investigation was conducted according to the principles expressed in the Declaration of Helsinki. Blood samples for genetic analysis were obtained at or after randomization once subjects had provided written informed consent for genetic analysis.

All STABILITY subjects had chronic CHD, defined as prior MI, prior coronary revascularization or multivessel CHD without revascularization. In addition, subjects had to fulfill at least one of the following: age ≥60 years; diabetes mellitus requiring pharmacotherapy; HDL-cholesterol <1.03 mmol/l; current or previous (discontinuation within three months of randomization) smoker defined as ≥ 5 cigarettes per day on average; significant renal dysfunction (estimated glomerular filtration rate ≥30 and <60 mL/min per 1.73 m2 or urine albumin:creatinine ratio ≥30 mg albumin/g creatinine); or polyvascular disease (CHD and cerebrovascular disease or CHD and peripheral arterial disease)[11]. Among the 15,828 Intent-to-Treat recruited subjects, 13673 (86%) provided informed consent for genetic analysis. The self-reported racial distribution of these subjects was 80.2% White, 2% African-American/Black, 15.4% Asian and 2.6% other.

All SOLID-TIMI 52 subjects were recruited based on having experienced acute coronary syndrome (ACS; unstable angina, non–ST-elevation or ST-elevation MI) within 30 days of randomization. In addition, subjects had to fulfill at least one of the following: age ≥60 years; history of MI prior to the qualifying event, significant renal dysfunction (estimated glomerular filtration rate ≥30 and <60 mL/min per 1.73 m2), diabetes mellitus requiring pharmacotherapy, or polyvascular disease (cerebrovascular disease [carotid artery disease or prior ischemic stroke] or peripheral arterial disease)[12]. Among the13,026 Intent-to-Treat recruited subjects, 10,621 (82%) provided informed consent for genetic analysis. The self-reported racial distribution of these subjects was 88.7% White, 2.5% African-American/Black, 5.6% Asian and 3.2% other.

The above study recruitment criteria highlight a considerable difference between the chronic disease phenotype for the STABILITY study and the acute phenotype in the SOLID-TIMI 52 study. See Table 1 for the characteristics of the STABILITY and SOLID-TIMI 52 study populations. Tolerability phenotypes had similar characteristics across the two trials (Table 2).

### Phenotype definition and measurements

Baseline values were defined as the last value obtained until and including the date of randomization. Plasma Lp-PLA_2_ enzyme activity was measured by diaDexus, Inc. (South San Francisco, CA) using the PLAC® Test for Lp-PLA_2_ Activity, both at baseline and post-baseline at the Month 1 treatment visit. At Month 1 most of the effect of Lp-PLA_2_ inhibition by darapladib was expected to have occurred plus the impact of subject withdrawal was minimal, therefore the maximum number of subjects would be available for evaluation.

LDL, HDL, hsCRP levels were measured by Quest (Quest Diagnostics Clinical Trials Laboratory, Valencia, California, USA or affiliated laboratories).

Efficacy analyses were focused on the Major Coronary Events (MCE) and myocardial infarction (MI) endpoints in both the darapladib and placebo treatment arms. The pharmacodynamic analysis of Lp-PLA_2_ activity was performed for baseline and change from baseline (without adjustment for baseline and adjusted for baseline as sensitivity analysis) in the darapladib treatment arm after one month of treatment.

To evaluate darapladib tolerability, both diarrhea and odor-related endpoints were analyzed in the darapladib treatment arm only. In order to reduce a signal to noise ratio, the diarrhea endpoint was defined as a subset that was most likely associated to treatment with darapladib. Subjects were included based on a) diarrhea experienced in the first 90 days of darapladib treatment (time of onset of diarrhea in placebo arm tended to be later); b) excluding subjects who were also treated with metformin (since diarrhea is a well-known side-effect of metformin treatment); c) including subjects of White race only (reduce cultural reporting bias). Additionally, a stricter diarrhea phenotype was also evaluated, focusing on subjects who experienced moderate and/or severe diarrhea (MS Diarrhea; S9 Fig). For the odor-related phenotype, Bathroom (abnormal urine or faeces odor) and Non-Bathroom (mainly skin) related endpoints were analyzed as the main odor endpoints.

S1 Fig shows the power curves for the ability of this study to detect variants associated with the pharmacodynamic, efficacy and tolerability endpoints.

### Genotype data and quality control

STABILITY: Genotype data for 951,117 markers were generated on the HumanOmniExpressExome-8 v1 array (Illumina Inc., San Diego, CA, USA) by Expression Analysis Inc. (Durham, North Carolina, USA) for 13,728 samples. Quality control was performed using the toolset PLINK v1.07 [45] and SNP & Variation Suite v7.7 (Golden Helix, Bozeman, MT, USA; www.goldenhelix.com). Individuals were excluded if genotyping call rates were less than 95%, if they had a 3rd degree or higher relationship as determined by kinship coefficient estimates using KING software [46] or, if the GWAS genotype predicted gender did not match the annotated gender. Markers were excluded if they had call rates less than 95%, were monomorphic or if Hardy-Weinberg p-values were less than 10^−7^ in PCA defined White and Korean subgroups. PCA analysis was carried out using the SNPRelate [47] software for variants with MAF > 5% and pairwise *r*^2^ < 0.2. When a variant had multiple markers on the array the lowest efficiency marker was removed. For markers with minor allele frequency (MAF) less than 0.5% missing genotypes were recalled using zCall [48]. After quality control, the resulting dataset included 13,577 individuals genotyped on 881,788 variants. For the purpose of this study, we defined rare variants as MAF<0.5%, low frequency as 0.5≤MAF<5% and common variants as MAF≥5%.

Additional STABILITY genotype data was generated by LGC Genomics for 12 candidate variants in 13,752 samples using KASP technology (LGC Genomics, Hoddesdon, UK). No markers were excluded and subjects were excluded if they had missing data for >3 markers.

SOLID-TIMI 52: Genotype data for 692,259 markers were generated on the Axiom® Biobank Plus Genotyping Array with custom content array (Affymetrix, Santa Clara, CA, USA) by BioProcessing Solutions (BPS, Piscataway, NJ, USA) for 10,619 samples. Quality control was performed using the toolset PLINK v1.07 [45]. Individuals were excluded if genotyping call rates were less than 97%, if they had a 3rd degree or higher relationship as determined by kinship coefficient estimates using KING software [46] or if the GWAS genotype predicted gender did not match the annotated gender. Markers were excluded if they had call rates less than 95%, were monomorphic or if Hardy-Weinberg p-values were less than 10^−6^ in White and Korean subgroups. When a variant had multiple markers on the array the best assay was included using the Affymetrix SNPolisher Best Probeset filter (http://www.affymetrix.com/estore/partners_programs/programs/developer/tools/devnettools.affx).

Additional SOLID-TIMI 52 genotype data was generated by BPS for 35 candidate variants in 10,054 samples using Fluidigm® Biomark^TM^ technology (Fluidigm Inc., South San Francisco, CA, USA). No markers were excluded and subjects were excluded if they had missing data for >3 markers.

For the efficacy and pharmacodynamic (Lp-PLA_2_ activity levels) analyses candidate genes or loci and key variants were selected based on published association data for CVD risk and Lp-PLA_2_ activity and/or mass and related pathways.

### 1000 Genome imputation

1000 Genome imputation was performed using the 1000 Genomes Project Reference Panel (phase I, 2012-03-14 haplotypes, genome map GRCh37)(http://www.1000genomes.org/), which contains haplotypes on 1092 samples for ~39.7 million bi-allelic polymorphic markers, by University of Michigan for the STABILITY study and by GSK for the SOLID-TIMI 52 study. Prior to genotype imputation, additional GWAS variants were excluded if Hardy-Weinberg p-values were less than 10^−7^ in the Principle Component Analysis (PCA)-defined White population or if they showed irreconcilable differences in alleles or allele frequency with reference panel genotypes. Pre-phasing of STABILITY GWAS samples was performed per chromosome using SHAPEIT v2.644 [49] with the options of 200 states and 2.5 Mb window. Imputation was carried out in 1,109 chunks with chunk size 2.5 Mb plus 500 kb flanking region using minimac 2013.7.17 [50,51] for STABILITY. For SOLID-TIMI 52 samples HAPI-UR was used [52]. After imputation, QC metrics were examined to identify strand flip errors (e.g. correlation between measured and imputed genotype close to *r*^2^ = -1 plus a comparison of the reference allele frequency data with the imputation allele frequency data). For X chromosome, male and female subjects were phased and imputed separately using the same methods.

In summary, data for a total of approximately 882,000 (STABILITY) and 547,000 (SOLID-TIMI 52) common and low frequency coding variations using GWAS arrays enriched for exome variants were used for imputation of genome-wide variants that were not part of the GWAS arrays, resulting in approximately 13.6M markers post-QC. Imputation data for STABILITY and SOLID-TIMI 52 were kept separately for the statistical analysis.

### Statistical analysis

#### Summary of study population

Characteristics of the study population were summarized by number of sample size, mean and standard deviation for continuous variables related to demographics and laboratory measures, percentage for binary variables related to demographics, the use of co-medication and confounding diseases.

#### Power evaluation

Basic power calculations were conducted to inform the range of genetic effects that could be found with various genetic effects and allele frequencies for all endpoints considered. For baseline and post-baseline data for Lp-PLA_2_ enzyme activity (S1A Fig), plotted on the x-axis, allelic mean difference is the standardized effect for each copy of the effect allele. For example, while baseline Lp-PLA_2_ data was analyzed using samples from both placebo and darapladib arms, assuming an allelic mean difference = 0.15 and a sample size of 23,000, the power to detect a genetic association given an effect allele frequency of 5% is approximately 94% for alpha = 5E-08. As change from baseline Lp-PLA_2_ was evaluated in the darapladib arm only, assuming an allelic mean difference = 0.15 and a sample size of 11,500, the power to detect a genetic association given an effect allele frequency of 5% is approximately 31% for alpha = 5E-08. To detect genetic variants associated with efficacy endpoints related to darapladib treatment, a sample size of 23,000 is assumed, based on the total number of PGx subjects from both STABILITY and SOLID trials for analysis. Given an event rate of 4% per year and a median follow-up of 3 years, and risk reductions of 10% and 0% in STABILITY and SOLID, respectively, assuming subjects carrying the genetic allele respond better to the treatment than the non-carriers and the treated non-carriers having an event rate less than or similar to those in placebo arm, S1B Fig shows the power to detect a genetic association when the genetic effect exists in the treated arm but not in the placebo arm. The optimal power exists for variants with a risk allele frequency around 5%. For example, assuming a hazard ratio of 0.6 for each copy of the effect allele for a genetic variant, the power to detect a genetic association given the risk allele frequency at 5% is approximately 44% and 98% for alpha = 5E-08 and 0.001, respectively. However, power for other risk allele frequencies is either limited due to rare frequencies or not applicable because it is impossible for a variant with an allele frequency ≥ 10% to have a 20% or greater risk reduction in carriers comparing to non-carriers, due to the constraint on the proportion of allele carriers and the size of overall treatment effect. To detect genetic variants associated with tolerability endpoints related to darapladib treatment, a sample size of 11,500 is assumed, based on the total number of PGx subjects in the darapladib treated arm from both STABILITY and SOLID trials for analysis. For example, assuming a hazard ratio of 2 for each copy of the effect allele for a genetic variant, the power to detect a genetic association given the risk allele frequency at 5% is approximately 100% (S1C Fig) for alpha = 5E-08.

#### Analyses of Lp-PLA_2_ enzyme activity

A linear regression model was used to assess the association of allelic dosage with continuous traits including Lp-PLA_**2**_ activity at baseline for all subjects and change from baseline at month 1 for darapladib treated subjects. Both endpoints showed symmetric bell shaped distribution and were winsorized by limiting the extreme outliers to ±4xSD boundary before analysis. To accommodate the uncertainty of imputed genotypes, allelic dosage, i.e. count of non-reference alleles weighted by imputed genotype probabilities, was used assuming an additive genetic model. The top 10 PCs were included as covariates to account for population structure. Baseline LDL was included as an additional covariate for variants showing significant association with baseline Lp-PLA_**2**_ activity. Analyses were conducted in STABILITY and SOLID-TIMI 52 separately. For baseline and change from baseline Lp-PLA_**2**_ activity data, summary statistics from both trials were meta-analyzed using the inverse variance method using METAL [53]. For baseline common, low frequency and rare variants were analyzed, whilst only low frequency and common variants (MAF>0.5%) were analyzed for change from baseline Lp-PLA_**2**_ activity. This is because we were specifically interested in finding and characterizing additional null alleles, which were likely to be rare.

#### Evaluation of association with efficacy outcomes

A Cox proportional hazards regression model was used to assess the association of genotype of common variants (MAF>0.5%) with time to event outcome for the efficacy endpoints (MCE and MI). A multivariate Cox model that includes treatment, genotype and treatment by genotype interaction and top 10 PCs was used to detect genetic variants associated with efficacy response. A 2-degree-freedom test for both genotype main and genotype by treatment interaction effect was performed. P value, effect size estimates (hazard ratio) and standard errors (SEs) for genotype main effect in treated and placebo arm were obtained in STABILITY and SOLID-TIMI 52 separately. Summary statistics from both trials were meta-analyzed using the inverse variance method using METAL [53] in each arm separately. For results meeting the significance threshold, Kaplan-Meier plots were generated to evaluate if the proportional hazards assumption is valid (parallel curves by genotypes).

#### Evaluation of association with tolerability endpoints

A Cox proportional hazards regression model was used to assess the association of genotype of common variants (MAF>0.5%) with time-to-event outcome for tolerability endpoints (Diarrhea and Odor) in patients treated by darapladib, including allelic dosage assuming an additive genetic model and top 10 PCs as covariates to account for population structure. One degree-freedom P value, effect size estimates (hazard ratio) and SEs for genotype main effect in the darapladib treatment arm were calculated in STABILITY and SOLID-TIMI 52 separately. Summary statistics for the darapladib treatment arm from both trials were meta-analyzed using the inverse variance method using METAL [53].

#### Analysis of the X-chromosome

The X chromosome was imputed and analyzed by sex for each trait in STABILITY and SOLID-TIMI 52 separately. As more than 70% of patients were males in both studies, meta-analysis of all endpoints were conducted in males based on summary statistics from both trials using the inverse variance method in METAL [53].

#### Multiple testing adjustment

The Bonferroni corrected multiple test threshold for declaring statistical significance was calculated as P≤5.0E-04 for the candidate gene analysis (0.05/ number of variants tested). For GWAS, the classical GWAS-significance threshold (P≤5E-08) was applied to declare significant results. This threshold was established at a time when most GWAS involved approximately 1 million independent tests, mostly for common variants with MAF>5%. Given the larger number of both common and low frequency variants (MAF<5%) to be analyzed here, this implies a lack of stringent multiple testing correction such that any associations reaching classical genome wide significance would be in need of further follow-up for validation or replication. Due to the exploratory nature of the genetic analyses, no further correction was applied to adjust for the number of endpoints tested.

## Supporting information

S1 Text Supporting text. Details on differences in reporting of odor events and functional insights for variants associated with efficacy and tolerability endpoints.
(DOCX)

S1 Fig Power plots. Power for a) Baseline Lp-PLA_2_ activity in both placebo and darapladib arms (n = 23,000) and Change from Baseline in darapladib arm (n = 11,500) at alpha = 5E-08; b) efficacy endpoints in both placebo and darapladib arms (n = 23,000) at alpha = 0.001 (candidate gene) and 5E-08 (GWAS) tolerability endpoints in darapladib arm only arms (n = 11,500) at alpha = 0.001 (candidate gene) and 5E-08 (GWAS).
(PDF)

S2 Fig Manhattan, QQ, and histogram for baseline Lp-PLA_2_ activity. (PDF)

S3 Fig Regional, forest and box plots of novel loci associated with baseline Lp-PLA_2_ activity. Regional, forest and box plots of mean difference (MD) of novel loci (with greater than 10 copies of variant alleles) associated with baseline Lp-PLA_2_ activity at genome-wide (a&b) and candidate gene (c) significance. The dashed line in the region plots indicates the significance threshold.
(PDF)

S4 Fig Manhattan, QQ and histogram plots for efficacy endpoints. For MCE: a) main genotype effect P-value in placebo arm, b) main genotype effect P-value in darapladib, c) genotype by treatment interaction P-value, and d) 2df test P-value for both main genotype and genotype by treatment interaction; For MI: e) main genotype effect P-value in placebo arm, f) main genotype effect P-value in darapladib, g) genotype by treatment interaction P-value, and h) 2df test P-value for both main genotype and genotype by treatment interaction.
(PDF)

S5 Fig Regional, survival and forest plots of variants associated with MCE following placebo and darapladib treatment. The dashed line in the regional plots indicates the significance threshold. a) rs138741635, b) rs192427471, c) rs181937009, d) rs12290663, e) rs147204125.
(PDF)

S6 Fig Regional, survival and forest plots of variants associated with MI following placebo and darapladib treatment. The dashed line in the regional plots indicates the significance threshold. a) rs192476688, rs201052613, b) rs117714106.
(PDF)

S7 Fig Manhattan, QQ and histogram plots for tolerability endpoints. a) diarrhea in darapladib arm, b) moderate and severe diarrhea in darapladib arm, c) bathroom relate odor events in darapladib arm, d) non-bathroom relate odor events in darapladib arm.
(PDF)

S8 Fig Heat map of P-values observed for the genome-wide significant variants for Lp-PLA_2_ enzyme activity, efficacy and tolerability endpoints. (PDF)

S9 Fig Analysis population for diarrhea using STABILITY as an example. (PDF)

S10 Fig World map constructed using principle components from GWAS data of STABILITY subjects. Highlights the great diversity in country origin which is inherent to large clinical trials.
(PDF)

S11 Fig Baseline Lp-PLA_2_ activity level plots for low frequency and rare variants in the *PLA2G7* gene conditioned on V279F. Main plot shows association P-values (main plot), with effect estimates in top right insert.
(PDF)

S1 Table List of significant results for baseline and change from baseline Lp-PLA_2_ activity. *r*^2^: imputation quality.
(XLSX)

S2 Table Association results between *PLA2G7* functional variants and baseline Lp-PLA_2_ activity. (XLSX)

S3 Table List of candidate gene variants. (XLSX)

S4 Table Candidate gene variant results for efficacy endpoint. *r*^2^: imputation quality; EFF: effect size for log hazard ratio for minor allele; trt: darapladib arm; plc: placebo arm; SE = standard error; Direction: effect direction in STABILITY and SOLID-TIMI 52 trials respectively.
(XLSX)

S5 Table List of significant results for tolerability endpoints (diarrhea and moderate/severe diarrhea events). Meta-analysis P-values are indicated in bold.
(XLSX)

S6 Table Proportion of reported odor events by region. (XLSX)

S7 Table List of names and addresses of IEC/IRB Committees used for the STABILITY trial. (XLSX)

S8 Table List of names and addresses of IEC/IRB Committees used for the SOLID-TIMI 52 trial. (XLSX)

## References

[1] Zalewski A, Macphee C, Nelson JJ. Lipoprotein-associated phospholipase A2: a potential therapeutic target for atherosclerosis. Curr Drug Targets Cardiovasc Haematol Disord 2005;56:527–32. 10.2174/156800605774962103 16503872

[2] Garza CA, Montori VM, McConnell JP, Somers VK, Kullo IJ, Lopez-Jimenez F. Association between lipoprotein-associated phospholipase A2 and cardiovascular disease: A systematic review. Mayo Clin Proc 2007;822:159–65. 10.4065/82.2.159 17290721

[3] Zalewski A, Macphee C. Role of lipoprotein-associated phospholipase A2 in atherosclerosis: biology, epidemiology, and possible therapeutic target. Arterioscler Thromb Vasc Biol 2005 5;255:923–31. 10.1161/01.ATV.0000160551.21962.a7 15731492

[4] Häkkinen T, Luoma JS, Hiltunen MO, Macphee CH, Milliner KJ, Patel L, et al Lipoprotein-associated phospholipase A2, platelet-activating factor acetylhydrolase, is expressed by macrophages in human and rabbit atherosclerotic lesions. Arterioscler Thromb Vasc Biol 1999;1912:2909–17. 10.1161/01.atv.19.12.2909 10591668

[5] Kolodgie FD, Burke AP, Skorija KS, Ladich E, Kutys R, Makuria AT, et al Lipoprotein-associated phospholipase A2 protein expression in the natural progression of human coronary atherosclerosis. Arterioscler Thromb Vasc Biol 2006;2611:2523–9. 10.1161/01.ATV.0000244681.72738.bc 16960105

[6] Serruys PW, Garcia-Garcia HMM, Buszman P, Erne P, Verheye S, Aschermann M, et al Effects of the Direct Lipoprotein-Associated Phospholipase A_2_ Inhibitor Darapladib on Human Coronary Atherosclerotic Plaque. Circulation 2008;11811:1172. 10.1161/CIRCULATIONAHA.108.771899 18765397

[7] Wilensky RL, Shi Y, Mohler ER, Hamamdzic D, Burgert ME, Li J, et al Inhibition of lipoprotein-associated phospholipase A2 reduces complex coronary atherosclerotic plaque development. Nat Med 2008;1410:1059–66. 10.1038/nm.1870 PMC2885134 18806801

[8] Macphee C, Benson GM, Shi Y, Zalewski A. Lipoprotein-associated phospholipase A2: a novel marker of cardiovascular risk and potential therapeutic target. Expert Opin Investig Drugs 2005;146:671–9. 10.1517/13543784.14.6.671 16004595

[9] Macphee CH, Nelson JJ, Zalewski A. Lipoprotein-associated phospholipase A2 as a target of therapy. Curr Opin Lipidol 2005;164:442–6. 10.1097/01.mol.0000174155.61307.5f 15990594

[10] Zalewski A, Nelson JJ, Hegg L, Macphee C. Lp-PLA2: a new kid on the block. Clin Chem 2006;529:1645–50. 10.1373/clinchem.2006.070672 16873290

[11] White H, Held C, Stewart R, Watson D, Harrington R, Budaj A, et al Study design and rationale for the clinical outcomes of the STABILITY Trial STabilization of Atherosclerotic plaque By Initiation of darapLadIb TherapY. comparing darapladib versus placebo in patients with coronary heart disease. Am Heart J 2010;1604:655–61. 10.1016/j.ahj.2010.07.006 20934559

[12] O'Donoghue ML, Braunwald E, White HD, Serruys P, Steg PG, Hochman J, et al Study design and rationale for the Stabilization of pLaques usIng Darapladib-Thrombolysis in Myocardial Infarction SOLID-TIMI 52. trial in patients after an acute coronary syndrome. Am Heart J 2011;1624:613–9. 10.1016/j.ahj.2011.07.018 21982651

[13] O'Donoghue ML, Braunwald E, White HD, Steen DP, Lukas MA, Tarka E, et al Effect of darapladib on major coronary events after an acute coronary syndrome: The SOLID-TIMI 52 randomized clinical trial. JAMA 2014;31210:1006–15. 10.1001/jama.2014.11061 25173516

[14] White HD, Held C, Stewart R, Tarka E, Brown R, Davies RY, et al Darapladib for preventing ischemic events in stable coronary heart disease. New Engl J Med 2014;37018:1702–11. 10.1056/NEJMoa1315878 24678955

[15] Stafforini DM, Satoh K, Atkinson DL, Tjoelker LW, Eberhardt C, Yoshida H, et al Platelet-activating factor acetylhydrolase deficiency. A missense mutation near the active site of an anti-inflammatory phospholipase. J Clin Invest 1996;9712:2784–91. 10.1172/JCI118733 PMC507371 8675689

[16] Wang Q, Hao Y, Mo X, Wang L, Lu X, Huang J, et al PLA2G7 gene polymorphisms and coronary heart disease risk: A meta-analysis. Thromb Res 2010;1266:498–503. 10.1016/j.thromres.2010.09.009 20926117

[17] Zheng GH, Chen HY, Xiong SQ, Chu JF. Lipoprotein-associated phospholipase A2 gene V279F polymorphisms and coronary heart disease: A meta-analysis. Mol Biol Rep 2011;386:4089–99. 10.1007/s11033-010-0529-9 21107710

[18] Jang Y, Waterworth D, Lee JE, Song K, Kim S, Kim HS, et al Carriage of the V279F null allele within the gene encoding Lp-PLA2. is protective from coronary artery disease in South Korean males. PLoS One 2011;64:e18208. 10.1371/journal.pone.0018208 PMC3071750 21490708

[19] Millwood IY, Bennett DA, Walters RG, Clarke R, Waterworth D, Johnson T, et al Lipoprotein-Associated Phospholipase A2 Loss-of-Function Variant and Risk of Vascular Diseases in 90,000 Chinese Adults. J Am Coll Cardiol 2016;672:230–1. 10.1016/j.jacc.2015.10.056 PMC4710575 26791069

[20] Gregson JM, Freitag DF, Surendran P, Stitziel NO, Chowdhury R, Burgess S, et al Genetic invalidation of Lp-PLA_2_ as a therapeutic target: Large-scale study of five functional Lp-PLA_2_-lowering alleles. Eur J Prev Cardiol 2017;ePub ahead of print 8 Dec 2016. 10.1177/2047487316682186 PMC5460752 27940953

[21] Suchindran S, Rivedal D, Guyton JR, Milledge T, Gao X, Benjamin A, et al Genome-wide association study of Lp-PLA2. activity and mass in the Framingham Heart Study. PLoS Genet 2010;64:e1000928. 10.1371/journal.pgen.1000928 PMC2861686 20442857

[22] Grallert H, Dupuis J, Bis JC, Dehghan A, Barbalic M, Baumert J, et al Eight genetic loci associated with variation in lipoprotein-associated phospholipase A2 mass and activity and coronary heart disease: meta-analysis of genome-wide association studies from five community-based studies. Eur Heart J 2012;332:238–51. 10.1093/eurheartj/ehr372 PMC3258449 22003152

[23] Chu AY, Guilianini F, Grallert H, Dupuis J, Ballantyne CM, Barratt BJ, et al Genome-wide association study evaluating lipoprotein-associated phospholipase A2 mass and activity at baseline and after rosuvastatin therapy. Circ Cardiovasc Genet 2012;56:676–85. 10.1161/CIRCGENETICS.112.963314 23118302

[24] Wallentin L, Held C, Armstrong PW, Cannon CP, Davies RY, Granger CB, et al Lipoprotein‐Associated Phospholipase A_2_ Activity Is a Marker of Risk But Not a Useful Target for Treatment in Patients With Stable Coronary Heart Disease. J Am Heart Assoc 2016;5:e003407 doi: 10.1161/JAHA.116.003407 27329448 10.1161/JAHA.116.003407 PMC4937279

[25] Ichihara S, Yamada Y, Yokota M. Association of a G994—>T missense mutation in the plasma platelet-activating factor acetylhydrolase gene with genetic susceptibility to nonfamilial dilated cardiomyopathy in Japanese. Circulation 1998;9818:1881–5. 10.1161/01.cir.98.18.1881 9799208

[26] Satoh K, Yoshida H, Imaizumi T, Takamatsu S, Mizuno S. Platelet-activating factor acetylhydrolase in plasma lipoproteins from patients with ischemic stroke. Stroke 1992;238:1090–2. 10.1161/01.str.23.8.1090 1636183

[27] Shimokata K, Yamada Y, Kondo T, Ichihara S, Izawa H, Nagata K, et al Association of gene polymorphisms with coronary artery disease in individuals with or without nonfamilial hypercholesterolemia. Atherosclerosis 2004;1721:167–73. 10.1016/j.atherosclerosis.2003.09.019 14709372

[28] Subramanian VS, Goyal J, Miwa M, Sugatami J, Akiyama M, Liu M, et al Role of lecithin-cholesterol acyltransferase in the metabolism of oxidized phospholipids in plasma: studies with platelet-activating factor-acetyl hydrolase-deficient plasma. Biochim Biophys Acta 1999;14391:95–109. 10.1016/s1388-1981(99)00072-4 10395969

[29] Unno N, Nakamura T, Kaneko H, Uchiyama T, Yamamoto N, Sugatani J, et al Plasma platelet-activating factor acetylhydrolase deficiency is associated with atherosclerotic occlusive disease in japan. J Vasc Surg 2000;322:263–7. 10.1067/mva.2000.105670 10917985

[30] Unno N, Nakamura T, Mitsuoka H, Uchiyama T, Yamamoto N, Saito T, et al Association of a G994 —>T missense mutation in the plasma platelet-activating factor acetylhydrolase gene with risk of abdominal aortic aneurysm in Japanese. Ann Surg 2002;2352:297–302. 10.1097/00000658-200202000-00020 PMC1422429 11807372

[31] Wang T, Karino K, Yamasaki M, Zhang Y, Masuda J, Yamaguchi S, et al Effects of G994T in the Lp-PLA2 gene on the plasma oxidized LDL level and carotid intima-media thickness in Japanese: the Shimane study. Am J Hypertens 2009;227:742–7. 10.1038/ajh.2009.70 19373214

[32] Yamada Y, Yokota M. Loss of activity of plasma platelet-activating factor acetylhydrolase due to a novel Gln281—>Arg mutation. Biochem Biophys Res Commun 1997;2363:772–5. 10.1006/bbrc.1997.7047 9245731

[33] Yamada Y, Ichihara S, Fujimura T, Yokota M. Identification of the G994—> T missense in exon 9 of the plasma platelet-activating factor acetylhydrolase gene as an independent risk factor for coronary artery disease in Japanese men. Metabolism 1998;472:177–81. 10.1016/s0026-0495(98)90216-5 9472966

[34] Yamada Y, Yoshida H, Ichihara S, Imaizumi T, Satoh K, Yokota M. Correlations between plasma platelet-activating factor acetylhydrolase PAF-AH. activity and PAF-AH genotype, age, and atherosclerosis in a Japanese population. Atherosclerosis 2000;1501:209–16. 10.1016/s0021-9150(99)00385-8 10781653

[35] Yamada Y, Ichihara S, Izawa H, Tanaka M, Yokota M. Association of a G994 —> T Val279 —> Phe. polymorphism of the plasma platelet-activating factor acetylhydrolase gene with myocardial damage in Japanese patients with nonfamilial hypertrophic cardiomyopathy. J Hum Genet 2001;468:436–41. 10.1007/s100380170042 11501940

[36] Zhang SY, Shibata H, Karino K, Wang BY, Kobayashi S, Masuda J, et al Comprehensive evaluation of genetic and environmental factors influencing the plasma lipoprotein-associated phospholipase A2 activity in a Japanese population. Hypertens Res 2007;305:403–9. 10.1291/hypres.30.403 17587752

[37] Teslovich TM, Musunuru K, Smith AV, Edmondson AC, Stylianou IM, Koseki M, et al Biological, clinical and population relevance of 95 loci for blood lipids. Nature 2010;4667307:707–13. 10.1038/nature09270 PMC3039276 20686565

[38] Cusanovich DA, Billstrand C, Zhou X, Chavarria C, De Leon S, Michelini K, et al The combination of a genome-wide association study of lymphocyte count and analysis of gene expression data reveals novel asthma candidate genes. Hum Mol Genet 2012;219:2111–23. 10.1093/hmg/dds021 PMC3315207 22286170

[39] Saxena R, Voight BF, Lyssenko V, Burtt NP, De Bakker PIW, Chen H, et al Genome-wide association analysis identifies loci for type 2 diabetes and triglyceride levels. Science 2007;3165829:1331–6. 10.1126/science.1142358 17463246

[40] Kathiresan S, Manning AK, Demissie S, D'Agostino RB, Surti A, Guiducci C, et al A genome-wide association study for blood lipid phenotypes in the Framingham Heart Study. BMC Med Genet 2007;8 SUPPL. 1. 10.1186/1471-2350-8-S1-S17 PMC1995614 17903299

[41] Kathiresan S, Willer CJ, Peloso GM, Demissie S, Musunuru K, Schadt EE, et al Common variants at 30 loci contribute to polygenic dyslipidemia. Nat Genet 2009;411:56–65. 10.1038/ng.291 PMC2881676 19060906

[42] Wojczynski MK, Li M, Bielak LF, Kerr KF, Reiner AP, Wong ND, et al Genetics of coronary artery calcification among African Americans, a meta-analysis. BMC Med Genet 2013;141. 10.1186/1471-2350-14-75 PMC3733595 23870195

[43] Nikpay M, Goel A, Won H, Hall LM, Willenborg C, Kanoni S, et al A comprehensive 1000 Genomes–based genome-wide association meta-analysis of coronary artery disease. Nat Genet. 2015;47:1121–1130. doi: 10.1038/ng.3396 26343387 10.1038/ng.3396 PMC4589895

[44] Nelson MR, Johnson T, Warren L, Hughes AR, Chissoe SL, Xu CF, et al The genetics of drug efficacy: opportunities and challenges. Nat Rev Genet 2016;174:197–206. 10.1038/nrg.2016.12 26972588

[45] Purcell S, Neale B, Todd-Brown K, Thomas L, Ferreira MA, Bender D, et al PLINK: a tool set for whole-genome association and population-based linkage analyses. Am J Hum Genet 2007;813:559–75. 10.1086/519795 PMC1950838 17701901

[46] Manichaikul A, Mychaleckyj JC, Rich SS, Daly K, Sale M, Chen WM. Robust relationship inference in genome-wide association studies. Bioinformatics 2010;2622:2867–73. 10.1093/bioinformatics/btq559 PMC3025716 20926424

[47] Zheng X, Levine D, Shen J, Gogarten SM, Laurie C, Weir BS. A high-performance computing toolset for relatedness and principal component analysis of SNP data. Bioinformatics 2012;2824:3326–8. 10.1093/bioinformatics/bts606 PMC3519454 23060615

[48] Goldstein JI, Crenshaw A, Carey J, Grant GB, Maguire J, Fromer M, et al zCall: a rare variant caller for array-based genotyping: genetics and population analysis. Bioinformatics 201;2819:2543–5. 10.1093/bioinformatics/bts479 PMC3463112 22843986

[49] Delaneau O, Marchini J, Zagury JF. A linear complexity phasing method for thousands of genomes. Nat Methods 2012;92:179–81. 10.1038/nmeth.1785 22138821

[50] Li Y, Willer C, Sanna S, Abecasis Ga. Genotype Imputation. Annu Rev Genom Human Genet 2009 8 28;101:387–406. 10.1146/annurev.genom.9.081307.164242 PMC2925172 19715440

[51] Marchini J, Howie B. Genotype imputation for genome-wide association studies. Nat Rev Genet 2010;117:499–511. 10.1038/nrg2796 20517342

[52] Williams AL, Patterson N, Glessner J, Hakonarson H, Reich D. Phasing of many thousands of genotyped samples. Am J Hum Genet 2012;912:238–51. 10.1016/j.ajhg.2012.06.013 PMC3415548 22883141

[53] Willer CJ, Li Y, Abecasis GR. METAL: Fast and efficient meta-analysis of genomewide association scans. Bioinformatics 2010;2617:2190–1. 10.1093/bioinformatics/btq340 PMC2922887 20616382

