# Supplementary material for: Pharmacogenetic meta-analysis of baseline risk factors, pharmacodynamic, efficacy and tolerability endpoints from two large global cardiovascular outcomes trials for darapladib
Source: PLoS One. 2017 Jul 28;12(7):e0182115. doi: 10.1371/journal.pone.0182115 (PMC5533343; doi:10.1371/journal.pone.0182115)
Supplement: S1 Text — Details on differences in reporting of odor events and functional insights for variants associated with efficacy and tolerability endpoints. (DOCX) [file pone.0182115.s001.docx]

# Supporting Text

## ****Differences in reporting of odor events****

**Some regional differences in reporting of the odor events were observed, with an** increased chance of odor events (treatment effect varied from 2 to 5 times) in the subjects treated with darapladib by region, possibly due to cultural differences in reporting such events, with higher events rates in North America (23-25%) and Western Europe (13-15%) compared to Asia (7-9%) and Eastern Europe (3-5%) (S6 Table). Although a variable event rate was also observed for diarrhea, there was consistent treatment effect by region (treatment effect was constant with 2 times increased chance of diarrhea event by darapladib by region compared to placebo).

## Functional insights for variants associated with efficacy and tolerability endpoints

**As the majority of the top-associated variants at the efficacy and tolerability loci were not located within genes, we reviewed public databases to assess any possible impact on gene regulation, to see if this could provide a biological rationale.**

**Review of the four efficacy loci suggested the presence of enhancer histone marks in monocytes at the location of chromosome 1 variants rs192476688 and rs201052613 [1]. No potential regulatory features were identified for the other three loci. More investigation will be required to fully understand what specifically is underlying these associations.**

**Review of the diarrhea association with an intragenic chromosome 9 variant rs62568141 (minor allele frequency approximately 3%) highlighted that this conserved variant may change some regulatory motifs and has some interesting correlations with promoter histone marks in intestinal tissues in public databases, such as HaploReg v3.0 and RegulomeDB [1;2]. Although there is no direct evidence of gene regulation impact for the associated variants at chromosome 7 and 16 loci, there is some suggestive evidence for increased expression of the *TMEM231* gene at the chromosome 16 locus in Crohn’s disease and ulcerative colitis [3] and also for the *GLCCI1* gene at the chromosome 7 locus in inflammatory bowel disease [4;5].**

# ****References****

**(1) Boyle AP, Hong EL, Hariharan M, Cheng Y, Schaub MA, Kasowski M, et al. Annotation of functional variation in personal genomes using RegulomeDB. 2012;22(9):1790-7.**

**(2) Ward LD, Kellis M. HaploReg: A resource for exploring chromatin states, conservation, and regulatory motif alterations within sets of genetically linked variants. 2012;40(D1):D930-D934.**

**(3) Funke B, Lasitschka F, Roth W, Penzel R, Meuer S, Saile M, et al. Selective downregulation of retinoic acid-inducible gene i within the intestinal epithelial compartment in crohn's disease. 2011;17(9):1943-54.**

**(4) Galamb O, Gyorffy B, Sipos F, Spisak S, Nemeth AM, Miheller P, et al. Inflammation, adenoma and cancer: Objective classification of colon biopsy specimens with gene expression signature. Disease Markers 2008;25(1):1-16.**

**(5) Gyorffy B, Molnar B, Lage H, Szallasi Z, Eklund AC. Evaluation of microarray preprocessing algorithms based on concordance with RT-PCR in clinical samples. 2009;4(5).**
