# Supplementary material for: Pharmacogenetic meta-analysis of baseline risk factors, pharmacodynamic, efficacy and tolerability endpoints from two large global cardiovascular outcomes trials for darapladib
Source: PLoS One. 2017 Jul 28;12(7):e0182115. doi: 10.1371/journal.pone.0182115 (PMC5533343; doi:10.1371/journal.pone.0182115)

S1 Fig. Power plots. Power for a) Baseline Lp-PLA2 activity in both placebo and darapladib arms (n=23,000) and Change from Baseline in darapladib arm (n = 11,500) at alpha = 5E-8; b) efficacy endpoints in both placebo and darapladib arms (n = 23,000) at alpha = 0.001 (candidate gene) and 5E-8 (GWAS) tolerability endpoints in darapladib arm only arms (n = 11,500) at alpha = 0.001 (candidate gene) and 5E-8 (GWAS).

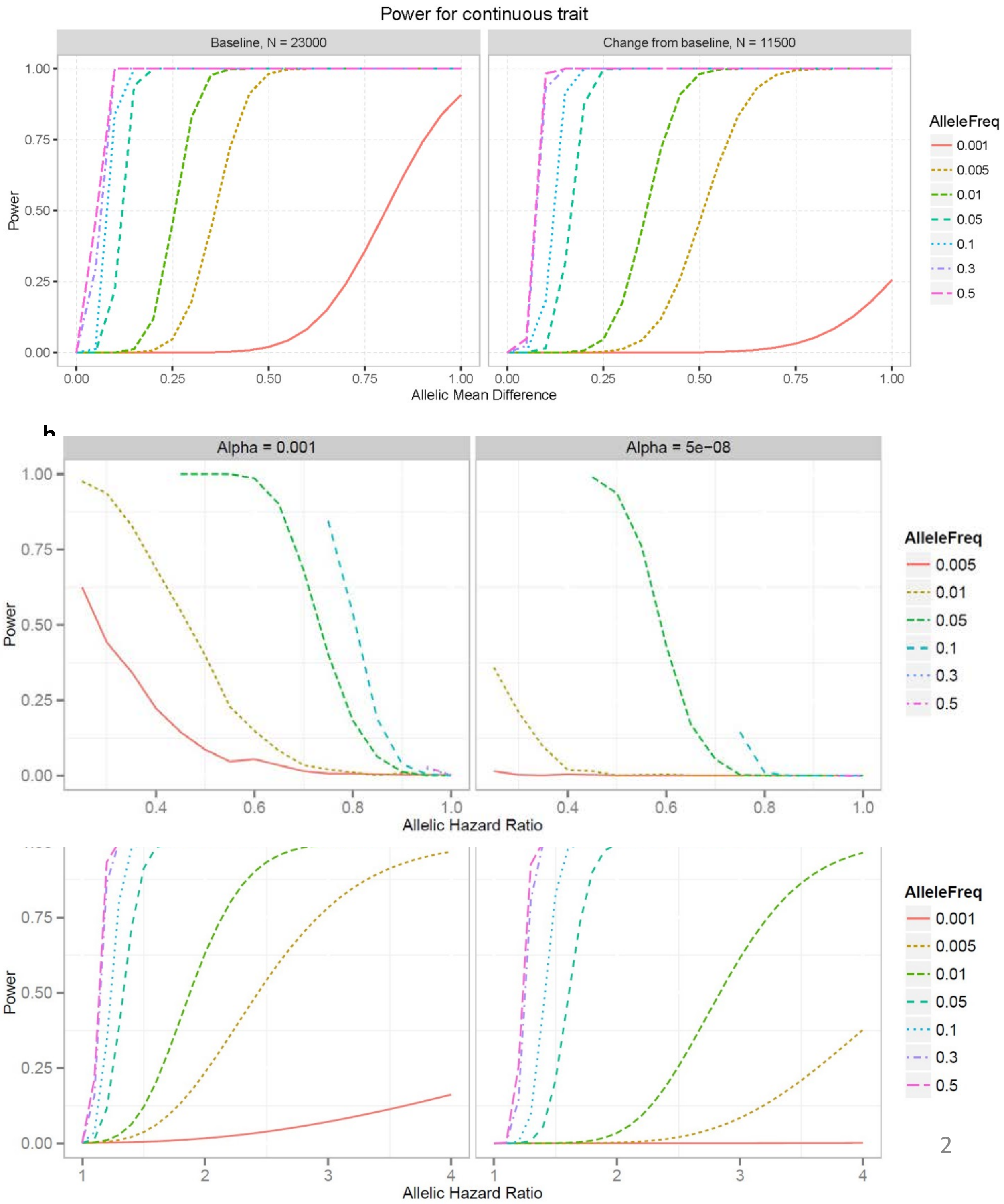

Supplement: S1 Fig — Power for a) Baseline Lp-PLA2 activity in both placebo and darapladib arms (n = 23,000) and Change from Baseline in darapladib arm (n = 11,500) at alpha = 5E-08; b) efficacy endpoints in both placebo and darapladib arms (n = 23,000) at alpha = 0.001 (candidate gene) and 5E-08 (GWAS) tolerability endpoints in darapladib arm only arms (n = 11,500) at alpha = 0.001 (candidate gene) and 5E-08 (GWAS). (PDF) [file pone.0182115.s002.pdf]
