# Supplementary figures and images for: Pharmacogenetic meta-analysis of baseline risk factors, pharmacodynamic, efficacy and tolerability endpoints from two large global cardiovascular outcomes trials for darapladib
Source: PLoS One. 2017 Jul 28;12(7):e0182115. doi: 10.1371/journal.pone.0182115 (PMC5533343; doi:10.1371/journal.pone.0182115)

**S2 Fig. Manhattan, QQ, and histogram for baseline Lp-PLA<sub>2</sub> activity.**

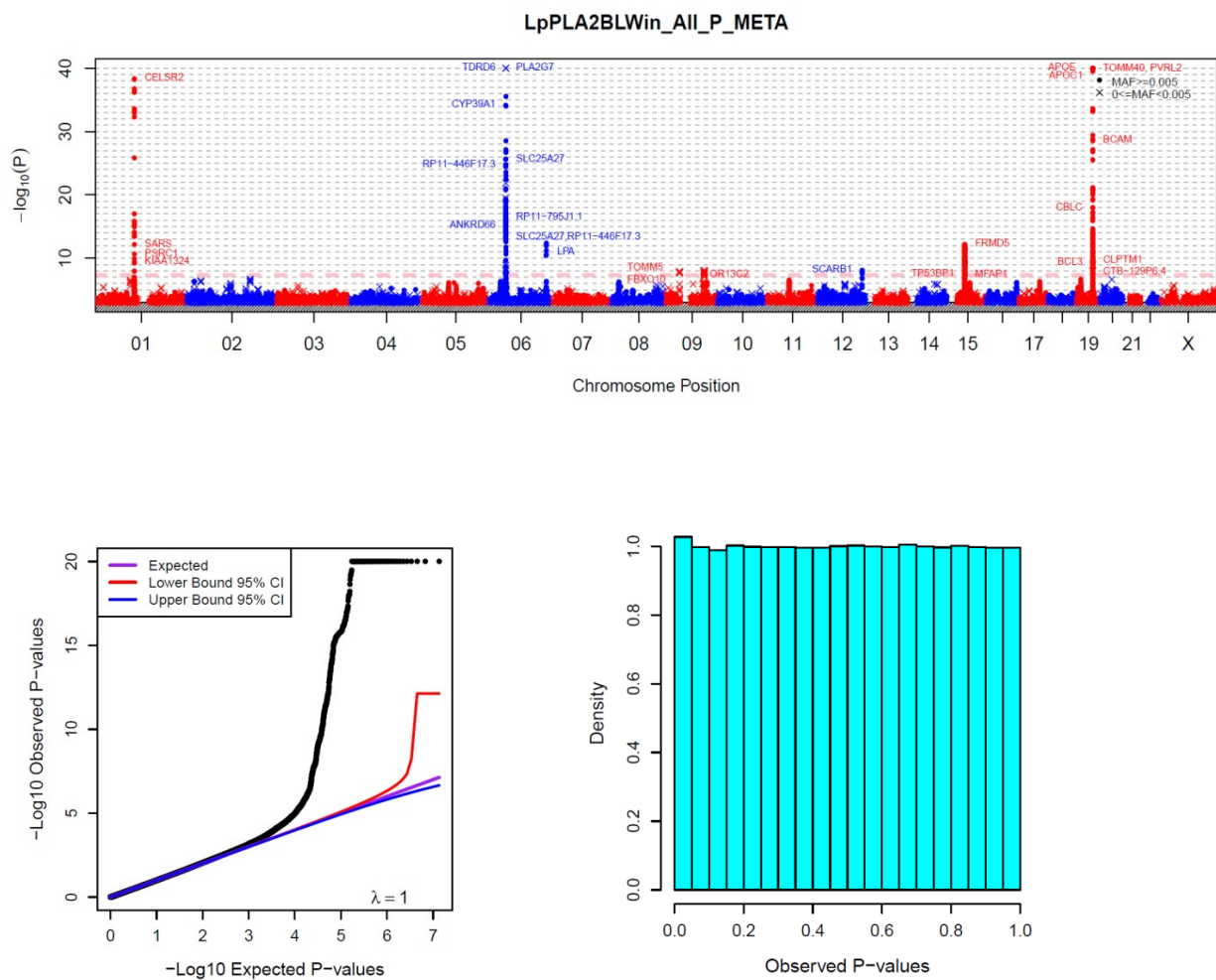

Supplement: S2 Fig — (PDF) [file pone.0182115.s003.pdf]
