# Supplementary material for: Pharmacogenetic meta-analysis of baseline risk factors, pharmacodynamic, efficacy and tolerability endpoints from two large global cardiovascular outcomes trials for darapladib
Source: PLoS One. 2017 Jul 28;12(7):e0182115. doi: 10.1371/journal.pone.0182115 (PMC5533343; doi:10.1371/journal.pone.0182115)
Supplement: S3 Fig — Regional, forest and box plots of mean difference (MD) of novel loci (with greater than 10 copies of variant alleles) associated with baseline Lp-PLA2 activity at genome-wide (a&b) and candidate gene (c) significance. The dashed line in the region plots indicates the significance threshold. (PDF) [file pone.0182115.s004.pdf]

S3 Fig. Regional, forest and box plots of novel loci associated with baseline Lp-PLA2 activity. Regional, forest and box plots of mean difference (MD) of novel loci (with greater than 10 copies of variant alleles) associated with baseline lipoprotein-associated phospholipase A2 activity at genome-wide (a&b) and candidate gene (c) significance. The dashed line in the region plots indicates the significance threshold.

a

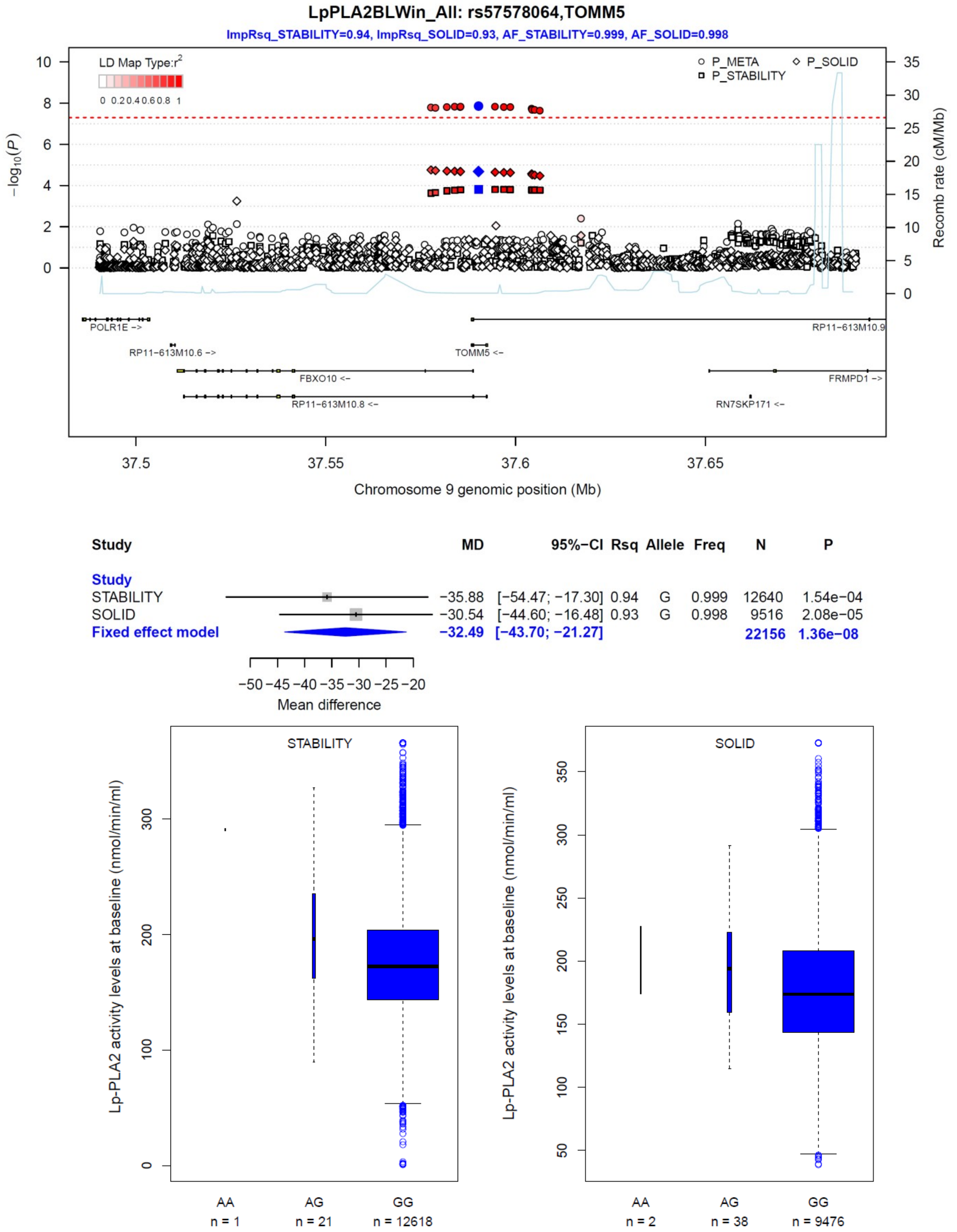

S3 Fig. b

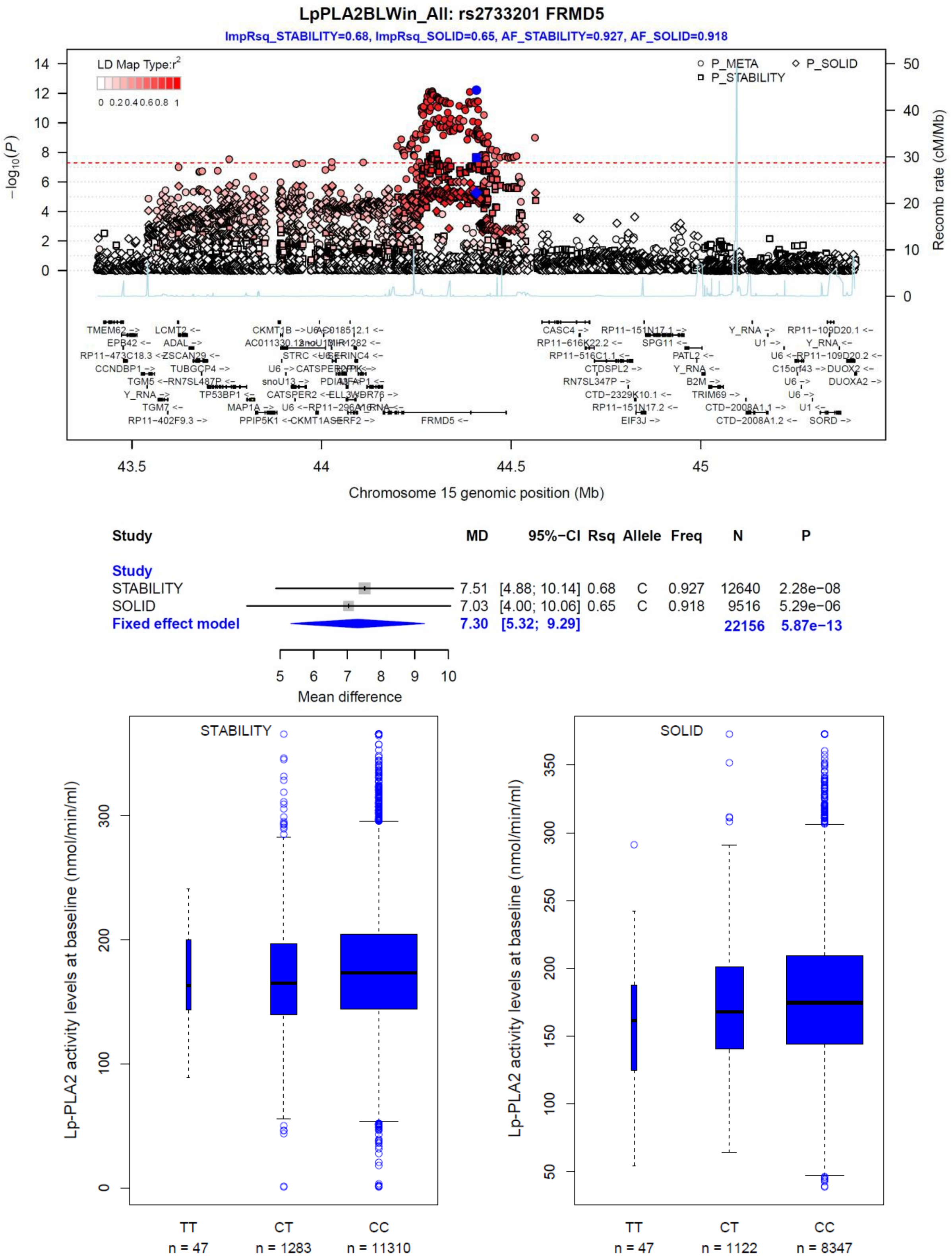
LpPLA2BLWin_All: rs2733201 FRMD5
ImpRsq_STABILITY=0.68, ImpRsq_SOLID=0.65, AF_STABILITY=0.927, AF_SOLID=0.918
LD Map Type:r2
0 0.2 0.4 0.6 0.8 1
P_META
P_STABILITY
P_SOLID
−log10(P)
Recomb rate (cM/Mb)
Chromosome 15 genomic position (Mb)
43.5
44
44.5
45
FRMD5 <-
Study MD 95%−CI Rsq Allele Freq N P
Study
STABILITY 7.51 [4.88; 10.14] 0.68 C 0.927 12640 2.28e−08
SOLID 7.03 [4.00; 10.06] 0.65 C 0.918 9516 5.29e−06
Fixed effect model 7.30 [5.32; 9.29] 22156 5.87e−13
Mean difference
STABILITY
SOLID
Lp-PLA2 activity levels at baseline (nmol/min/ml)
TT n = 47
CT n = 1283
CC n = 11310
TT n = 47
CT n = 1122
CC n = 8347

S3 Fig. c

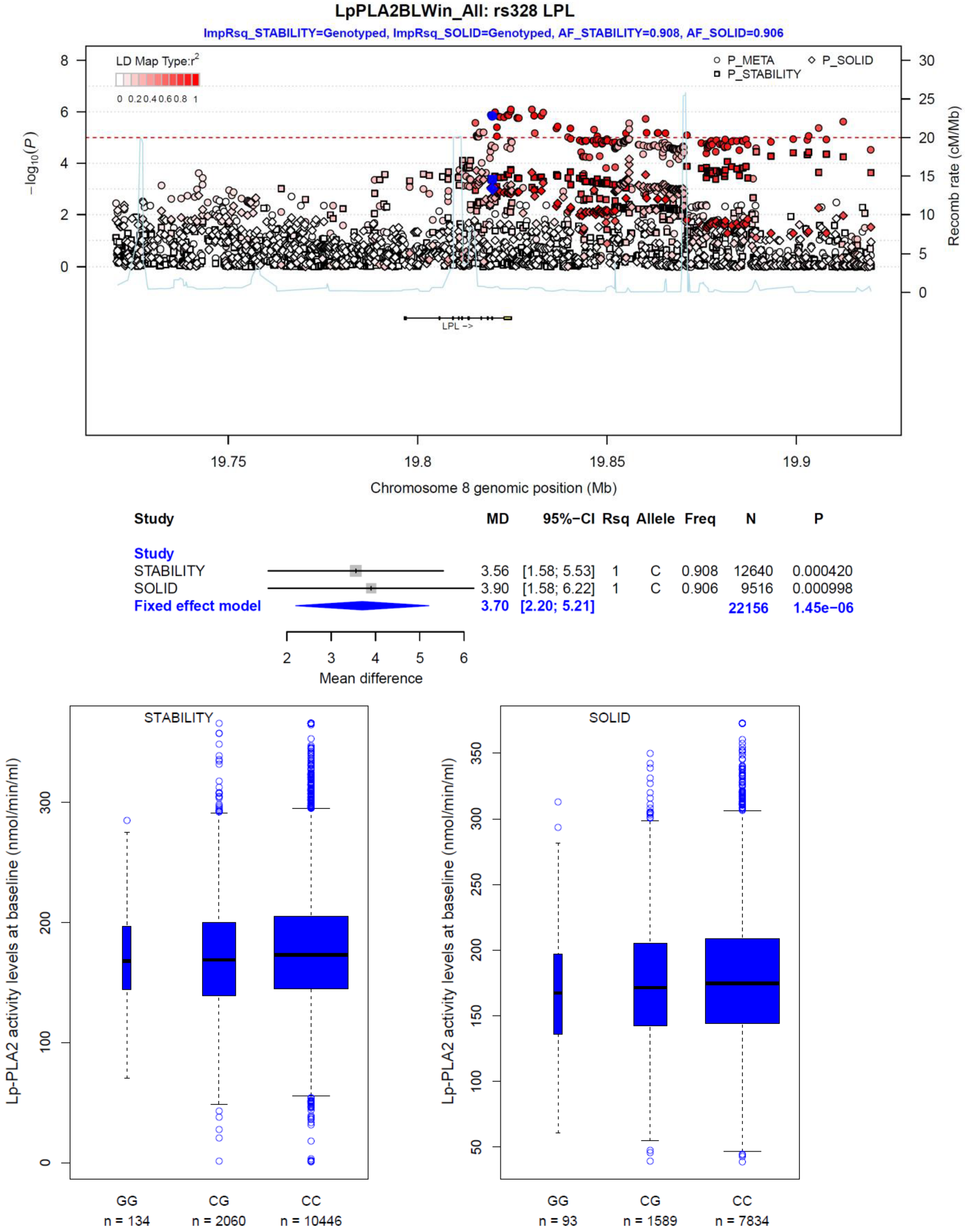
LpPLA2BLWin_All: rs328 LPL
ImpRsq_STABILITY=Genotyped, ImpRsq_SOLID=Genotyped, AF_STABILITY=0.908, AF_SOLID=0.906
LD Map Type:r2
0 0.2 0.4 0.6 0.8 1
P_META
P_SOLID
P_STABILITY
−log10(P)
Recomb rate (cM/Mb)
LPL ->
19.75
19.8
19.85
19.9
Chromosome 8 genomic position (Mb)
Study MD 95%−CI Rsq Allele Freq N P
Study
STABILITY 3.56 [1.58; 5.53] 1 C 0.908 12640 0.000420
SOLID 3.90 [1.58; 6.22] 1 C 0.906 9516 0.000998
Fixed effect model 3.70 [2.20; 5.21] 22156 1.45e−06
Mean difference
STABILITY
SOLID
Lp-PLA2 activity levels at baseline (nmol/min/ml)
GG
n = 134
CG
n = 2060
CC
n = 10446
GG
n = 93
CG
n = 1589
CC
n = 7834
