# Supplementary material for: Pharmacogenetic meta-analysis of baseline risk factors, pharmacodynamic, efficacy and tolerability endpoints from two large global cardiovascular outcomes trials for darapladib
Source: PLoS One. 2017 Jul 28;12(7):e0182115. doi: 10.1371/journal.pone.0182115 (PMC5533343; doi:10.1371/journal.pone.0182115)
Supplement: S4 Fig — For MCE: a) main genotype effect P-value in placebo arm, b) main genotype effect P-value in darapladib, c) genotype by treatment interaction P-value, and d) 2df test P-value for both main genotype and genotype by treatment interaction; For MI: e) main genotype effect P-value in placebo arm, f) main genotype effect P-value in darapladib, g) genotype by treatment interaction P-value, and h) 2df test P-value for both main genotype and genotype by treatment interaction. (PDF) [file pone.0182115.s005.pdf]

S4 Fig. Manhattan, QQ and histogram plots for efficacy endpoints. For MCE: a) main genotype effect p value in placebo arm, b) main genotype effect p value in darapladib, c) genotype by treatment interaction p value, and d) 2df test p value for both main genotype and genotype by treatment interaction; For MI: e) main genotype effect p value in placebo arm, f) main genotype effect p value in darapladib, g) genotype by treatment interaction p value, and h) 2df test p value for both main genotype and genotype by treatment interaction.

a

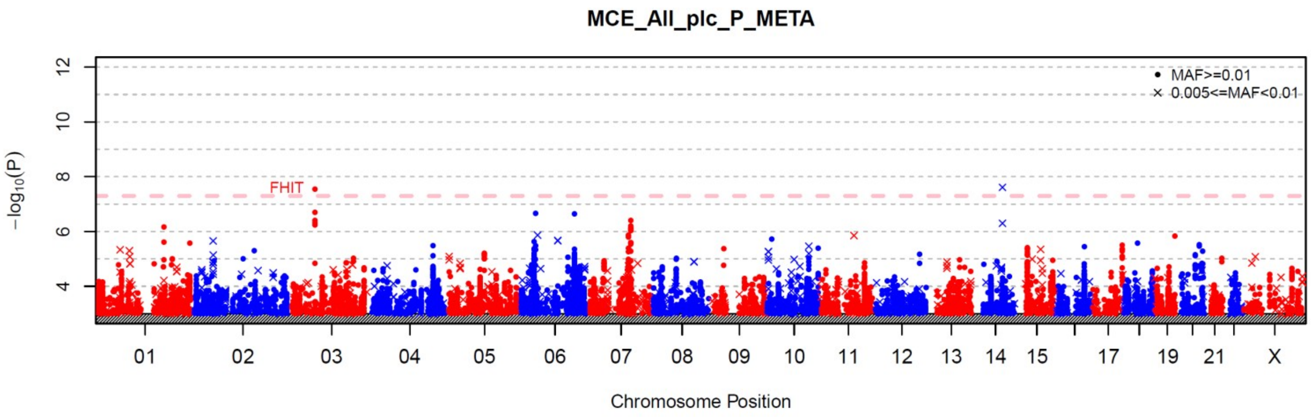

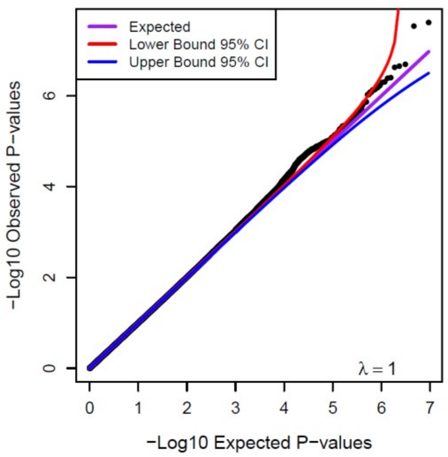

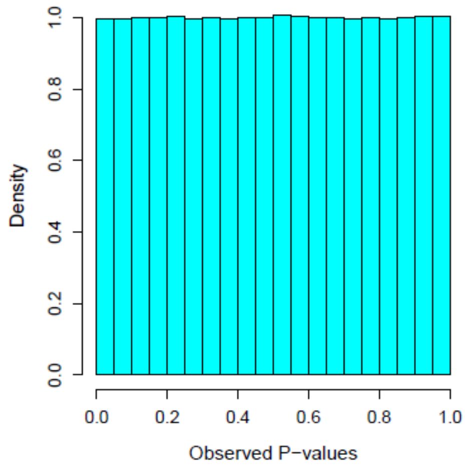

S4 Fig. b

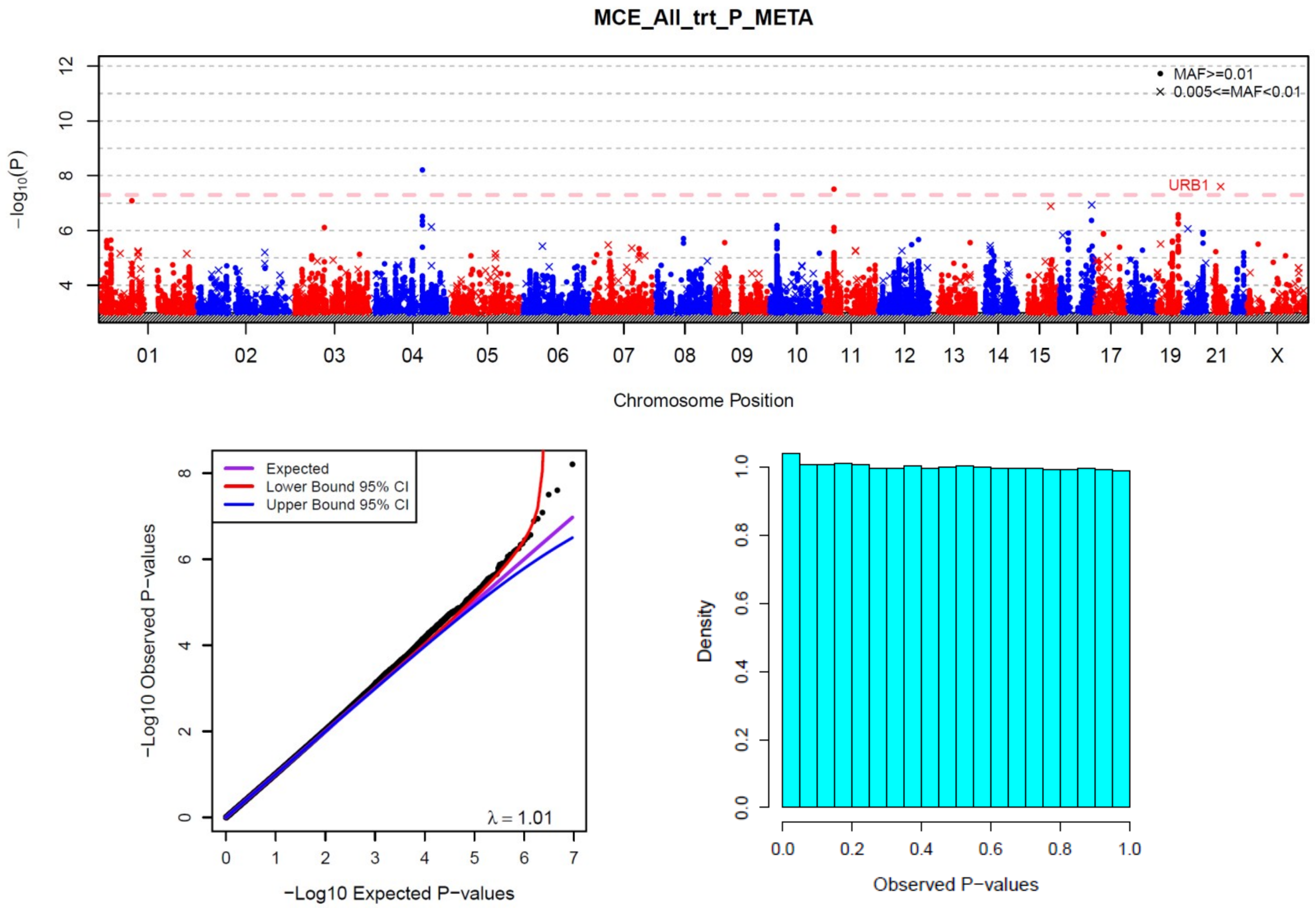
MCE_All_trt_P_META
MAF>=0.01
0.005<=MAF<0.01
URB1
−log10(P)
Chromosome Position
01
02
03
04
05
06
07
08
09
10
11
12
13
14
15
17
19
21
X
Expected
Lower Bound 95% CI
Upper Bound 95% CI
λ = 1.01
−Log10 Observed P−values
−Log10 Expected P−values
Density
Observed P−values

S4 Fig. c

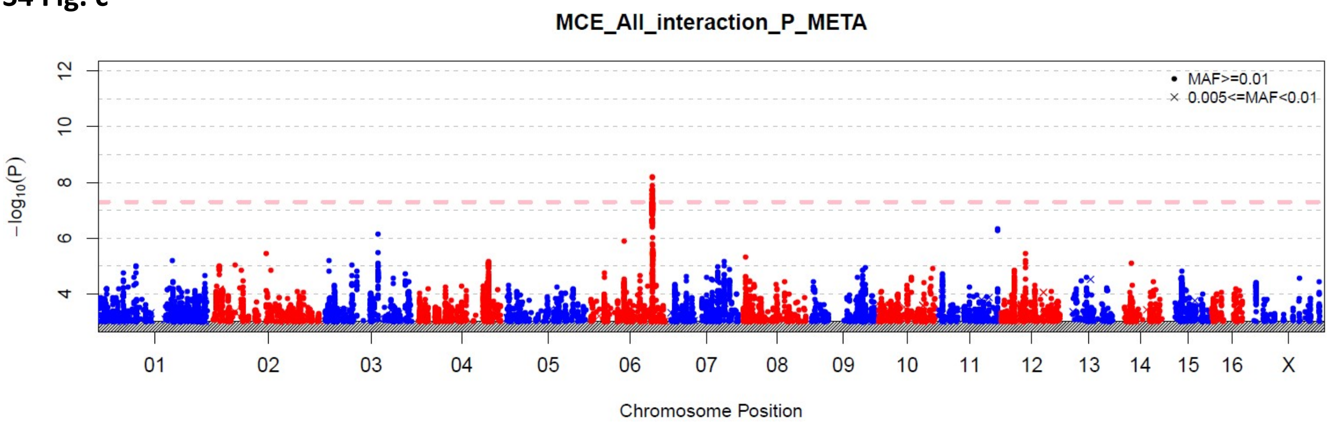
MCE_All_interaction_P_META
• MAF>=0.01
× 0.005<=MAF<0.01
−log10(P)
Chromosome Position
01
02
03
04
05
06
07
08
09
10
11
12
13
14
15
16
X

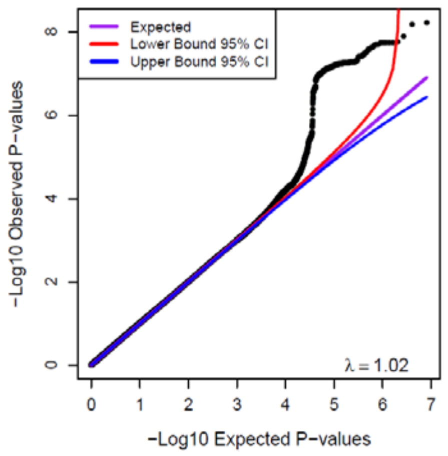
Expected
Lower Bound 95% CI
Upper Bound 95% CI
λ = 1.02
−Log10 Observed P−values
−Log10 Expected P−values

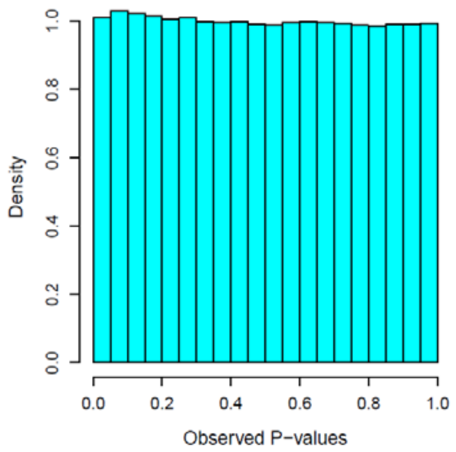
Density
Observed P−values

S4 Fig. d

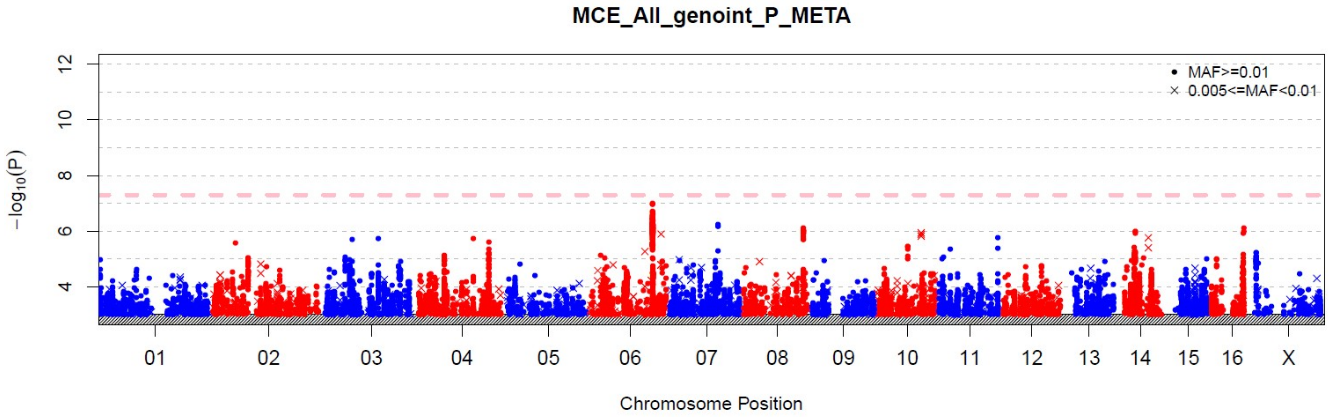
MCE_All_genoint_P_META
MAF>=0.01
0.005<=MAF<0.01
$-\log_{10}(P)$
Chromosome Position
01 02 03 04 05 06 07 08 09 10 11 12 13 14 15 16 X

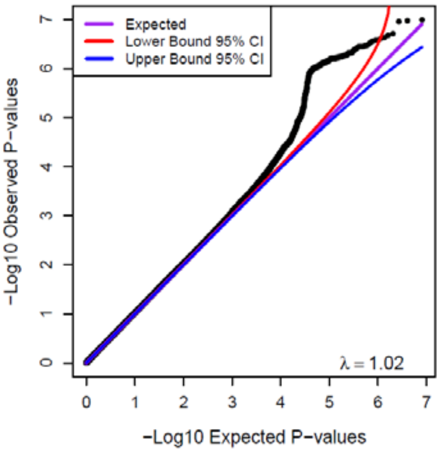
Expected
Lower Bound 95% CI
Upper Bound 95% CI
$\lambda = 1.02$
-Log10 Observed P-values
-Log10 Expected P-values

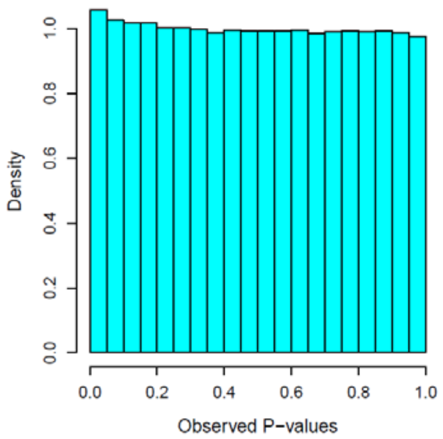
Density
Observed P-values

S4 Fig. e

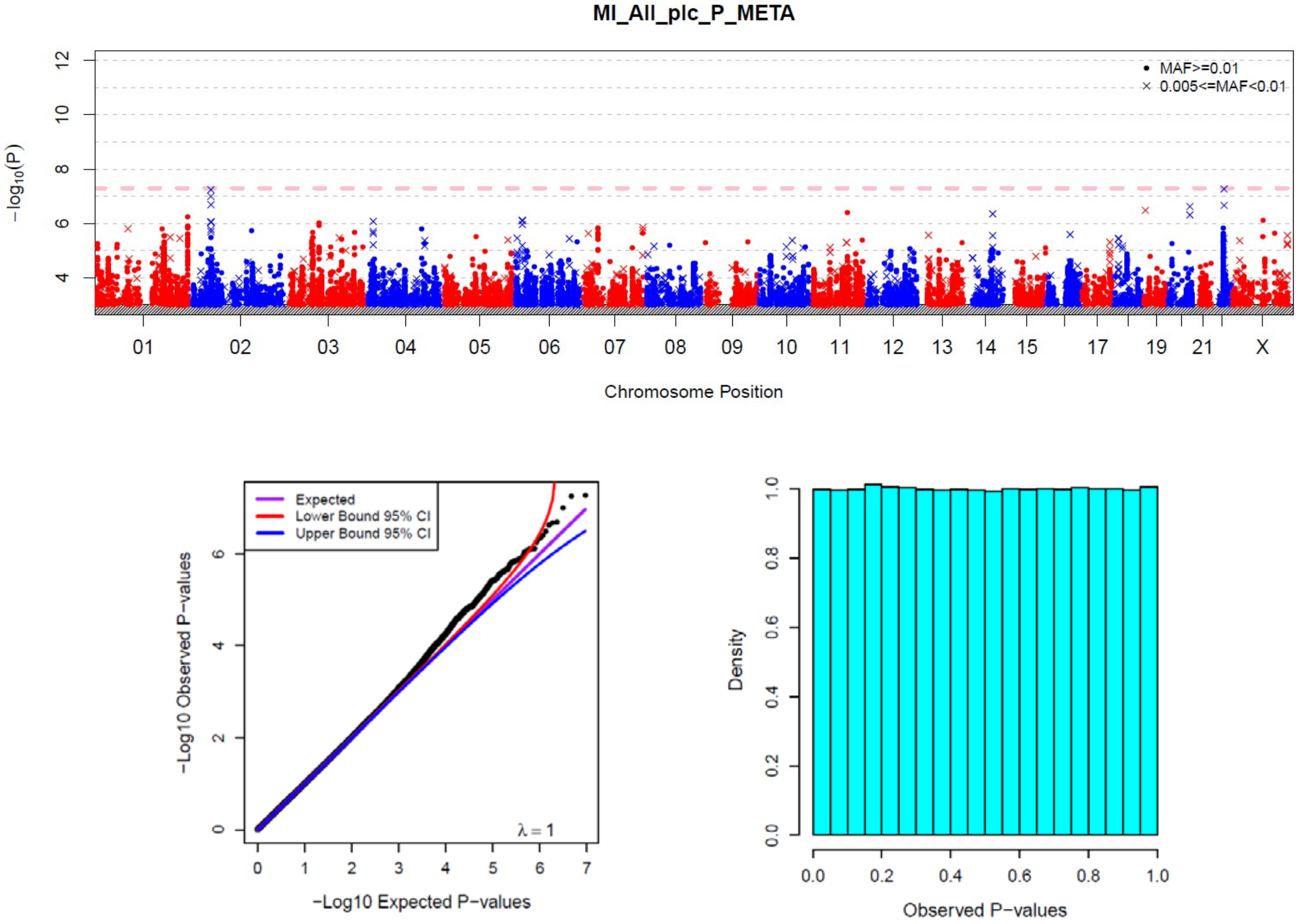
MI_All_plc_P_META
MAF>=0.01
0.005<=MAF<0.01
−log10(P)
12
10
8
6
4
01
02
03
04
05
06
07
08
09
10
11
12
13
14
15
17
19
21
X
Chromosome Position
Expected
Lower Bound 95% CI
Upper Bound 95% CI
−Log10 Observed P−values
−Log10 Expected P−values
λ = 1
0
1
2
3
4
5
6
7
Density
0.0
0.2
0.4
0.6
0.8
1.0
Observed P−values

S4 Fig. f

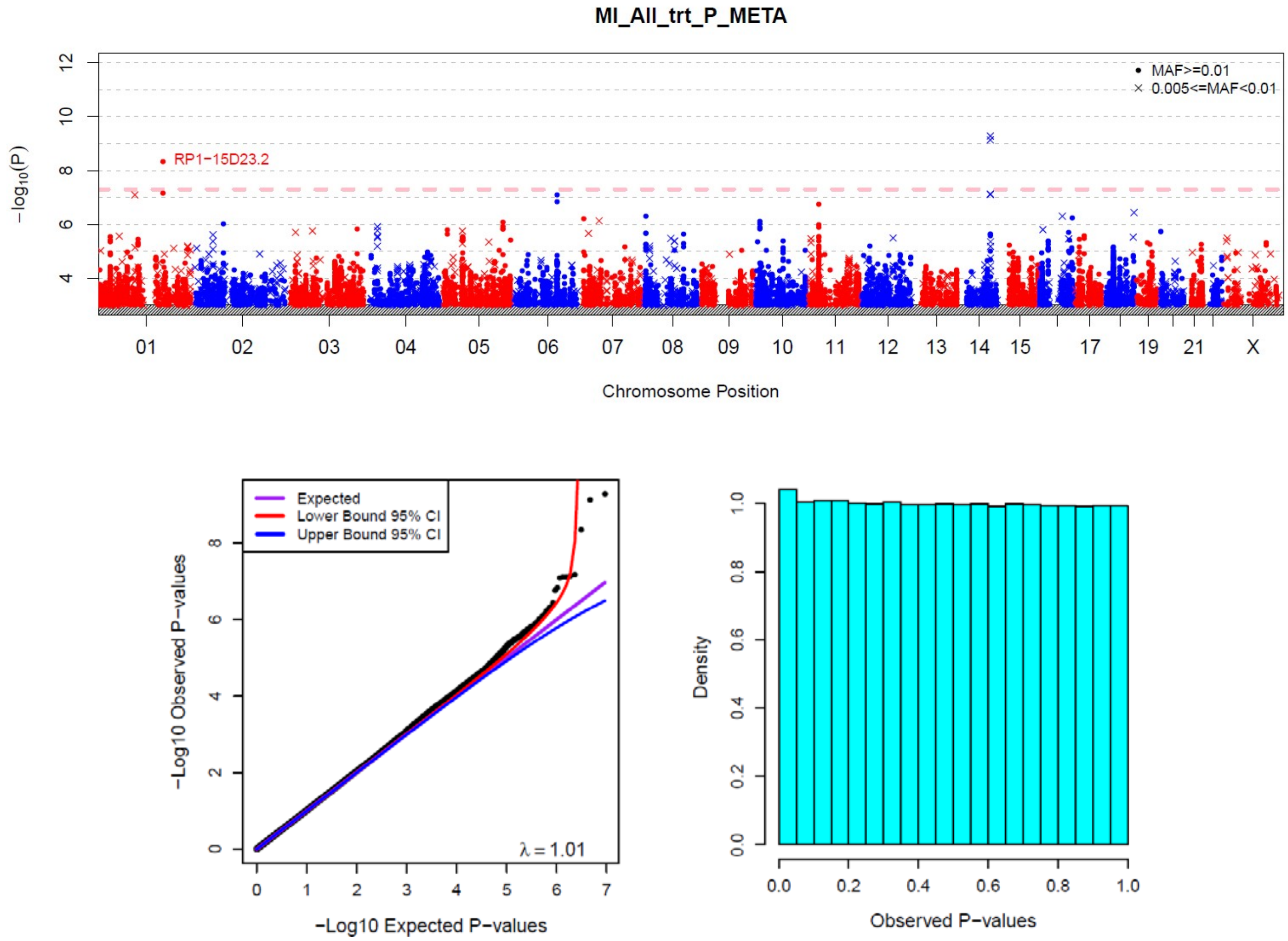
MI_All_trt_P_META
MAF>=0.01
0.005<=MAF<0.01
RP1−15D23.2
$-\log_{10}(P)$
Chromosome Position
01
02
03
04
05
06
07
08
09
10
11
12
13
14
15
17
19
21
X
Expected
Lower Bound 95% CI
Upper Bound 95% CI
λ = 1.01
−Log10 Observed P−values
−Log10 Expected P−values
Density
Observed P−values

S4 Fig. g

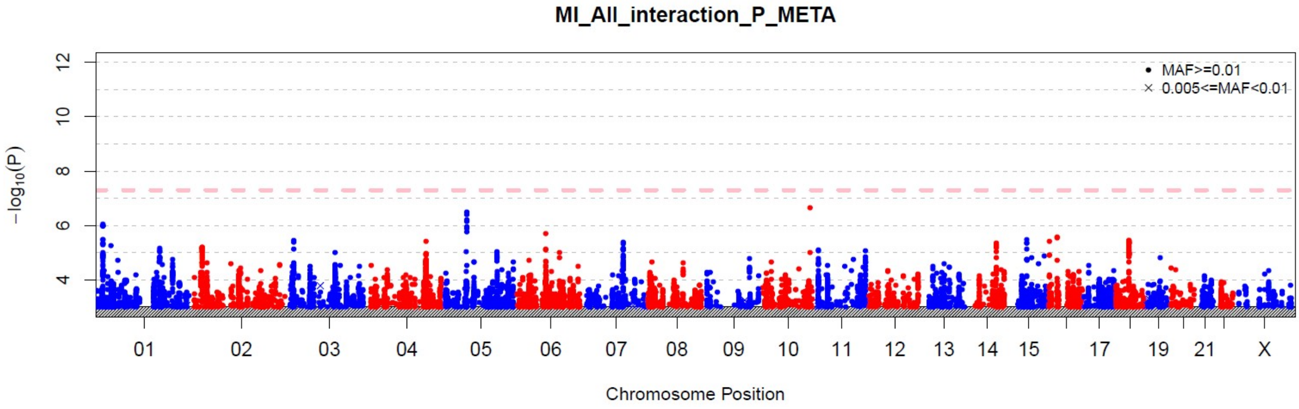

MI_All_interaction_P_META
MAF>=0.01
0.005<=MAF<0.01
$-\log_{10}(P)$
Chromosome Position
01 02 03 04 05 06 07 08 09 10 11 12 13 14 15 17 19 21 X

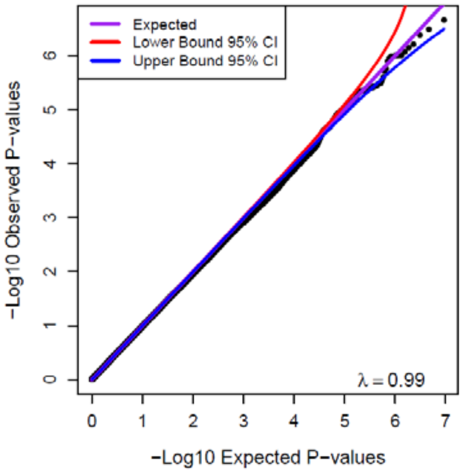

Expected
Lower Bound 95% CI
Upper Bound 95% CI
−Log10 Observed P−values
−Log10 Expected P−values
λ = 0.99

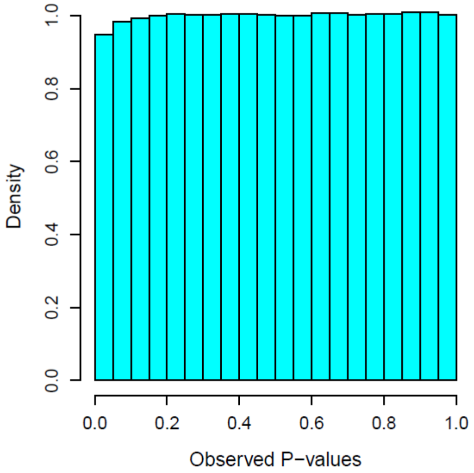

Density
Observed P−values

S4 Fig. h

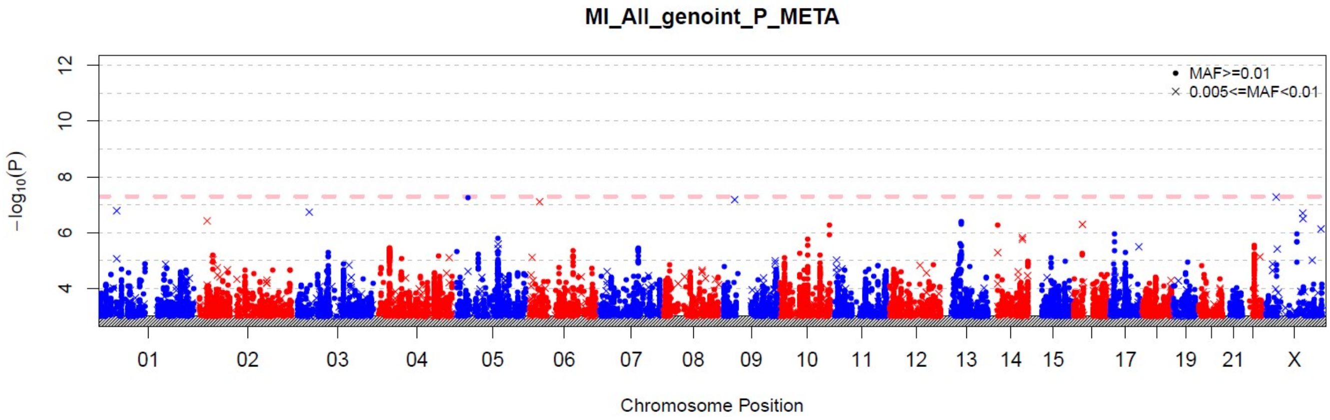
MI_All_genoint_P_META
MAF>=0.01
0.005<=MAF<0.01
$-\log_{10}(P)$
Chromosome Position
01 02 03 04 05 06 07 08 09 10 11 12 13 14 15 17 19 21 X

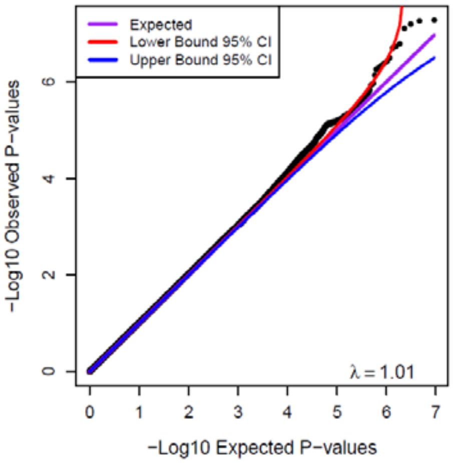
Expected
Lower Bound 95% CI
Upper Bound 95% CI
−Log10 Observed P−values
−Log10 Expected P−values
λ = 1.01

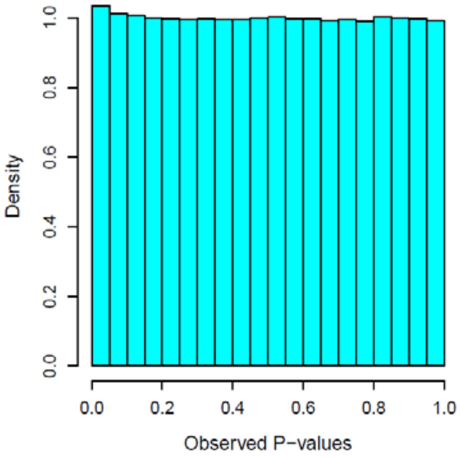
Density
Observed P−values
