# Supplementary material for: Pharmacogenetic meta-analysis of baseline risk factors, pharmacodynamic, efficacy and tolerability endpoints from two large global cardiovascular outcomes trials for darapladib
Source: PLoS One. 2017 Jul 28;12(7):e0182115. doi: 10.1371/journal.pone.0182115 (PMC5533343; doi:10.1371/journal.pone.0182115)
Supplement: S5 Fig — The dashed line in the regional plots indicates the significance threshold. a) rs138741635, b) rs192427471, c) rs181937009, d) rs12290663, e) rs147204125. (PDF) [file pone.0182115.s006.pdf]

S5 Fig. Regional, survival and forest plots of variants associated with MCE following placebo and darapladib treatment. The dashed line in the regional plots indicates the significance threshold. a) rs138741635 , b) rs192427471, c) rs181937009, d) rs12290663, e) rs147204125.

S5 Fig. a

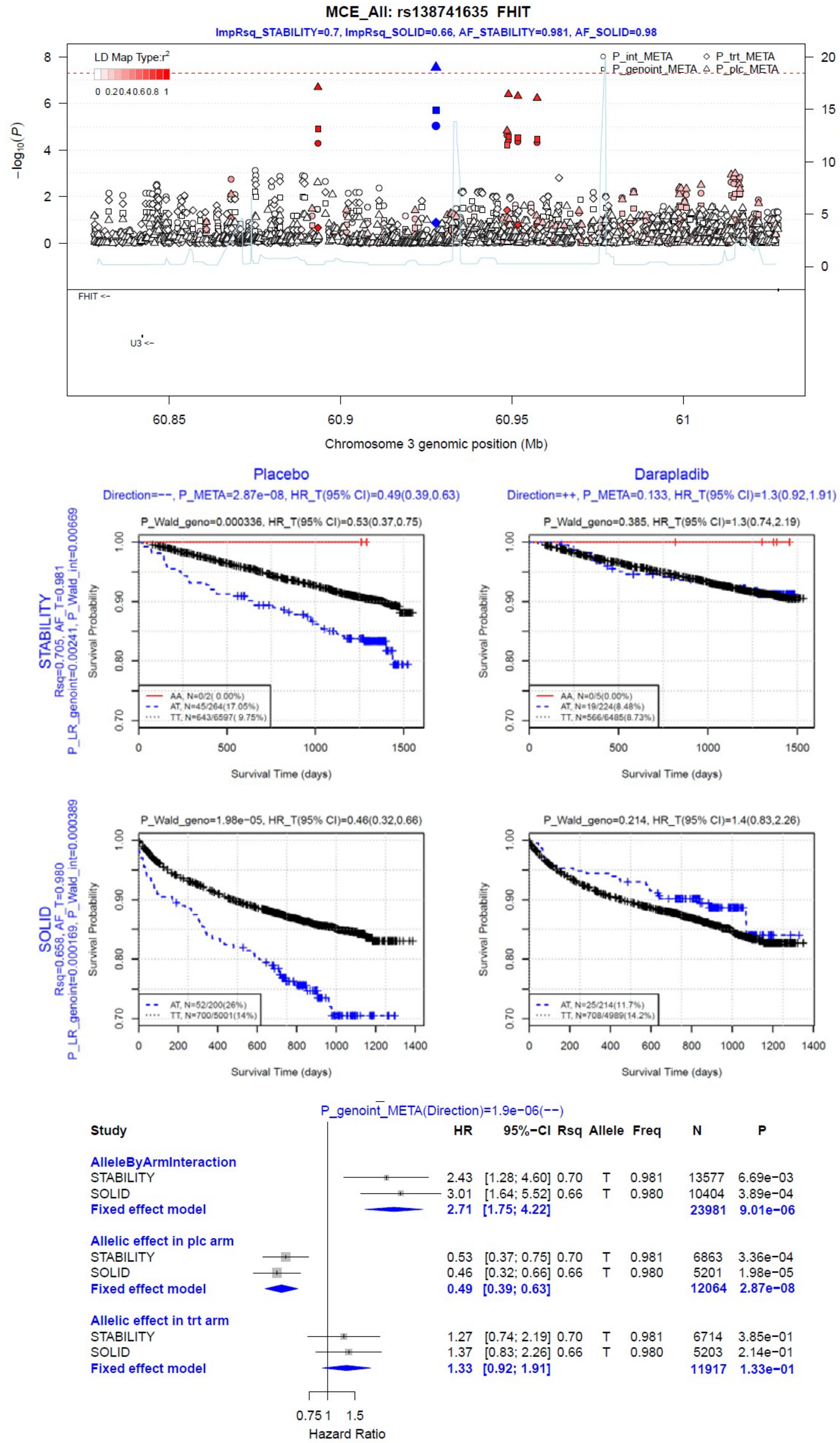
MCE_All: rs138741635 FHIT
ImpRsq_STABILITY=0.7, ImpRsq_SOLID=0.66, AF_STABILITY=0.981, AF_SOLID=0.98
LD Map Type:r2
0 0.20.40.60.8 1
P_int_META
P_trt_META
P_genoint_META
P_plc_META
−log10(P)
Recomb rate (cM/Mb)
FHIT <-
U3 <-
60.85
60.9
60.95
61
Chromosome 3 genomic position (Mb)
Placebo
Direction=−−, P_META=2.87e−08, HR_T(95% CI)=0.49(0.39,0.63)
Darapladib
Direction=++, P_META=0.133, HR_T(95% CI)=1.3(0.92,1.91)
STABILITY
Rsq=0.705, AF_T=0.981
P_LR_genoint=0.00241, P_Wald_int=0.00669
P_Wald_geno=0.000336, HR_T(95% CI)=0.53(0.37,0.75)
AA, N=0/2(0.00%)
AT, N=45/264(17.05%)
TT, N=643/6597(9.75%)
P_Wald_geno=0.385, HR_T(95% CI)=1.3(0.74,2.19)
AA, N=0/5(0.00%)
AT, N=19/224(8.48%)
TT, N=566/6485(8.73%)
Survival Probability
Survival Time (days)
SOLID
Rsq=0.658, AF_T=0.980
P_LR_genoint=0.000169, P_Wald_int=0.000389
P_Wald_geno=1.98e−05, HR_T(95% CI)=0.46(0.32,0.66)
AT, N=52/200(26%)
TT, N=700/5001(14%)
P_Wald_geno=0.214, HR_T(95% CI)=1.4(0.83,2.26)
AT, N=25/214(11.7%)
TT, N=708/4989(14.2%)
P_genoint_META(Direction)=1.9e−06(−−)
Study HR 95%−CI Rsq Allele Freq N P
AlleleByArmInteraction
STABILITY 2.43 [1.28; 4.60] 0.70 T 0.981 13577 6.69e−03
SOLID 3.01 [1.64; 5.52] 0.66 T 0.980 10404 3.89e−04
Fixed effect model 2.71 [1.75; 4.22] 23981 9.01e−06
Allelic effect in plc arm
STABILITY 0.53 [0.37; 0.75] 0.70 T 0.981 6863 3.36e−04
SOLID 0.46 [0.32; 0.66] 0.66 T 0.980 5201 1.98e−05
Fixed effect model 0.49 [0.39; 0.63] 12064 2.87e−08
Allelic effect in trt arm
STABILITY 1.27 [0.74; 2.19] 0.70 T 0.981 6714 3.85e−01
SOLID 1.37 [0.83; 2.26] 0.66 T 0.980 5203 2.14e−01
Fixed effect model 1.33 [0.92; 1.91] 11917 1.33e−01
0.75 1 1.5
Hazard Ratio

S5 Fig. b

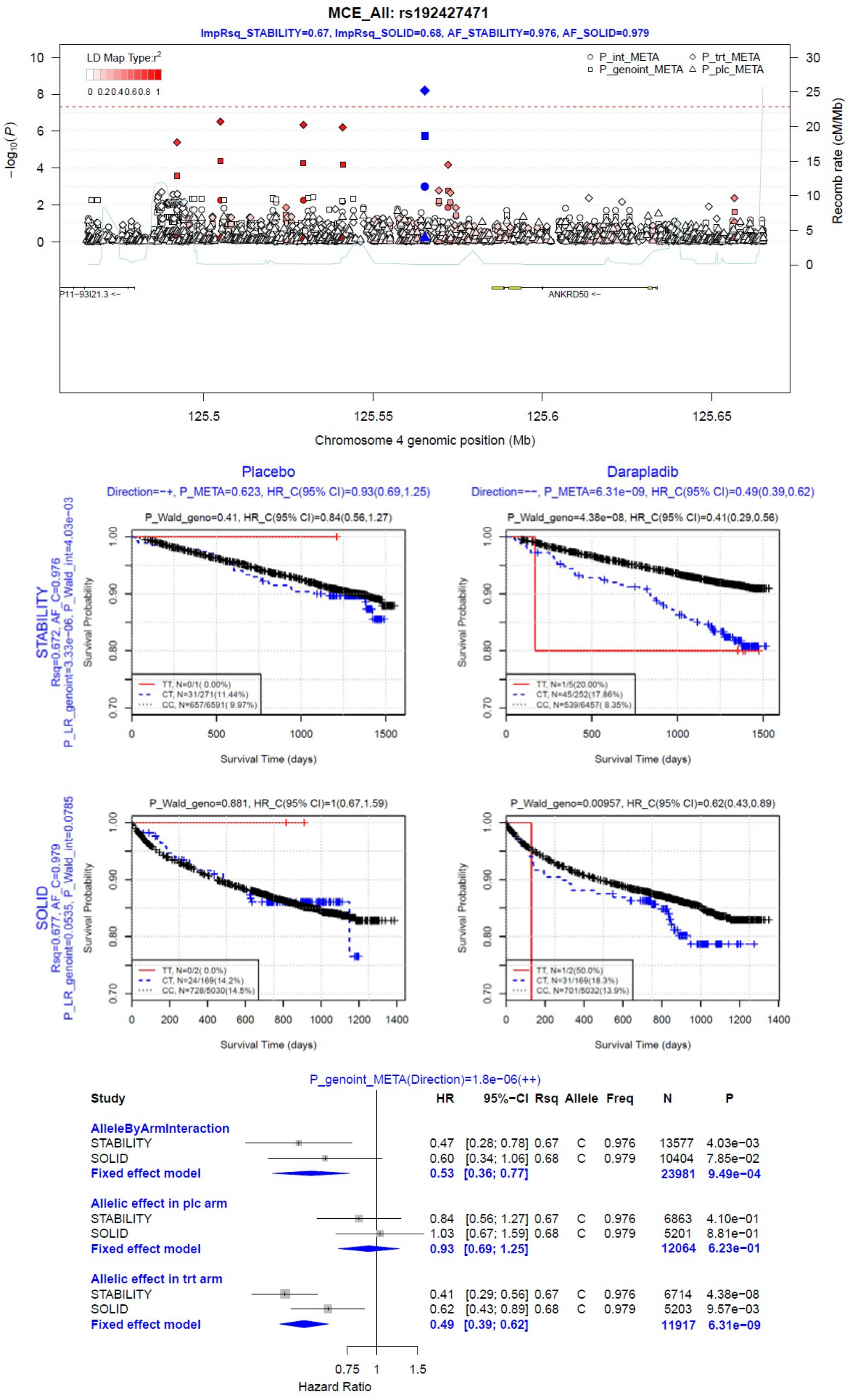
MCE_All: rs192427471
ImpRsq_STABILITY=0.67, ImpRsq_SOLID=0.68, AF_STABILITY=0.976, AF_SOLID=0.979
LD Map Type:r2
0 0.20.40.60.8 1
P_int_META
P_genoint_META
P_trt_META
P_plc_META
−log10(P)
Recomb rate (cM/Mb)
P11−93I21.3 <−
ANKRD50 <−
125.5
125.55
125.6
125.65
Chromosome 4 genomic position (Mb)
Placebo
Direction=−+, P_META=0.623, HR_C(95% CI)=0.93(0.69,1.25)
Darapladib
Direction=−−, P_META=6.31e−09, HR_C(95% CI)=0.49(0.39,0.62)
STABILITY
Rsq=0.672, AF_C=0.976
P_LR_genoint=3.33e−06, P_Wald_int=4.03e−03
P_Wald_geno=0.41, HR_C(95% CI)=0.84(0.56,1.27)
TT, N=0/1(0.00%)
CT, N=31/271(11.44%)
CC, N=657/6591(9.97%)
P_Wald_geno=4.38e−08, HR_C(95% CI)=0.41(0.29,0.56)
TT, N=1/5(20.00%)
CT, N=45/252(17.86%)
CC, N=539/6457(8.35%)
Survival Probability
Survival Time (days)
SOLID
Rsq=0.677, AF_C=0.979
P_LR_genoint=0.0535, P_Wald_int=0.0785
P_Wald_geno=0.881, HR_C(95% CI)=1(0.67,1.59)
TT, N=0/2(0.0%)
CT, N=24/169(14.2%)
CC, N=728/5030(14.5%)
P_Wald_geno=0.00957, HR_C(95% CI)=0.62(0.43,0.89)
TT, N=1/2(50.0%)
CT, N=31/169(18.3%)
CC, N=701/5032(13.9%)
P_genoint_META(Direction)=1.8e−06(++)
Study HR 95%−CI Rsq Allele Freq N P
AlleleByArmInteraction
STABILITY 0.47 [0.28; 0.78] 0.67 C 0.976 13577 4.03e−03
SOLID 0.60 [0.34; 1.06] 0.68 C 0.979 10404 7.85e−02
Fixed effect model 0.53 [0.36; 0.77] 23981 9.49e−04
Allelic effect in plc arm
STABILITY 0.84 [0.56; 1.27] 0.67 C 0.976 6863 4.10e−01
SOLID 1.03 [0.67; 1.59] 0.68 C 0.979 5201 8.81e−01
Fixed effect model 0.93 [0.69; 1.25] 12064 6.23e−01
Allelic effect in trt arm
STABILITY 0.41 [0.29; 0.56] 0.67 C 0.976 6714 4.38e−08
SOLID 0.62 [0.43; 0.89] 0.68 C 0.979 5203 9.57e−03
Fixed effect model 0.49 [0.39; 0.62] 11917 6.31e−09
0.75 1 1.5
Hazard Ratio

S5 Fig. c

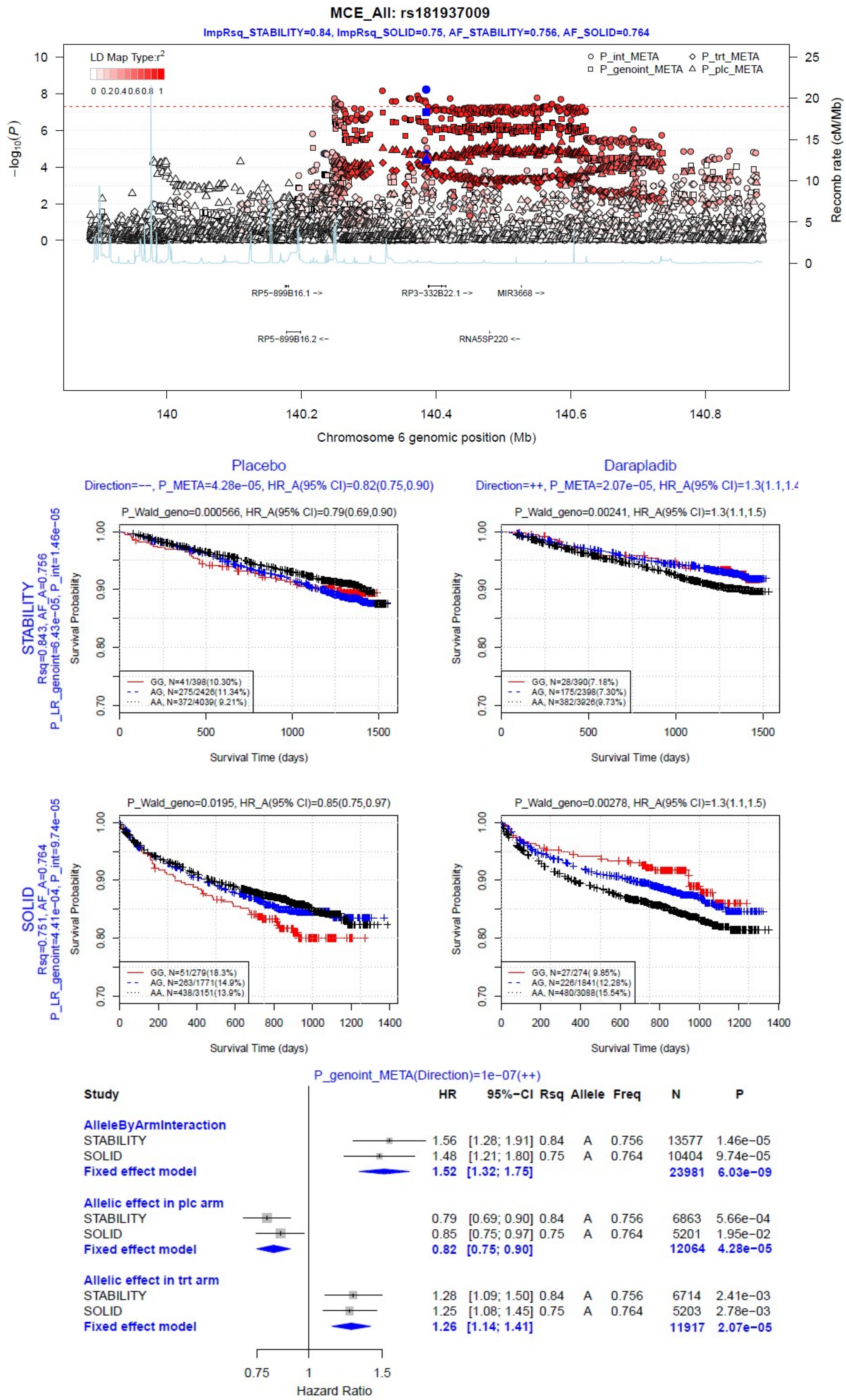
MCE_All: rs181937009
ImpRsq_STABILITY=0.84, ImpRsq_SOLID=0.75, AF_STABILITY=0.756, AF_SOLID=0.764
LD Map Type:r2
P_int_META
P_trt_META
P_genoint_META
P_plc_META
−log10(P)
Recomb rate (cM/Mb)
RP5−899B16.1 −>
RP3−332B22.1 −>
MIR3668 −>
RP5−899B16.2 <−
RNA5SP220 <−
Chromosome 6 genomic position (Mb)
Placebo
Direction=−−, P_META=4.28e−05, HR_A(95% CI)=0.82(0.75,0.90)
Darapladib
Direction=++, P_META=2.07e−05, HR_A(95% CI)=1.3(1.1,1.4
STABILITY
Rsq=0.843, AF_A=0.756
P_LR_genoint=6.43e−05, P_int=1.46e−05
P_Wald_geno=0.000566, HR_A(95% CI)=0.79(0.69,0.90)
GG, N=41/398(10.30%)
AG, N=275/2426(11.34%)
AA, N=372/4039(9.21%)
P_Wald_geno=0.00241, HR_A(95% CI)=1.3(1.1,1.5)
GG, N=28/390(7.18%)
AG, N=175/2398(7.30%)
AA, N=382/3926(9.73%)
Survival Probability
Survival Time (days)
SOLID
Rsq=0.751, AF_A=0.764
P_LR_genoint=4.41e−04, P_int=9.74e−05
P_Wald_geno=0.0195, HR_A(95% CI)=0.85(0.75,0.97)
GG, N=51/279(18.3%)
AG, N=263/1771(14.9%)
AA, N=438/3151(13.9%)
P_Wald_geno=0.00278, HR_A(95% CI)=1.3(1.1,1.5)
GG, N=27/274(9.85%)
AG, N=226/1841(12.28%)
AA, N=480/3088(15.54%)
P_genoint_META(Direction)=1e−07(++)
Study HR 95%−CI Rsq Allele Freq N P
AlleleByArmInteraction
STABILITY 1.56 [1.28; 1.91] 0.84 A 0.756 13577 1.46e−05
SOLID 1.48 [1.21; 1.80] 0.75 A 0.764 10404 9.74e−05
Fixed effect model 1.52 [1.32; 1.75] 23981 6.03e−09
Allelic effect in plc arm
STABILITY 0.79 [0.69; 0.90] 0.84 A 0.756 6863 5.66e−04
SOLID 0.85 [0.75; 0.97] 0.75 A 0.764 5201 1.95e−02
Fixed effect model 0.82 [0.75; 0.90] 12064 4.28e−05
Allelic effect in trt arm
STABILITY 1.28 [1.09; 1.50] 0.84 A 0.756 6714 2.41e−03
SOLID 1.25 [1.08; 1.45] 0.75 A 0.764 5203 2.78e−03
Fixed effect model 1.26 [1.14; 1.41] 11917 2.07e−05
Hazard Ratio

S5 Fig. d

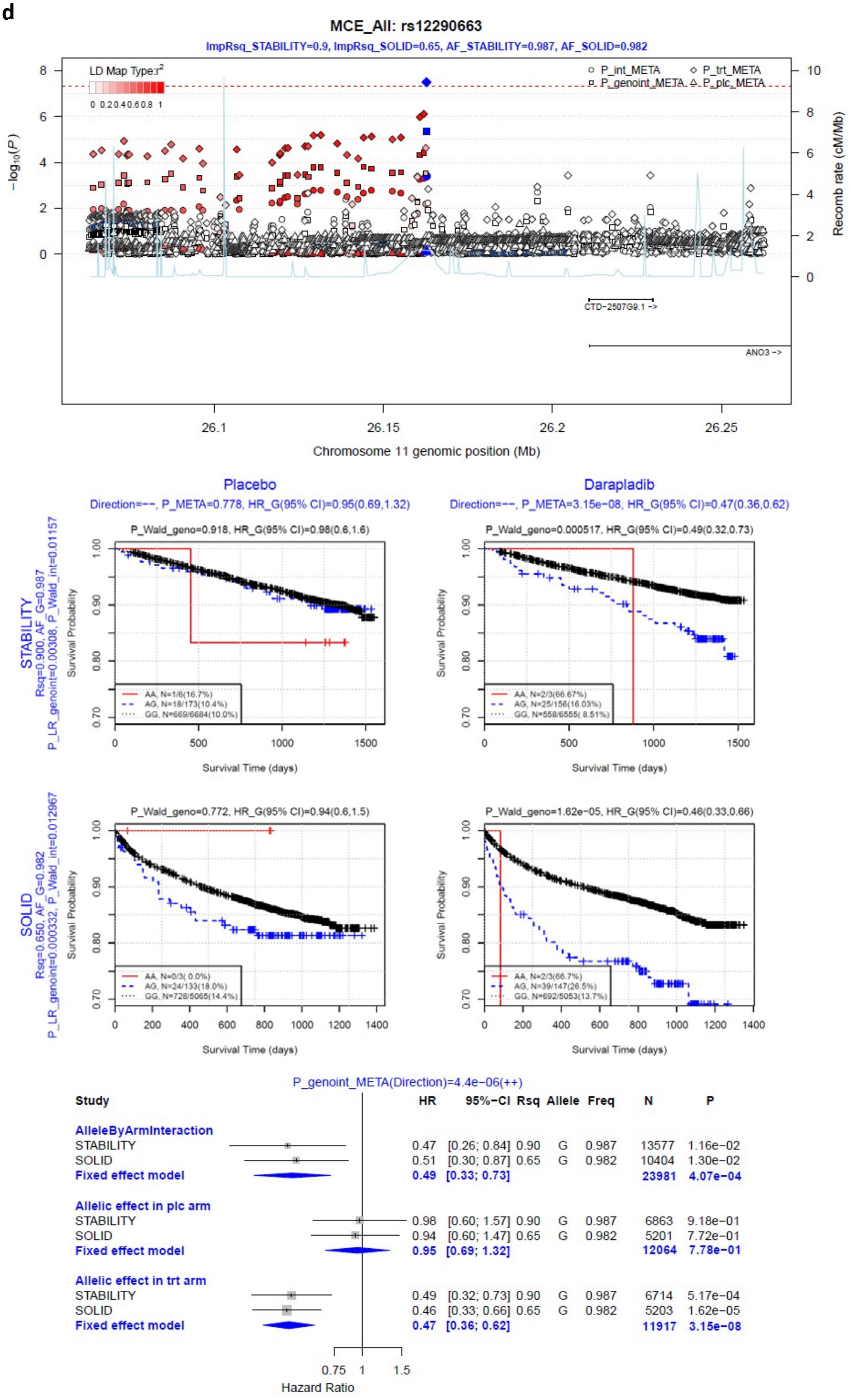
MCE_All: rs12290663
ImpRsq_STABILITY=0.9, ImpRsq_SOLID=0.65, AF_STABILITY=0.987, AF_SOLID=0.982
LD Map Type:r²
0 0.2 0.4 0.6 0.8 1
P_int_META
P_trt_META
P_genoint_META
P_plc_META
−log10(P)
Recomb rate (cM/Mb)
CTD−2507G9.1 −>
ANO3 −>
26.1
26.15
26.2
26.25
Chromosome 11 genomic position (Mb)
Placebo
Direction=−−, P_META=0.778, HR_G(95% CI)=0.95(0.69,1.32)
Darapladib
Direction=−−, P_META=3.15e−08, HR_G(95% CI)=0.47(0.36,0.62)
STABILITY
Rsq=0.900, AF_G=0.987
P_LR_genoint=0.00308, P_Wald_int=0.01157
P_Wald_geno=0.918, HR_G(95% CI)=0.98(0.6,1.6)
AA, N=1/6(16.7%)
AG, N=18/173(10.4%)
GG, N=669/6684(10.0%)
P_Wald_geno=0.000517, HR_G(95% CI)=0.49(0.32,0.73)
AA, N=2/3(66.67%)
AG, N=25/156(16.03%)
GG, N=558/6555(8.51%)
Survival Probability
Survival Time (days)
SOLID
Rsq=0.650, AF_G=0.982
P_LR_genoint=0.000332, P_Wald_int=0.012967
P_Wald_geno=0.772, HR_G(95% CI)=0.94(0.6,1.5)
AA, N=0/3(0.0%)
AG, N=24/133(18.0%)
GG, N=728/5065(14.4%)
P_Wald_geno=1.62e−05, HR_G(95% CI)=0.46(0.33,0.66)
AA, N=2/3(66.7%)
AG, N=39/147(26.5%)
GG, N=692/5053(13.7%)
P_genoint_META(Direction)=4.4e−06(++)
Study HR 95%−CI Rsq Allele Freq N P
AlleleByArmInteraction
STABILITY 0.47 [0.26; 0.84] 0.90 G 0.987 13577 1.16e−02
SOLID 0.51 [0.30; 0.87] 0.65 G 0.982 10404 1.30e−02
Fixed effect model 0.49 [0.33; 0.73] 23981 4.07e−04
Allelic effect in plc arm
STABILITY 0.98 [0.60; 1.57] 0.90 G 0.987 6863 9.18e−01
SOLID 0.94 [0.60; 1.47] 0.65 G 0.982 5201 7.72e−01
Fixed effect model 0.95 [0.69; 1.32] 12064 7.78e−01
Allelic effect in trt arm
STABILITY 0.49 [0.32; 0.73] 0.90 G 0.987 6714 5.17e−04
SOLID 0.46 [0.33; 0.66] 0.65 G 0.982 5203 1.62e−05
Fixed effect model 0.47 [0.36; 0.62] 11917 3.15e−08
0.75 1 1.5
Hazard Ratio

S5 Fig. e

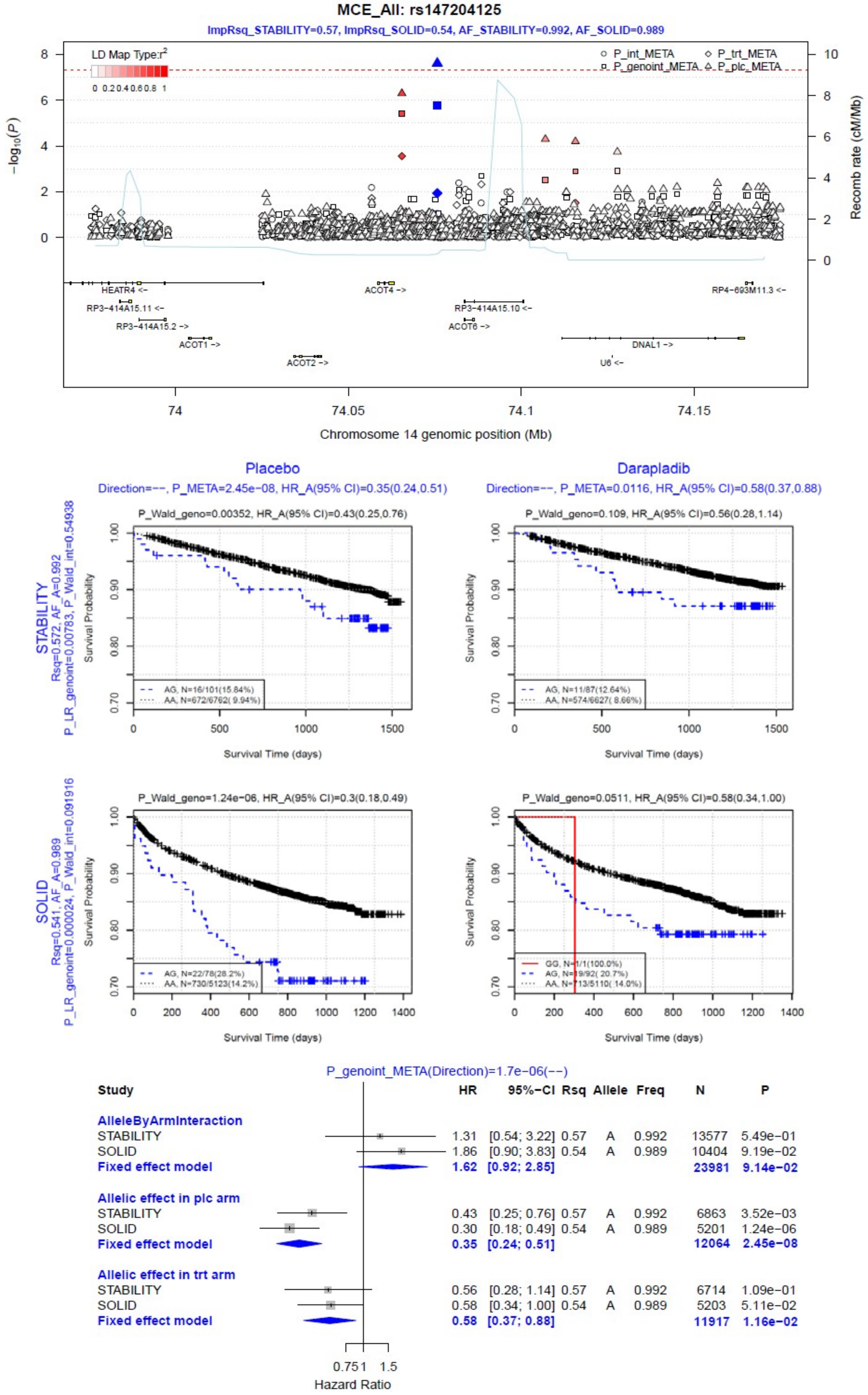

MCE_All: rs147204125
ImpRsq_STABILITY=0.57, ImpRsq_SOLID=0.54, AF_STABILITY=0.992, AF_SOLID=0.989
LD Map Type:r²
P_int_META
P_trt_META
P_genoint_META
P_plc_META
−log10(P)
Recomb rate (cM/Mb)
HEATR4 <-
RP3-414A15.11 <-
RP3-414A15.2 ->
ACOT1 ->
ACOT2 ->
ACOT4 ->
RP3-414A15.10 <-
ACOT6 ->
DNAL1 ->
U6 <-
RP4-693M11.3 <-
Chromosome 14 genomic position (Mb)
Placebo
Direction=−−, P_META=2.45e−08, HR_A(95% CI)=0.35(0.24,0.51)
Darapladib
Direction=−−, P_META=0.0116, HR_A(95% CI)=0.58(0.37,0.88)
STABILITY
Rsq=0.572, AF_A=0.992
P_LR_genoint=0.00783, P_Wald_int=0.54938
P_Wald_geno=0.00352, HR_A(95% CI)=0.43(0.25,0.76)
AG, N=16/101(15.84%)
AA, N=672/6762(9.94%)
P_Wald_geno=0.109, HR_A(95% CI)=0.56(0.28,1.14)
AG, N=11/87(12.64%)
AA, N=574/6627(8.66%)
SOLID
Rsq=0.541, AF_A=0.989
P_LR_genoint=0.000024, P_Wald_int=0.091916
P_Wald_geno=1.24e−06, HR_A(95% CI)=0.3(0.18,0.49)
AG, N=22/78(28.2%)
AA, N=730/5123(14.2%)
P_Wald_geno=0.0511, HR_A(95% CI)=0.58(0.34,1.00)
GG, N=1/1(100.0%)
AG, N=19/92(20.7%)
AA, N=713/5110(14.0%)
Survival Probability
Survival Time (days)
P_genoint_META(Direction)=1.7e−06(−−)
Study HR 95%−CI Rsq Allele Freq N P
AlleleByArmInteraction
STABILITY 1.31 [0.54; 3.22] 0.57 A 0.992 13577 5.49e−01
SOLID 1.86 [0.90; 3.83] 0.54 A 0.989 10404 9.19e−02
Fixed effect model 1.62 [0.92; 2.85] 23981 9.14e−02
Allelic effect in plc arm
STABILITY 0.43 [0.25; 0.76] 0.57 A 0.992 6863 3.52e−03
SOLID 0.30 [0.18; 0.49] 0.54 A 0.989 5201 1.24e−06
Fixed effect model 0.35 [0.24; 0.51] 12064 2.45e−08
Allelic effect in trt arm
STABILITY 0.56 [0.28; 1.14] 0.57 A 0.992 6714 1.09e−01
SOLID 0.58 [0.34; 1.00] 0.54 A 0.989 5203 5.11e−02
Fixed effect model 0.58 [0.37; 0.88] 11917 1.16e−02
Hazard Ratio
