# Supplementary material for: Pharmacogenetic meta-analysis of baseline risk factors, pharmacodynamic, efficacy and tolerability endpoints from two large global cardiovascular outcomes trials for darapladib
Source: PLoS One. 2017 Jul 28;12(7):e0182115. doi: 10.1371/journal.pone.0182115 (PMC5533343; doi:10.1371/journal.pone.0182115)
Supplement: S6 Fig — The dashed line in the regional plots indicates the significance threshold. a) rs192476688, rs201052613, b) rs117714106. (PDF) [file pone.0182115.s007.pdf]

S6 Fig. Regional, survival and forest plots of variants associated with MI following placebo and darapladib treatment. The dashed line in the regional plots indicates the significance threshold. a) rs192476688, rs201052613, b) rs117714106.

S6 Fig. a

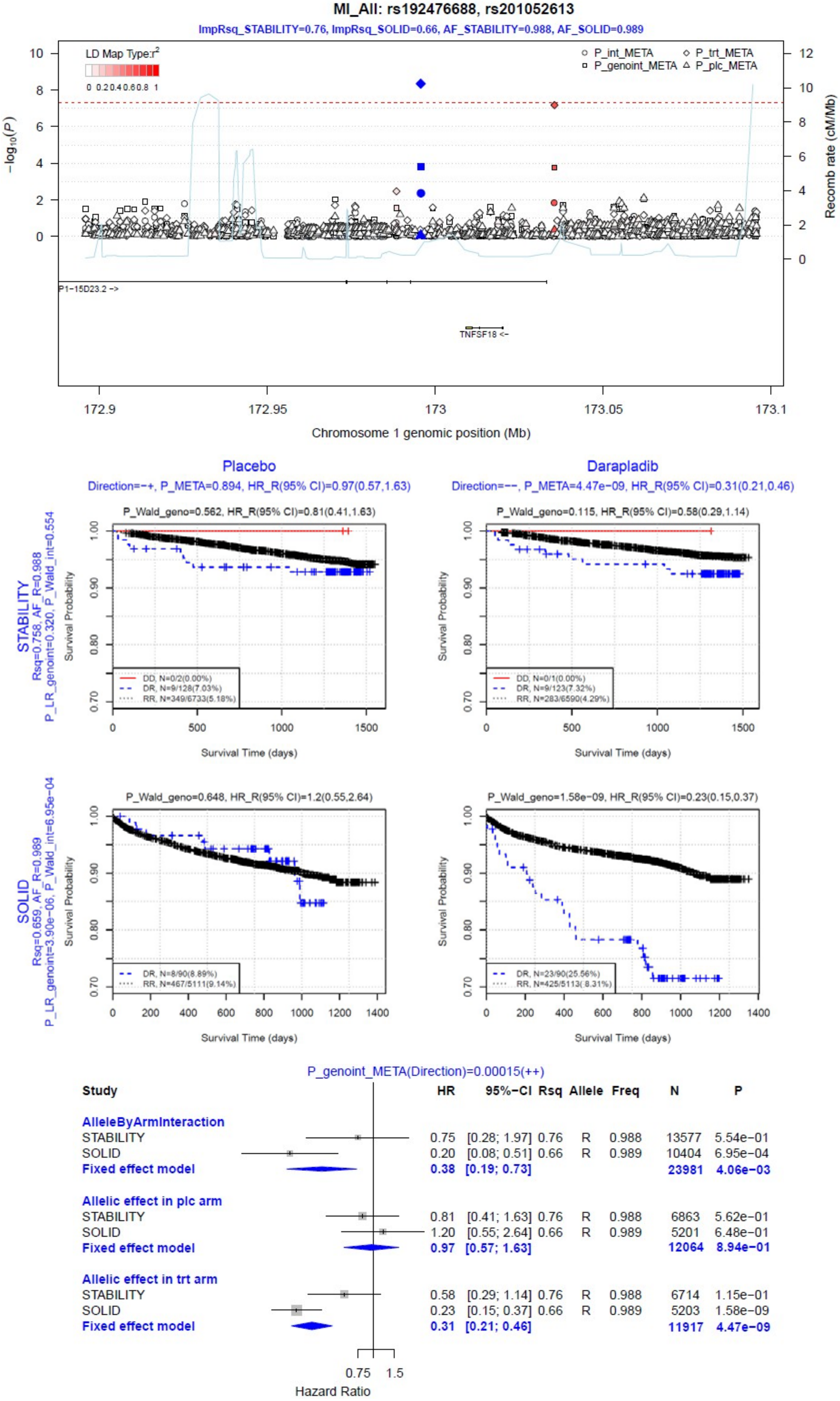
MI_All: rs192476688, rs201052613
ImpRsq_STABILITY=0.76, ImpRsq_SOLID=0.66, AF_STABILITY=0.988, AF_SOLID=0.989
LD Map Type:r²
P_int_META
P_genoint_META
P_trt_META
P_plc_META
−log10(P)
Recomb rate (cM/Mb)
P1-15D23.2 ->
TNFSF18 <-
Chromosome 1 genomic position (Mb)
Placebo
Direction=-+, P_META=0.894, HR_R(95% CI)=0.97(0.57,1.63)
Darapladib
Direction=--, P_META=4.47e-09, HR_R(95% CI)=0.31(0.21,0.46)
STABILITY
Rsq=0.758, AF_R=0.988
P_LR_genoint=0.320, P_Wald_int=0.554
P_Wald_geno=0.562, HR_R(95% CI)=0.81(0.41,1.63)
DD, N=0/2(0.00%)
DR, N=9/128(7.03%)
RR, N=349/6733(5.18%)
P_Wald_geno=0.115, HR_R(95% CI)=0.58(0.29,1.14)
DD, N=0/1(0.00%)
DR, N=9/123(7.32%)
RR, N=283/6590(4.29%)
SOLID
Rsq=0.659, AF_R=0.989
P_LR_genoint=3.90e-06, P_Wald_int=6.95e-04
P_Wald_geno=0.648, HR_R(95% CI)=1.2(0.55,2.64)
DR, N=8/90(8.89%)
RR, N=467/5111(9.14%)
P_Wald_geno=1.58e-09, HR_R(95% CI)=0.23(0.15,0.37)
DR, N=23/90(25.56%)
RR, N=425/5113(8.31%)
Survival Probability
Survival Time (days)
P_genoint_META(Direction)=0.00015(++)
Study HR 95%-CI Rsq Allele Freq N P
AlleleByArmInteraction
STABILITY 0.75 [0.28; 1.97] 0.76 R 0.988 13577 5.54e-01
SOLID 0.20 [0.08; 0.51] 0.66 R 0.989 10404 6.95e-04
Fixed effect model 0.38 [0.19; 0.73] 23981 4.06e-03
Allelic effect in plc arm
STABILITY 0.81 [0.41; 1.63] 0.76 R 0.988 6863 5.62e-01
SOLID 1.20 [0.55; 2.64] 0.66 R 0.989 5201 6.48e-01
Fixed effect model 0.97 [0.57; 1.63] 12064 8.94e-01
Allelic effect in trt arm
STABILITY 0.58 [0.29; 1.14] 0.76 R 0.988 6714 1.15e-01
SOLID 0.23 [0.15; 0.37] 0.66 R 0.989 5203 1.58e-09
Fixed effect model 0.31 [0.21; 0.46] 11917 4.47e-09
0.75 1.5
Hazard Ratio

S6 Fig. b

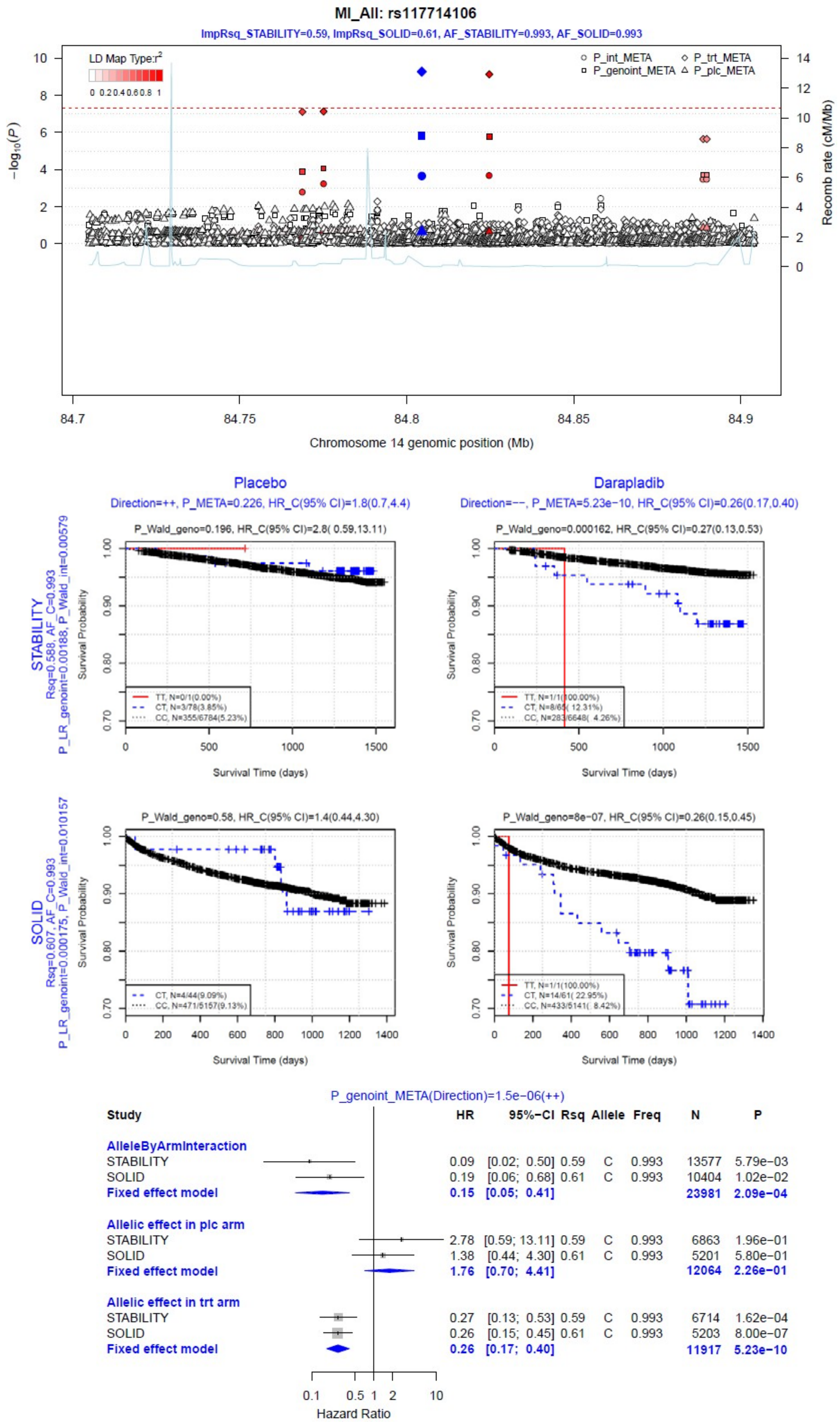
MI_All: rs117714106
ImpRsq_STABILITY=0.59, ImpRsq_SOLID=0.61, AF_STABILITY=0.993, AF_SOLID=0.993
LD Map Type:r2
0 0.2 0.4 0.6 0.8 1
P_int_META
P_genoint_META
P_trt_META
P_plc_META
−log10(P)
Recomb rate (cM/Mb)
Chromosome 14 genomic position (Mb)
Placebo
Direction=++, P_META=0.226, HR_C(95% CI)=1.8(0.7,4.4)
Darapladib
Direction=−−, P_META=5.23e-10, HR_C(95% CI)=0.26(0.17,0.40)
STABILITY
Rsq=0.588, AF_C=0.993
P_LR_genoint=0.00188, P_Wald_int=0.00579
P_Wald_geno=0.196, HR_C(95% CI)=2.8(0.59,13.11)
TT, N=0/1(0.00%)
CT, N=3/78(3.85%)
CC, N=355/6784(5.23%)
P_Wald_geno=0.000162, HR_C(95% CI)=0.27(0.13,0.53)
TT, N=1/1(100.00%)
CT, N=8/65(12.31%)
CC, N=283/6648(4.26%)
SOLID
Rsq=0.607, AF_C=0.993
P_LR_genoint=0.000175, P_Wald_int=0.010157
P_Wald_geno=0.58, HR_C(95% CI)=1.4(0.44,4.30)
CT, N=4/44(9.09%)
CC, N=471/5157(9.13%)
P_Wald_geno=8e-07, HR_C(95% CI)=0.26(0.15,0.45)
TT, N=1/1(100.00%)
CT, N=14/61(22.95%)
CC, N=433/5141(8.42%)
Survival Probability
Survival Time (days)
P_genoint_META(Direction)=1.5e-06(++)
Study HR 95%-CI Rsq Allele Freq N P
AlleleByArmInteraction
STABILITY 0.09 [0.02; 0.50] 0.59 C 0.993 13577 5.79e-03
SOLID 0.19 [0.06; 0.68] 0.61 C 0.993 10404 1.02e-02
Fixed effect model 0.15 [0.05; 0.41] 23981 2.09e-04
Allelic effect in plc arm
STABILITY 2.78 [0.59; 13.11] 0.59 C 0.993 6863 1.96e-01
SOLID 1.38 [0.44; 4.30] 0.61 C 0.993 5201 5.80e-01
Fixed effect model 1.76 [0.70; 4.41] 12064 2.26e-01
Allelic effect in trt arm
STABILITY 0.27 [0.13; 0.53] 0.59 C 0.993 6714 1.62e-04
SOLID 0.26 [0.15; 0.45] 0.61 C 0.993 5203 8.00e-07
Fixed effect model 0.26 [0.17; 0.40] 11917 5.23e-10
Hazard Ratio
