# Supplementary material for: Pharmacogenetic meta-analysis of baseline risk factors, pharmacodynamic, efficacy and tolerability endpoints from two large global cardiovascular outcomes trials for darapladib
Source: PLoS One. 2017 Jul 28;12(7):e0182115. doi: 10.1371/journal.pone.0182115 (PMC5533343; doi:10.1371/journal.pone.0182115)
Supplement: S7 Fig — a) diarrhea in darapladib arm, b) moderate and severe diarrhea in darapladib arm, c) bathroom relate odor events in darapladib arm, d) non-bathroom relate odor events in darapladib arm. (PDF) [file pone.0182115.s008.pdf]

S7 Fig. Manhattan, QQ and histogram plots for tolerability endpoints. a) diarrhea in darapladib arm, b) moderate and severe diarrhea in darapladib arm, c) bathroom relate odor events in darapladib arm, d) non-bathroom relate odor events in darapladib arm.

a

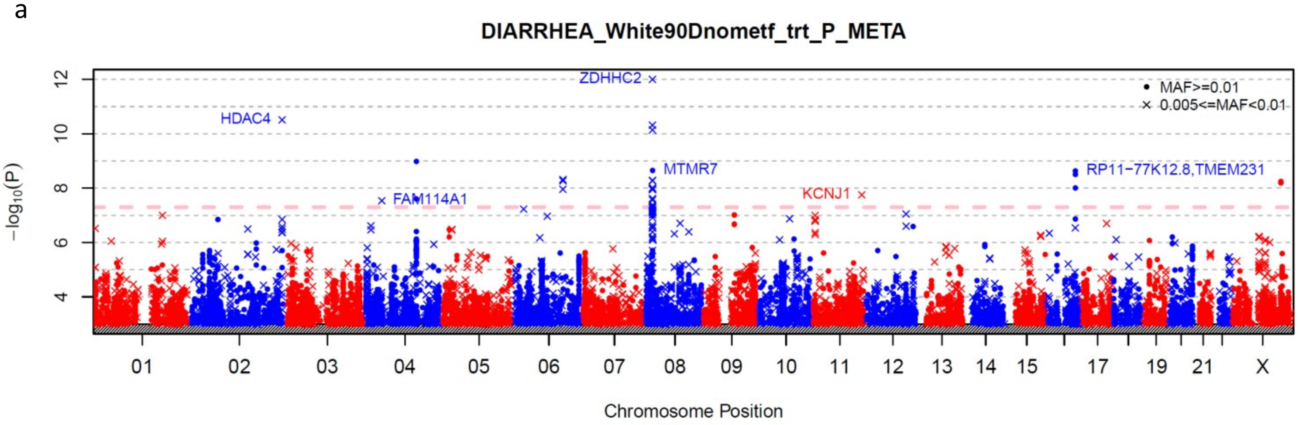

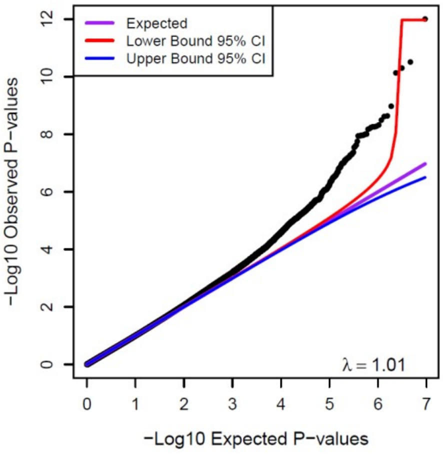

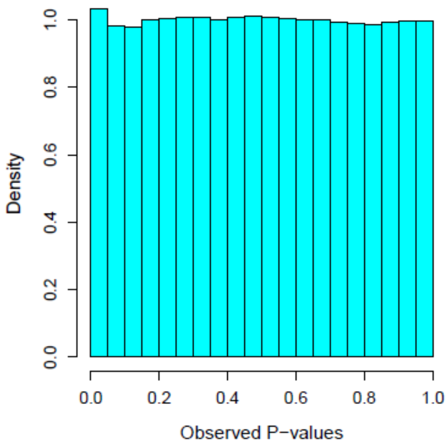

S7 Fig. b

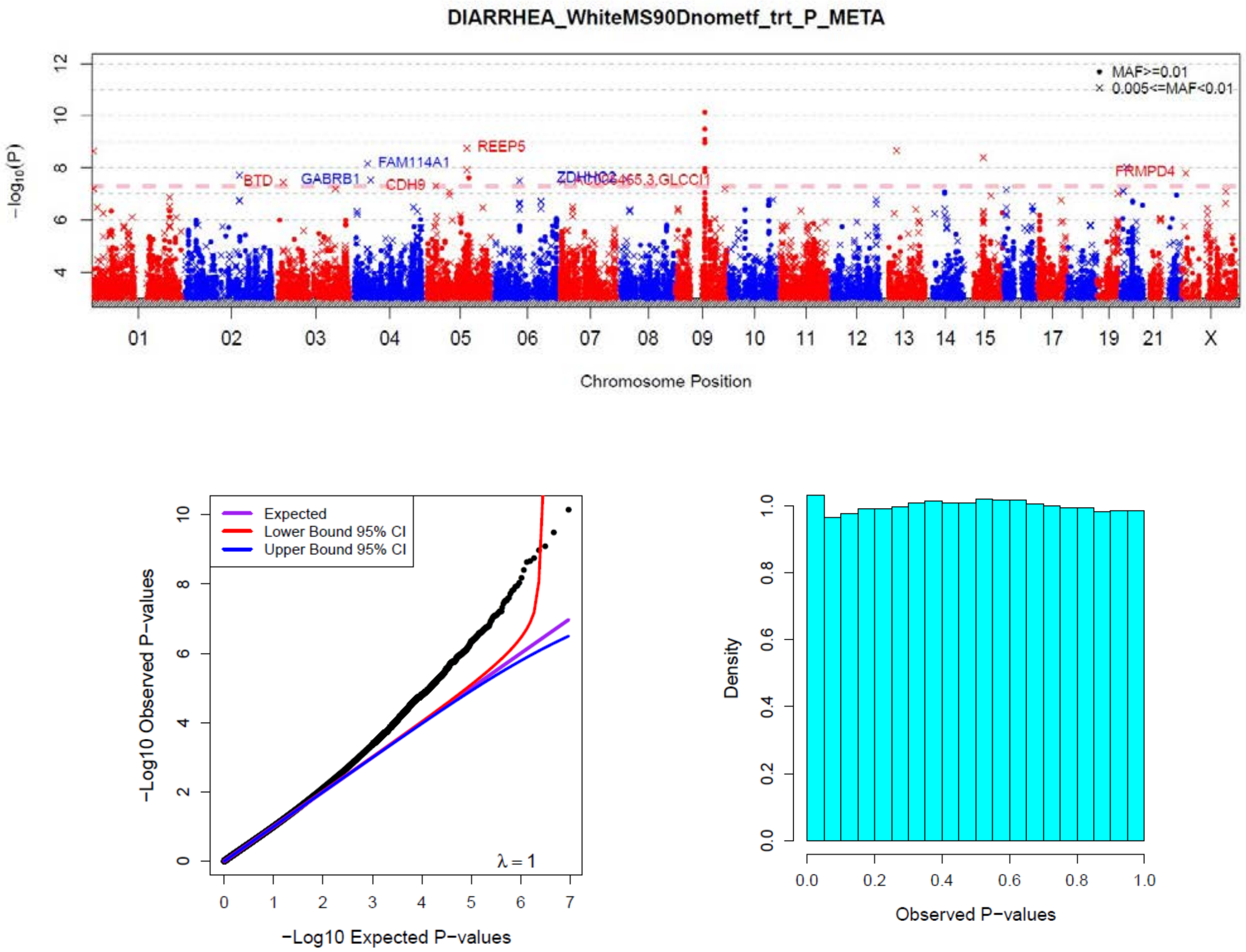
DIARRHEA_WhiteMS90Dnometf_trt_P_META
MAF>=0.01
0.005<=MAF<0.01
BTD
GABRB1
FAM114A1
CDH9
REEP5
FRMPD4
−log10(P)
Chromosome Position
Expected
Lower Bound 95% CI
Upper Bound 95% CI
λ = 1
−Log10 Observed P−values
−Log10 Expected P−values
Density
Observed P−values

S7 Fig. c

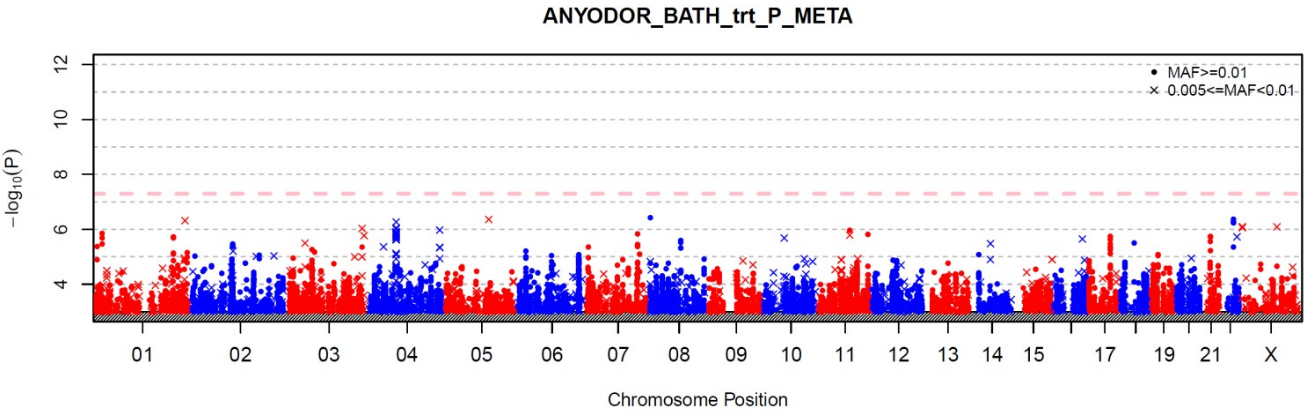

ANYODOR_BATH_trt_P_META
MAF>=0.01
0.005<=MAF<0.01
−log10(P)
12
10
8
6
4
01
02
03
04
05
06
07
08
09
10
11
12
13
14
15
17
19
21
X
Chromosome Position

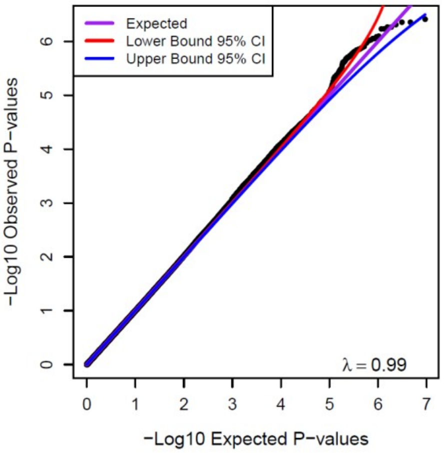

Expected
Lower Bound 95% CI
Upper Bound 95% CI
λ = 0.99
−Log10 Observed P−values
−Log10 Expected P−values
0
1
2
3
4
5
6
7

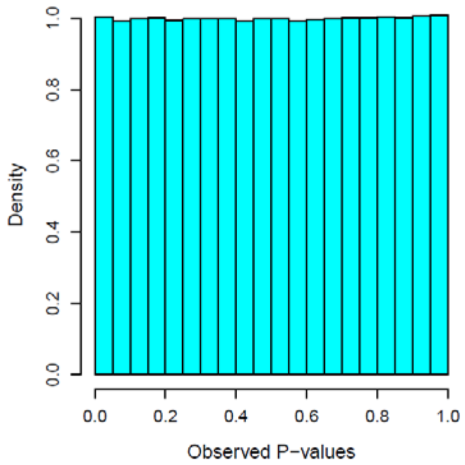

Density
Observed P−values
0.0
0.2
0.4
0.6
0.8
1.0

S7 Fig. d

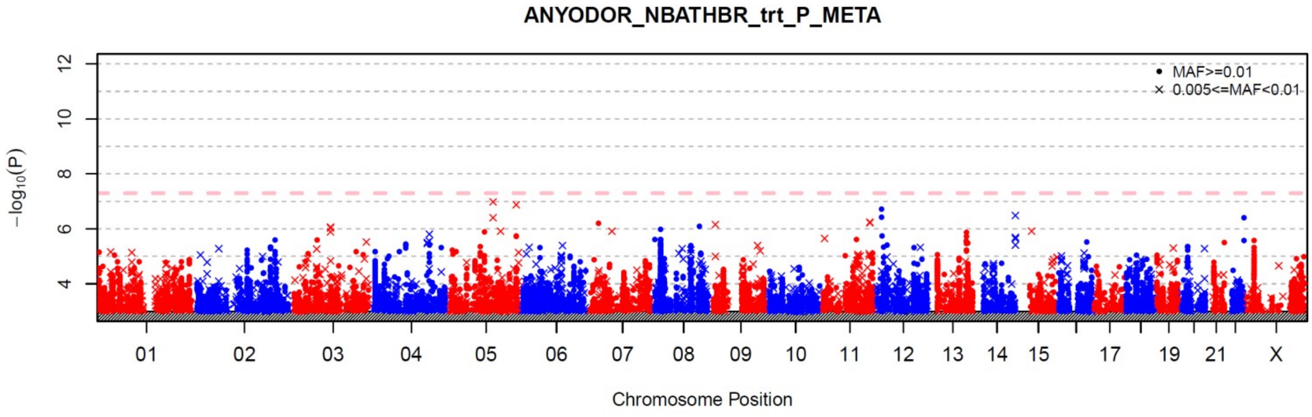
ANYODOR_NBATHBR_trt_P_META
MAF>=0.01
0.005<=MAF<0.01
-log10(P)
12
10
8
6
4
01
02
03
04
05
06
07
08
09
10
11
12
13
14
15
17
19
21
X
Chromosome Position

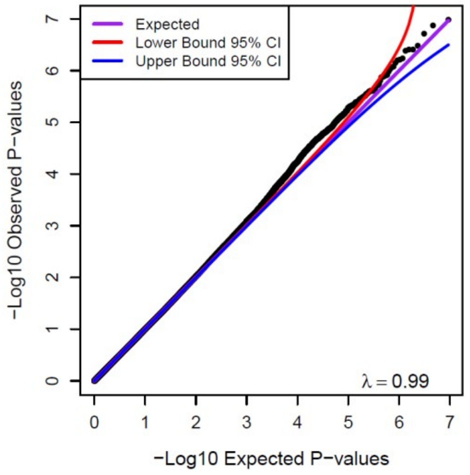
Expected
Lower Bound 95% CI
Upper Bound 95% CI
−Log10 Observed P−values
−Log10 Expected P−values
λ = 0.99
0
1
2
3
4
5
6
7

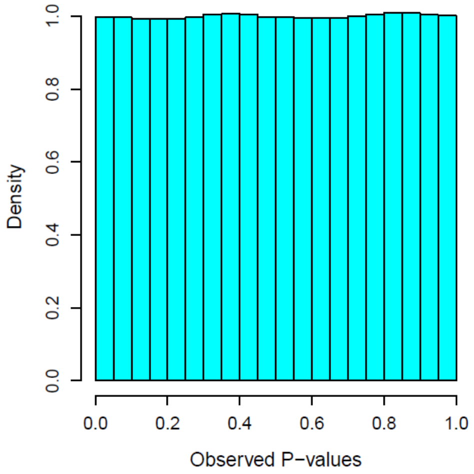
Density
Observed P−values
0.0
0.2
0.4
0.6
0.8
1.0
