# Supplementary material for: Pharmacogenetic meta-analysis of baseline risk factors, pharmacodynamic, efficacy and tolerability endpoints from two large global cardiovascular outcomes trials for darapladib
Source: PLoS One. 2017 Jul 28;12(7):e0182115. doi: 10.1371/journal.pone.0182115 (PMC5533343; doi:10.1371/journal.pone.0182115)

S8 Fig. Heatmap of p-values observed for the genome-wide significant variants for Lp-PLA_2 enzyme activity, efficacy and tolerability endpoints.

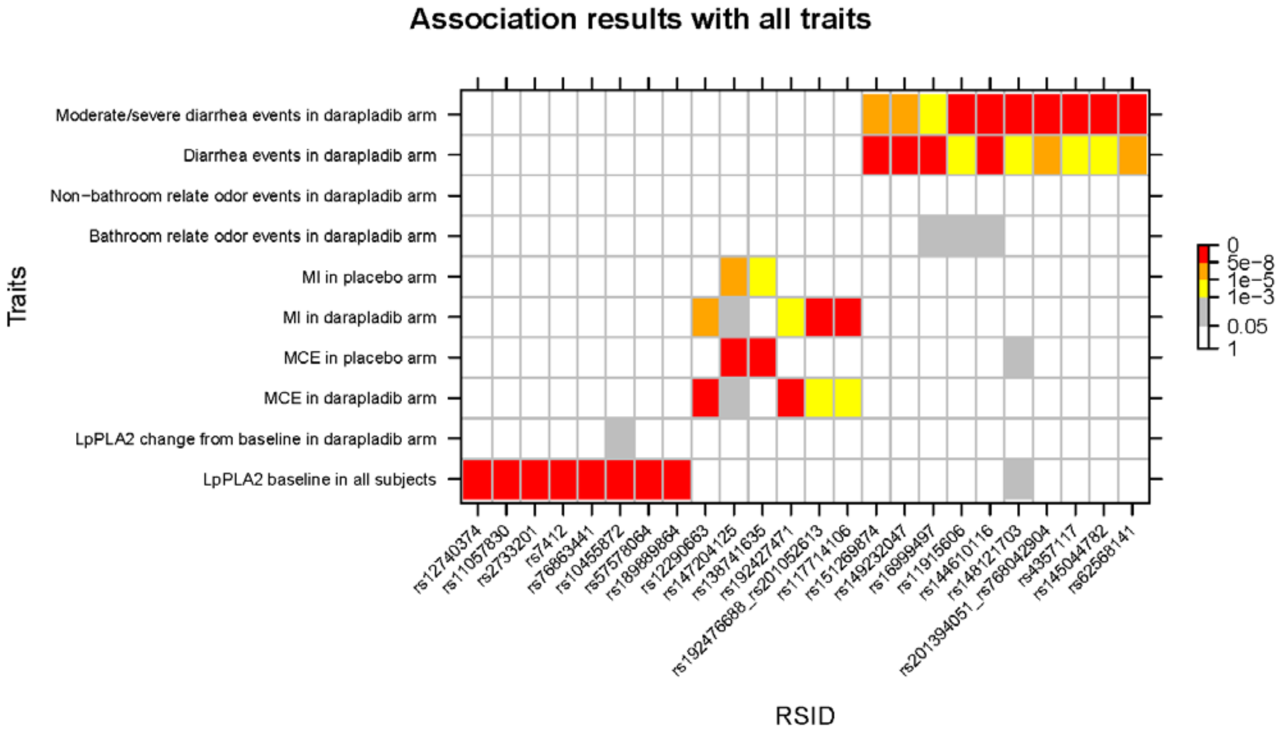

Supplement: S8 Fig — (PDF) [file pone.0182115.s009.pdf]
