# Supplementary material for: Pharmacogenetic meta-analysis of baseline risk factors, pharmacodynamic, efficacy and tolerability endpoints from two large global cardiovascular outcomes trials for darapladib
Source: PLoS One. 2017 Jul 28;12(7):e0182115. doi: 10.1371/journal.pone.0182115 (PMC5533343; doi:10.1371/journal.pone.0182115)

S10 Fig. World map constructed using principle components from GWAS data of STABILITY subjects. Highlights the great diversity in country origin which is inherent to large clinical trials.

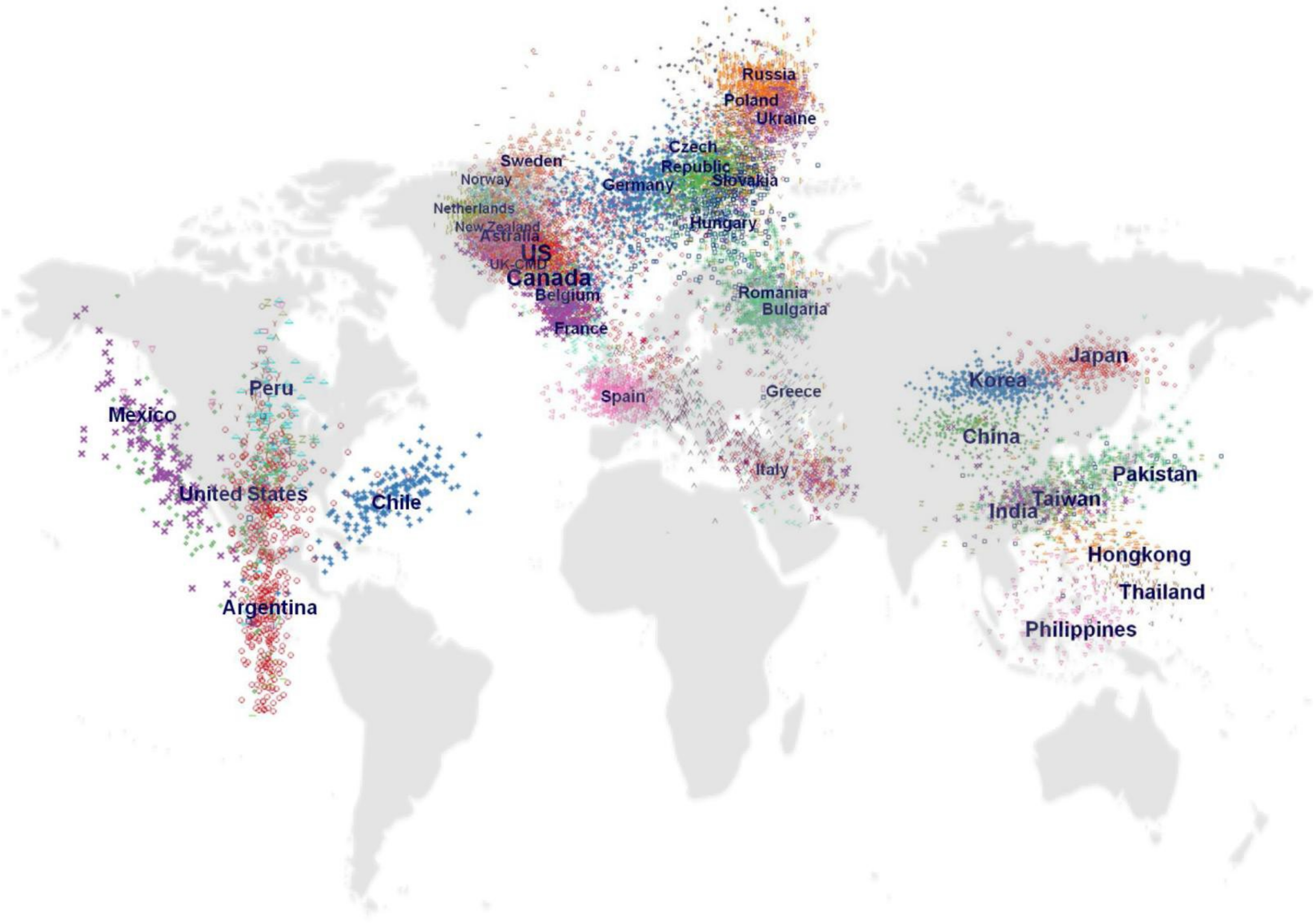

Supplement: S10 Fig — Highlights the great diversity in country origin which is inherent to large clinical trials. (PDF) [file pone.0182115.s011.pdf]
