# Supplementary material for: Pharmacogenetic meta-analysis of baseline risk factors, pharmacodynamic, efficacy and tolerability endpoints from two large global cardiovascular outcomes trials for darapladib
Source: PLoS One. 2017 Jul 28;12(7):e0182115. doi: 10.1371/journal.pone.0182115 (PMC5533343; doi:10.1371/journal.pone.0182115)

S11 Fig. Baseline Lp-PLA$_2$ activity level plots for low frequency and rare variants in the *PLA2G7* gene conditioned on V279F. Main plot shows association P-values (main plot), with effect estimates in top right insert.

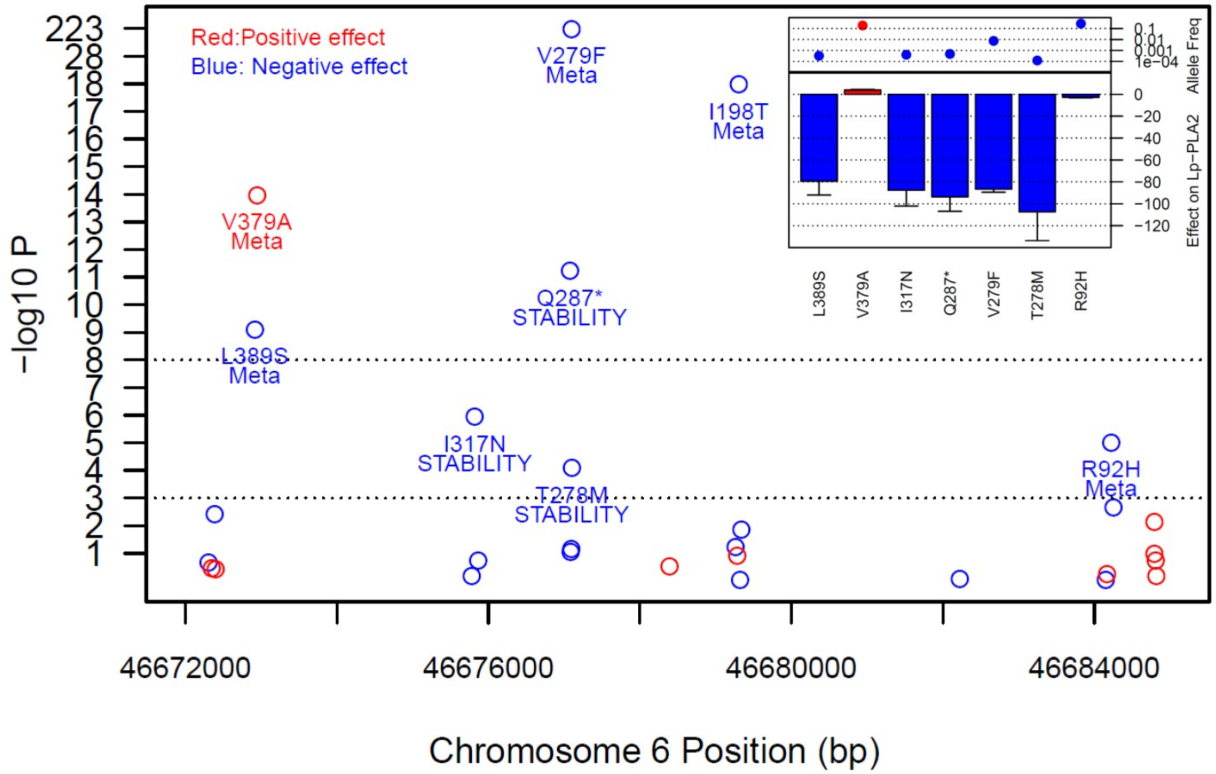

Supplement: S11 Fig — Main plot shows association P-values (main plot), with effect estimates in top right insert. (PDF) [file pone.0182115.s012.pdf]
